# Stereopermutation on the Putative Structure of the Marine Natural Product Mucosin

**DOI:** 10.3390/molecules22101720

**Published:** 2017-10-13

**Authors:** Simen G. Antonsen, Harrison Gallantree-Smith, Carl Henrik Görbitz, Trond Vidar Hansen, Yngve H. Stenstrøm, Jens M. J. Nolsøe

**Affiliations:** 1 Faculty of Chemistry, Biotechnology and Food Science, Norwegian University of Life Sciences, P.O. Box 5003, 1433 Ås, Norway; simen.antonsen@nmbu.no (S.G.A.); lithium_87@hotmail.co.uk (H.G.-S.); t.v.hansen@farmasi.uio.no (T.V.H.); yngve.stenstrom@nmbu.no (Y.H.S.); 2 Department of Chemistry, University of Oslo, P.O. Box 1033, 0315 Oslo, Norway; c.h.gorbitz@kjemi.uio.no; 3 Department of Pharmaceutical Chemistry, University of Oslo, P.O. Box 1068, 0316 Oslo, Norway

**Keywords:** marine hydrindane natural product, asymmetric synthesis, stereodivergent strategy, structural elucidation, eicosanoid

## Abstract

A stereodivergent total synthesis has been executed based on the plausibly misassigned structure of the unusual marine hydrindane mucosin (**1**). The topological connectivity of the four contiguous *all*-carbon stereocenters has been examined by selective permutation on the highlighted core. Thus, capitalizing on an unprecedented stereofacial preference of the *cis*-fused bicycle[4.3.0]non-3-ene system when a Michael acceptor motif is incorporated, copper-mediated conjugate addition furnished a single diastereomer. Cued by the relative relationship reported for the appendices in the natural product, the resulting *anti*-adduct was elaborated into a probative target structure 1*.

## 1. Introduction

Certain metabolites derived from polyunsaturated fatty acids (PUFAs) play a key role in mammalian physiology, where they orchestrate both inflammatory response as well as the return to homeostasis [1,2,3,4]. By combining total synthesis with chemical biology and molecular pharmacology, a number of distinct eicosanoids and docosanoids have been identified, which are active in the cascade elicited by noxious stimuli [5,6,7,8,9,10,11,12,13,14,15,16,17,18,19,20,21,22,23]. As a result, natural products with an underlying PUFA motif are of great interest as potential immunomodulators.

Since antiquity, sea dwelling organisms have proven to be a particularly abundant source of new chemical entities, set apart from those found in the terrestrial environment [24,25]. Thus, the ancient Phoenicians were renowned for their trading with Tyrian purple from the *Murex* sea snail [26,27]. Rising above mere prospecting, modern-day discovery, enabled by the advent of powerful analytical instruments and methods, has found a wealth of bioactive compounds in the marine environment [28,29,30,31,32,33,34].

**Figure 1:**
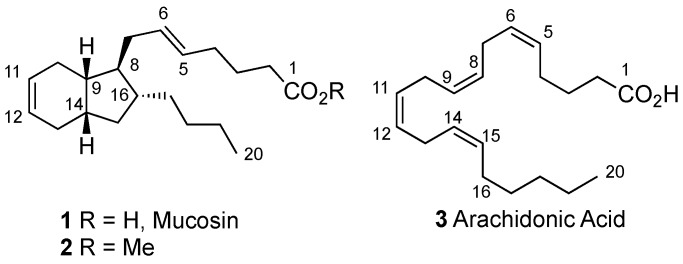
Suggested structure of mucosin and its relation to arachidonic acid.

Ostensibly, mucosin (**1**) is a natural product that was isolated from the Mediterranean sponge *Reniera mucosa* as methyl ester **2** [35]. Formally classified as an eicosanoid, it has been conjectured to originate from arachidonic acid (**3**), based on the C_20_-architechture (Figure 1). While sharing some noticeable features with the prostane scaffold, the compound differs by having an unusual bicyclic core. Clearly, in the structure proposed for mucosin (**1**), the characteristic cyclopentane ring is integrated in a *cis*-fused bicyclo[4.3.0]non-3-ene system. However, turning to the elucidation, the assignment of topology poses a challenge. Though only a small molecule, the structure is compact in terms of the four contiguous stereocentres. In NMR experiments on the isolated methyl ester, pertaining to both 1D- and 2D-techniques, the distinguishing resonances/correlations are ensconced in a crowded aliphatic region. Consequently, with the absence of any coupling pattern to corroborate the configuration of the carbocycle, the assignment published by Casapullo et al. does not convince on its own [35].

**Figure 2:**
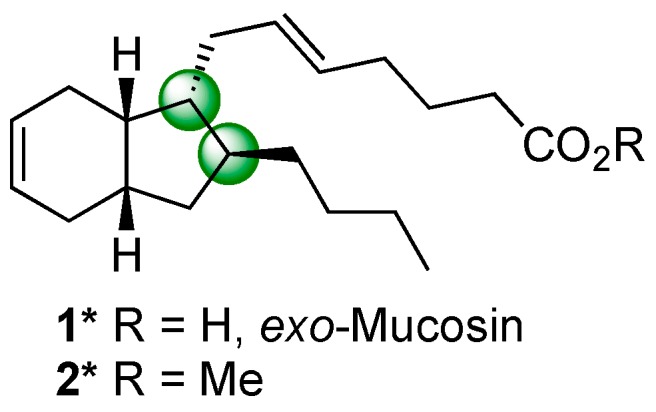
Stereopermutation on the *cis*-bicyclo[4.3.0]non-3-ene scaffold.

Fascinated by the structure and the prostane-like motif, we devised a practical, divergent and synthetically unambiguous strategy to establish the proposed stereochemistry. At the end of the campaign, capitalizing on X-ray crystallography to pinpoint the relative arrangement, it was concluded that mucosin (**1**) does not represent the portrayed compound [36]. In a pursuit to identify the natural product isolated from *Reniera mucosa*, our intent is to achieve the goal by manipulation of the bicyclo[4.3.0]non-3-ene system. We herein detail synthesis of the mucosin diastereomer 1*, demonstrating aspects of the chosen route with regard to stereochemical control (Figure 2). From the point of potential biological activity, the putative structure of mucosin shares some apparent structural similarities with bicyclic prostaglandins. Thus, providing that sufficient amounts could be made available, material could be screened in similar assays. Currently, it is the bearer of unknown properties.

## 2. Results and Discussion

In 2012, Whitby and co-workers reported that they had completed the first total synthesis of antipodal mucosin (*ent*-**1**) [37]. Using zirconium induced co-cyclisation as the pivotal feature, the preliminary experimental work led them to conclude that thermodynamic control would favour the relative stereochemistry assigned by Casapullo et al. [35]. Applied to the actual sequence, elaboration of the key zirconacycle afforded a ~3:1 mixture of diastereomers [37]. While it was conjectured that the major component could be processed to *ent*-**1**, the minor component would in turn yield *ent*-1*. However, although Whitby and co-workers provided data that aligned with the natural product, the authors of the present paper demonstrated irrefutably, that mucosin is not represented by the relative topological connectivity featured in structure **1** [36]. This therefore raised the question as to which diastereomer had been taken on by Whitby and co-workers, and whether mucosin in fact corresponds to structure 1*. In order to resolve this pressing issue, we designed a strategy to access structure 1*.

**Scheme 1:**
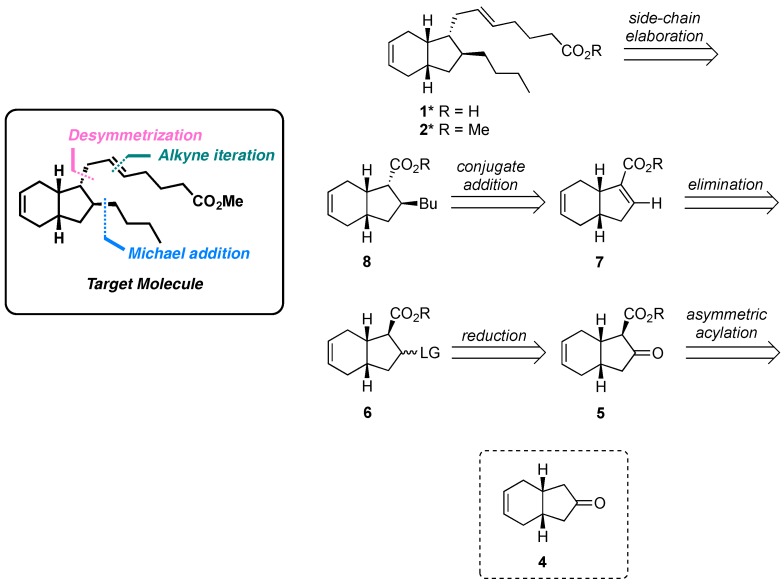
Key strategic points towards synthesis of *exo*-mucosin (1*).

**Figure 3:**
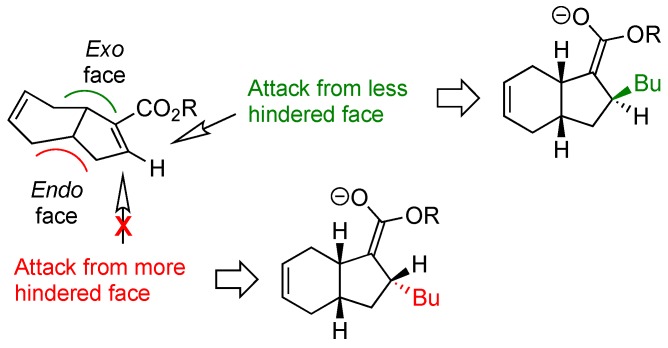
Projected diastereofacial bias in the key conjugate addition.

A central feature in our divergent strategy (Scheme 1) was to take advantage of the efficient desymmetrization of *meso*-ketone **4** [36]. (Supplementary Materials pp. S3, S4 provides the synthetic sequence to obtain *meso*-ketone **4**) After a chiral foothold had been established, it would then be a matter of introducing a functional pattern amenable for subsequent stereoiteration. Ideally, in order to uncover the topologically deviant point(s), the prerequisite *cis*-fused keto ester **5** should also be interconvertible with the *trans*-fused system if need be. However, we first chose to examine the configuration at the appended positions. Thus, along these lines and having previously established a diastereochemical bias, addition of some suitable nucleophile to conjugate ester **7** was judged to follow the precognised trend. By completing the sequence, a new compound 1* with the topology inverted at C8 and C16 would result. Aptly, this could then be named *exo*-mucosin 1*, since the bulky group added during the stereodifferentiating step was projected to occupy the *exo* face of the bicycle (Figure 3). Once *exo*-mucosin 1* had been made, the physical data recorded could be compared against those published for the natural product.

**Scheme 2:**
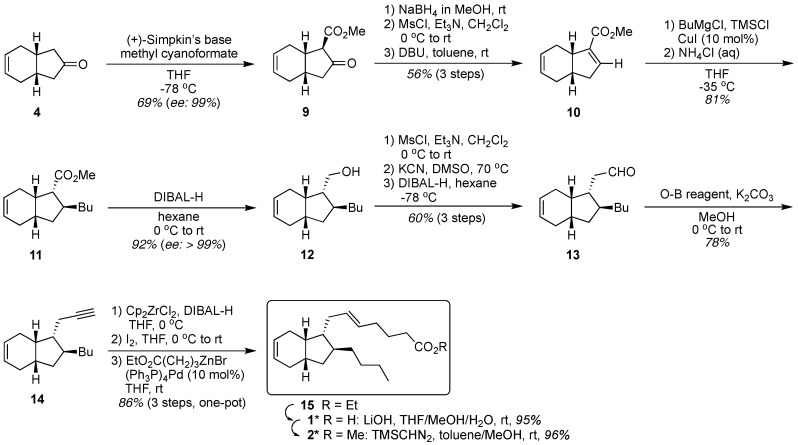
The total synthesis of *exo*-mucosin (1*) and its methyl ester 2*.

By the developed protocol, our synthesis commenced with desymmetrization of *meso*-ketone **4** [36], using Mander’s reagent in combination with the lithium amide of (+)-*bis*[(*R*)-phenyethyl]amine (Scheme 2). This chiral amide is sometimes also referred to as Simpkins’ base [38,39,40]. Then, with asymmetric keto ester **9** in hand, conjugated ester **10** was prepared by a three-step procedure, involving sequential manipulation of the keto moiety. Accordingly, the ketone in **9** was reduced, whereupon the corresponding alcohol was turned into a mesylate. Finally, the intermediate mesylate was subjected to base-induced elimination, whereby the Michael acceptor motif was produced.

Having carried out the delineated transformation, the key stereoiterative concept could be tested in the elaboration of conjugated ester **10**. While addition to the less hindered *exo*-face seemed inevitable, the resulting stereochemistry at the ester-appended chiral centre was somewhat uncertain a priori. Simplistically, depending on whether the supervening ester enolate is intercepted by H^+^ at the equatorial or axial position of C8, the protonated species will correspond to the kinetic and the thermodynamic product, respectively. Reflecting the ambivalent stereochemical nature of the C8-carbanion, and based on our previous experience [36], conjugate addition to **10** could consequently lead to a mixture of epimers.

**Scheme 3:**
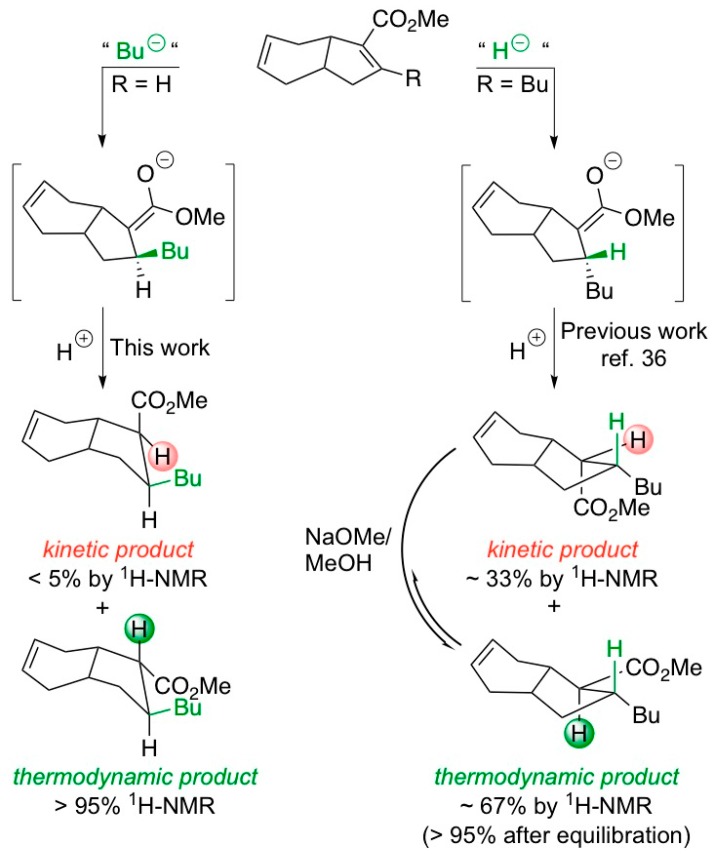
Observed divergent diastereoselectivity in the conjugate addition to Michael acceptor **10**.

**Figure 4:**
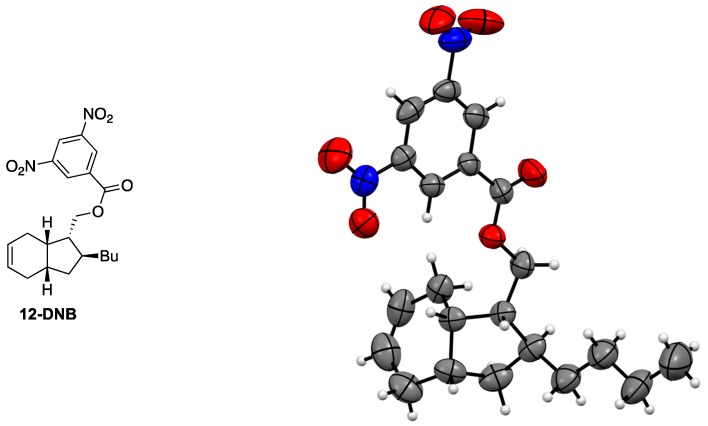
Single crystal X-ray structure obtained from the 3,5-dinitrobenzoate of the advanced intermediate **12** at 298 K. The structure is deposited at Cambridge Crystallographic Data Centre as CCDC 1535632.

In reality, with Cu(I)-catalysed conjugate addition, using BuMgCl as nucleophile in the presence of TMSCl, the reaction gave ester **11** as the sole compound (Scheme 3). Presumably, the Lewis acid takes on dual roles: Not only does TMSCl lower the LUMO of the Michael acceptor, but also stabilizes the ester enolate [41,42,43,44,45,46]. Hence, ester **11** ought to be the conjectured thermodynamic product. Subsequent reduction provided the corresponding carbinol **12**, which could also be readily derivatized for the purpose of X-ray analysis. By obtaining suitable crystals of the dinitrobenzoate **12-DNB**, the relative configuration of the four contiguous stereocentres could be established (Figure 4). This also confirmed the *exo*-facial and thermodynamic preference in the reaction of **10**, using the specified conditions. (Supplementary Figure S-74 provides a side perspective of the single crystal X-ray structure **12-DNB**).

With the intended topological pattern confirmed, carbinol **12** was taken through a course of four steps to install an alkyne handle by the Ohira-Bestmann protocol [47,48,49,50]. For the last step, it may be noted that Taber et al. have provided an interesting alternative to the rather pricy reagent [50]. Although ^1^H-NMR of the natural product clearly indicates the presence of an *E*-alkene [35], the *en route* aldehyde **13** could also serve as a relay point for *Z*-selective olefination. However, with the cited observation in mind, alkyne **14** was transformed accordingly to provide the featured *E*-configured alkenyl ester motif. This was achieved by performing three consecutive reactions in one-pot. Thus, by means of stereospecific hydrometallation [51,52,53,54,55] and halodemetallation [56], alkyne **14** rendered the corresponding *E*-vinyl halide as substrate for Pd-catalysed cross-coupling with a commercial zinc reagent [57,58]. The target molecule, *exo*-mucosin 1*, was then obtained after hydrolysis of ester **15**. Finally, re-esterification gave methyl ester 2*, to be compared with the data published by Casapullo et al. [35].

The *cis*-fused bicyclo[4.3.0]non-3-ene system is not often encountered in nature. Adhering to the supposition that arachidonic acid (**3**) is the biogenetic origin of mucosin [59,60,61], the geometry proposed for the core structure invokes a formal disrotatory ring-closure [62]. At a more profound level, the machinery leading to the natural product may traverse any number of pericyclic pathways [36]. Of particular interest, though, is the ongoing discussion regarding whether or not enzyme-catalysed Diels-Alder reactions are implicated in biological systems [63]. The preceding biosynthetic transformation of **3**, into a suitable conjugated precursor for cycloaddition, is known to take place in several marine species [59,60,61,64,65,66,67,68,69,70,71,72,73,74,75,76,77]. However, in all the cases where a Diels-Alderase could be claimed to provide the transformative impetus [78,79,80,81,82,83,84,85], the authors of this paper have found no example of *cis*-fusion.

Cycloaddition via a non-enzymatic pathway is also possible. Thus, Gerwick has proposed allylic carbocations as conceptual intermediates in the biogenesis of marine carbocyclic oxylipins, such as prostaglandin A2 (PGA_2_) [86,87,88]. In this sense, arachidonic acid (**3**) provides a link between mucosin and the prostanoid scaffold, pointing towards a possible mechanism. Yet, for the majority of examples found, the annulation produces a *trans*-1,2-disubstituted cyclopentane ring [89,90,91,92,93,94].

Although being an uncommon structural feature, it would be premature to conclude that the *cis*-fused bicyclo[4.3.0]non-3-ene system was incongruous. Nevertheless, when recordings were made on methyl ester 2*, the data did not match those reported for the compound isolated from *Reniera mucosa*. This was most convincingly demonstrated by comparing the ^13^C-NMR spectra (Table 1): Out of the 20 resonances that are observable for the carbon framework, excluding the methoxy group, 16 display deviating shifts (see also Supplementary Figure S-38). Furthermore, the optical rotation of 2* did not only differ in magnitude, but also in sign: While the naturally occurring material and its purported structure **2** have values of ${\left[\alpha \right]}_{D}^{26}=-35.5{}^{\circ}$ and −9.8° (c=0.8, hexane), respectively [35,36] the diastereomer 2* had an ${\left[\alpha \right]}_{D}^{26}=+64.0{}^{\circ}$ (c=0.8, hexane). Whitby and co-workers have reported ${\left[\alpha \right]}_{D}^{26}=+38.2{}^{\circ}$ (c=0.8, hexane) for the material obtained via zirconium induced co-cyclisation [37].

By achieving a rational synthesis of *exo*-mucosin 2*, the target selection has been narrowed down. Yet, in terms of the *cis*-fused bicycle, there are permutants still unaccounted for. However, given the obvious sterical encumbrance of the two remaining *syn*-diastereomers, they seemed unlikely candidates considering the biogenesis of marine carbocyclic oxylipins [86,87,88]. Additionally, it is worth noticing that the *anti*-relationship of the appended groups seems to rest on a sounder foundation: Diagnostic correlations between the C7-methylene group and the C16-proton have been observed by NOESY and ROESY [35]. Furthermore, DFT calculations by Whitby and co-workers on the relative stability of zirconacycles [37], indicate that the *anti*-geometries are favoured over the *syn*-geometries. Hence, based on our synthetic endeavours, it was inferred that the natural product named mucosin has a *trans*-fused bicyclo[4.3.0]non-3-ene ring system and the featured substituents are *anti*-related.

**Scheme 4:**
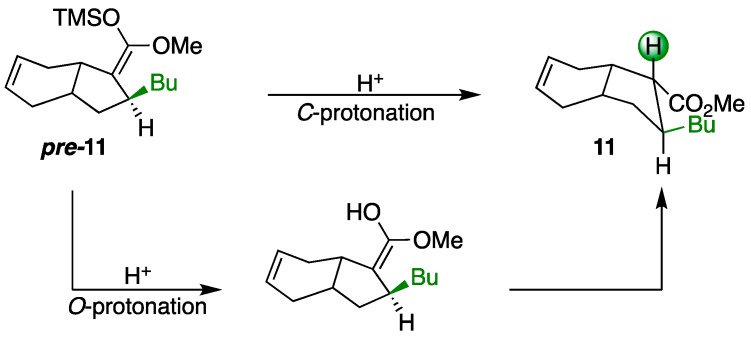
Silyl ketene acetal as the source of face-selective protonation.

Albeit that the outlined synthesis did not yield the ultimate target, the sequence provided an answer to a central question: namely, the question about the geometry of the fused bicycle. Moreover, taken together with what we have detailed before [36], the current findings have demonstrated a fascinating chemical aspect of the *cis*-fused bicyclo[4.3.0]non-3-ene scaffold, that unfolds when a Michael acceptor motif is incorporated. Thus, swapping the functional group at the β-position of the Michael acceptor with the functional group of the Michael donor, a complete inversion of diastereoselectivity was observed. The transformation proved to be doubly orthogonal, as even the stereochemistry at the α-position was inverted in the process. Bearing in mind the chosen protocol, it must be assumed that the favoured epimer is not obtained via spontaneous equilibration of the incipient ester enolate anion. Rather, the reactive constellation between BuMgCl and TMSCl intercepts the Michael adduct as a silyl ketene acetal; it is therefore in the succeeding protonation of the trapped ester enolate anion that the observed epimeric configuration is established (Scheme 4). The stereoselective protonation of enolates is a concept, which has received a great deal of attention and in its purest form constitutes a biomimetic approach to establish α-chirality [95,96,97,98]. In our example, the pre-existing topology works in consonance to dictate which face is being protonated.

In summary, the conjugate system shown in Scheme 3 (vide supra) displays a remarkable diastereotopic preference, enabling excellent control over the reactive manifold.

## 3. Experimental Section

### 3.1. General Information

All commercially available reagents and solvents were used in the form they were supplied without any further purification. (+)-Bis[(R)-1-phenylethyl]amine hydrochloride (optical purity ≥ 99% ee by GLC) was purchased from Sigma-Aldrich (St. Louis, MO, USA). The stated yields are based on isolated material. The melting points are uncorrected. Thin layer chromatography was performed on silica gel 60 F_254_ aluminum-backed plates fabricated by Merck (Kenilworth, NJ, USA). Flash column chromatography was performed on silica gel 60 (40–63 µm) fabricated by Merck. NMR spectra were recorded on a Bruker Ascend^TM^ 400 (Bruker, Billerica, MA, USA) at 400 MHz for ^1^H-NMR and at 100 MHz for ^13^C-NMR. Coupling constants (*J*) are reported in hertz (Hz) and chemical shifts are reported in parts per million (δ) relative to the central residual protium solvent resonance in ^1^H-NMR (CDCl_3_ = δ 7.27) and the central carbon solvent resonance in ^13^C-NMR (CDCl_3_ = δ 77.00 ppm). The following abbreviation, appt, has been used to designate an apparent triplet. Mass spectra were recorded at 70 eV on Waters Prospec Q spectrometer (Waters Corporation, Milford, MA, USA) using EI as the method of ionization. IR spectra (4000–600 cm^−1^) were recorded on a Perkin-Elmer Spectrum BX series FT-IR spectrophotometer (Waltham, MA, USA) using a reflectance cell (HATR). Optical rotations were measured using a 1 mL cell with a 1.0 dm path length on a Perkin Elmer 341 polarimeter using the stated solvents. Determination of enantiomeric excess was performed by GLC on an Agilent Technologies 7820A GC instrument (Agilent Technologies, Santa Clara, CA, USA) with split (1:30) injection, FID detector and equipped with a chiral stationary phase (Agilent J&W GC columns, CP-Chirasil-DEX CB, 25 m, 0.25 mm, 0.25 μm) applying the conditions stated. X-ray crystallography was performed on a Bruker D8 Venture diffractometer with InCoatec ImuS Microfocus radiation source and Photon 100 CMOS detector. Data collection with Apex2 [99], data integration and cell refinement with SAINT,1 absorption correction by SADABS [99], structure solution with SHELXT [100], structure refinement with SHELXL [101]. Molecular graphics from Mercury [102].

### 3.2. Synthesis of Keto Ester ***9***

(+)-Bis[(R)-1-phenylethyl]amine hydrochloride (2.5 g, 9.60 mmol, 1.58 equiv.) was added in one portion to dry THF (10 mL) at ambient temperature and stirred for 5 min. The stirring suspension was then cooled to −78 °C and BuLi (2.5M in hexane) (7.67 mL, 19.18 mmol, 3.16 equiv.) was added dropwise. The suspension changed colour from cloudy white to pale orange. After stirring at −78 °C for 15 min the suspension was warmed to ambient temperature whereby a transparent yellow solution was formed. This recooled to −78 °C and *meso*-(1*S*,6*R*)-bicyclo[4.3.0]non-3-ene-8-one **4** (0.826 g, 6.07 mmol, 1.0 equiv.) was added dropwise over 10 min in dry THF (10 mL). This mixture was then stirred for 45 min whereby a purple colour evolved. Methyl cyanoformate (0.96 mL, 12.14 mmol, 2.0 equiv.) was then added dropwise over 5 min. and the mixture immediately turned bright yellow in colour. This mixture was left stirring for 2.5 h and then quenched by addition of H_2_O (2 mL) at −78 °C. The mixture was then warmed to r.t. and extracted with EtOAc (2 × 50 mL). The resulting organic layer was then washed with H_2_O (2 × 100 mL), 0.5 M HCl (1 × 100 mL) and brine (1 × 100 mL). The organic layer was then dried over MgSO_4_, filtered and concentrated in vacuo. The resulting crude keto ester was purified by column chromatography (hexane/EtOAc 5:1) to form a colourless oil. This oil was then recrystallised from hexane at 0 °C, filtered and air dried to obtain the compound **9** as white crystals. All spectroscopic and physical data were in full agreement with those reported in the literature [103]. Yield: 0.812 g (69%); ${\left[\alpha \right]}_{D}^{26}=-161{}^{\circ}$ (c=0.1, CHCl_3_); ^1^H-NMR (400 MHz, CDCl_3_): δ 5.73–5.66 (m, 2H), 3.76 (s, 3H), 3.04 (d, *J* = 11.1 Hz, 1H), 2.88–2.83 (m, 1H), 2.52–2.38 (m, 3H), 2.33–2.21 (m, 2H), 2.04 (dd, *J* = 1.9, 18.2 Hz, 1H), 1.67–1.61 (m, 1H); ^13^C-NMR (100 MHz, CDCl_3_): δ 211.6, 169.7, 124.9, 123.9, 57.7, 52.4, 46.6, 37.3, 29.7, 26.8, 25.3; IR (neat, cm^−1^) 3034 (w), 2945 (m), 2908 (m), 2837 (w), 1751 (s), 1718 (s) 1656 (w) 1433 (s) 1404 (m); HRMS (EI+): Exact mass calculated for C_11_H_14_O_3_ [M]^+^: 194.0943 found 194.0933; m.p.: 59–61 °C; TLC (hexane/EtOAc 4:1, KMnO_4_ stain): R_f_ = 0.42.

### 3.3. Synthesis of Michael Acceptor ***10***

#### 3.3.1. Synthesis of α-Hydroxy Ester ***pre*-10a**

To stirring solution of **9** (1.40 g, 7.21 mmol, 1.0 equiv.) in MeOH (50 mL) at 0 °C was added NaBH_4_ (0,410 g, 10.8 mmol, 1.5 equiv.). The reaction was monitored by TLC and was deemed complete after 1 h. Then, dilute aq. HCl (10 mL, 1 M) was added drowise at 0 °C. The quenched reaction was concentrated in vacuo to afford a crude mixture. This was poured over Et_2_O (50 mL), whereupon water (50 mL) was added. The organic layer was separated and the aqueous layer was extracted with Et_2_O (2 × 50 mL). The organic layers were combined, washed with brine (1 × 100 mL), dried over MgSO_4_, filtered and concentrated in vacuo to afford a colourless, oily, residue. The crude was purified by column chromatography on silica (hexane/EtOAc 7:3) to afford the C8-epimeric compound ***pre*-10a** as colourless oil. Yield: 1.07 g (76%); ^1^H-NMR (400 MHz, CDCl_3_): δ 5.81–5.71 (m, 2H), 4.57–4.39 (m, 1H), 3.73 (s, 3H), 2.65–2.50 (m, 1H), 2.38–2.10 (m, 6H), 2.10–1.84 (m, 2H), 1.52–1.42 (m, 1H); ^13^C-NMR (100 MHz, CDCl_3_): δ 175.5 (major), 174.8 (minor), 127.5 (minor), 126.7 (minor), 126.6 (major), 125.6 (major), 75.3 (major), 73.0 (minor), 57.3 (major), 53.9 (minor), 51.8 (major), 51.7 (minor), 42.1 (minor), 40.9 (major), 39.0 (major), 38.0 (minor), 34.0 (minor), 33.6 (major), 27.9 (minor), 27.7 (major), 26.3 (major), 26.2 (minor); IR (neat, cm^−1^) 3439 (br), 3026 (w), 2914 (w), 2841 (w), 1712 (s), 1438 (m); HRMS (EI+): Exact mass calculated for C_11_H_16_O_3_ [M]^+^: 196.1099, found 196.1087; TLC (hexane/EtOAc 4:1, KMnO_4_ stain): R_f_ = 0.30.

#### 3.3.2. Synthesis of Mesylate ***pre*-10b**

To a stirring solution of C8-epimer ***pre***-**10a** (1.07 g, 5.45 mmol, 1.0 equiv.) in dry CH_2_Cl_2_ (50 mL) at ambient temperature was added Et_3_N (1.14 mL, 8.18 mmol, 1.5 equiv.) in a dropwise manner. The resulting mixture was left stirring for 5 min. and then cooled to 0 °C. Subsequently, methanesulfonyl chloride (0.51 mL, 6.59 mmol, 1.2 equiv.) was added in a dropwise manner and the reaction mixture was left stirring for 10 min. with continued cooling. Then, the cooling was discontinued and the reaction mixture was allowed to attain ambient temperature overnight. At this point the reaction mixture had turned from colourless to yellow. Brine (20 mL) was added in a dropwise manner and the volatiles were removed in vacuo. The resulting yellow liquid was poured over EtOAc (50 mL) and satd. aq. NaHCO_3_ (50 mL) was added. The organic layer was separated and the aqueous layer was extracted with EtOAc (2 × 50 mL). The organic layers were combined and washed with brine (1 × 100 mL), dried over MgSO_4_, filtered and concentrated in vacuo to afford a yellow, oily, residue. The crude was purified by column chromatography on silica (hexane/EtOAc 4:1) to afford the C8-epimeric compound ***pre*-10b** as a yellow oil. Yield: 1.16 g (77%); ^1^H-NMR (400 MHz, CDCl_3_) δ 5.81–5.77 (m, 0.3H, minor), (m, 1.7H, major), 5.37–5.31 (m, 1H), 3.74 (s, 2.6H, major), 3.72 (s, 0.4H, minor), 3.01 (s, 2.6H, major), 2.96 (s, 0.4H, minor), 2.88–2.82 (m, 1H), 2.4.06 (m, 6H), 2.02–1.87 (m, 1H), 1.86–1.78 (m, 1H); ^13^C-NMR (100 MHz, CDCl_3_) δ 173.9, 127.3 (minor), 126.8 (minor), 125.6 (major), 124.4 (major), 83.9 (major), 83.8 (minor), 54.6 (major), 53.6 (minor), 52.2 (major), 51.9 (minor), 40.9 (minor), 39.8 (major), 39.1 (major), 38.3 (minor), 37.9 (major), 36.4 (minor), 33.9 (major), 33.5 (minor), 27.6 (minor), 26.6 (major), 26.0 (minor), 25.4 (major); IR (neat, cm^−1^) 3031 (w), 2942 (w), 2847 (w), 1734 (s), 1438 (w), 1354 (s); HRMS (EI+): Exact mass calculated for C_12_H_18_O_5_S [M]^+^: 274.0875, found 274.0865; TLC (hexane/EtOAc 4:1, KMnO_4_ stain): R_f_ = 0.45.

#### 3.3.3. Synthesis of Michael Acceptor **10**

To a stirring solution of C8-epimer ***pre*-10b** (1.40 g, 5.10 mmol, 1.0 equiv.) in dry toluene (30 mL) at ambient temperature was added DBU (1.73 mL, 11.6 mmol, 2.3 equiv.) in a dropwise manner over 5 min. The reaction mixture was stirred overnight at the stated conditions. Having deemed the reaction complete by TLC, water (10 mL) and dilute aq. HCl (10 mL, 0.5 M) was added. The resulting mixture was poured over Et_2_O (20 mL) and the organic layer was separated. The aqueous layer was extracted with Et_2_O (2 × 30 mL). The organic layers were combined, washed in succession with water (1 × 50 mL) and brine (1 × 50 mL), dried over MgSO_4_, filtered and concentrated in vacuo to obtain a oily residue. The crude was purified by column chromatography on silica (hexane/EtOAc 9:1) to afford the compound **10** as colourless oil. Yield: 0.866 g, (95%); ${\left[\alpha \right]}_{D}^{26}=+180{}^{\circ}$ (c=0.8, CHCl_3_); ^1^H-NMR (400 MHz, CDCl_3_) δ 6.80–6.69 (m, 1H), 5.95–5.84 (m, 1H), 5.84–5.74 (m, 1H), 3.74 (s, 3H), 3.04–3.92 (m, 1H), 2.67–2.51 (m, 2H), 2.50–2.36 (m, 1H), 2.34–2.12 (m, 2H), 2.00–1.82 (m, 2H); ^13^C-NMR (100 MHz, CDCl_3_) δ 165.6, 143.5, 140.8, 128.3, 127.0, 51.2, 41.2, 39.4, 36.0, 27.8, 26.6; IR (neat, cm^−1^) 3031 (w), 2931 (w), 2841 (w), 1712 (s), 1628 (w), 1438 (m); HRMS (EI+): Exact mass calculated for C_11_H_14_O_2_ [M]^+^: 178.0994, found 178.1000; TLC (hexane/EtOAc 4:1, KMnO_4_ stain): R_f_ = 0.60.

### 3.4. Synthesis of Michael Adduct ***11***

To a solution of Michael acceptor **10** (0.421 g, 2.36 mmol, 1.0 equiv.) in dry THF (20 mL) at −35 °C was added in succession CuI (0.045 g, 0.24 mmol, 0.1 equiv.) and TMSCl (0.641 g, 0.75 mL, 5.90 mmol, 2.5 equiv.). The resulting slightly heterogenous mixture caused by suspended CuI was stirred for 5 min, whereupon BuMgCl (2.0 M in THF) (2.36 mL, 4.72 mmol, 2.0 equiv.) was added in a dropwise manner during the course of 2 h, maintaining the temperature between at −35 °C. Initial colours cycled between clear and yellow, but gradually took a transient purple hue while reverting to clear. Upon completing the addition, the purple colour persisted (cloudy amethyst). At this point, TLC revealed that the starting material had been consumed. The reaction was treated with aq. satd. NH_4_Cl (5 mL) and diluted with Et_2_O/water (30 mL, 2:1). The phases were separated and the aq. phase was extracted with Et_2_O (3 × 20 mL). The combined org. phases were washed with brine (15 mL), dried over MgSO_4_, filtered and the solvent was evaporated *in vacuo*. The residue was purified by column chromatography on silica (hexane/EtOAc 90:10) to afford compound **11** as a colourless oil. Yield: 0.496 g (81%); ${\left[\alpha \right]}_{D}^{26}=+63{}^{\circ}$ (c=0.8, CHCl_3_); ^1^H-NMR (400 MHz, CDCl_3_) δ 5.66–5.56 (m, 2H), 3.67 (s, 3H), 2.58–2.46 (m, 2H), 2.36–2.16 (m, 3H), 1,95–1.68 (m, 4H), 1.45–1.35 (m, 2H), 1.35–1.15 (m, 5H), 0.87 (t, *J* = 7.0 Hz, 3H); ^13^C-NMR (100 MHz, CDCl_3_) δ 174.8, 124.7, 124.6, 56.0, 51.3, 38.9, 37.4, 36.8, 36.0, 35.3, 30.4, 26.5, 22.8, 22.6, 14.1; IR (neat, cm^−1^) 3020 (w), 2925 (m), 1734 (s); HRMS (EI+): Exact mass calculated for C_15_H_24_O_2_ [*M*]^+^: 236.1776, found 236.1763; TLC (hexanes/EtOAc 80:20, KMnO_4_ stain): R_f_ = 0.70.

### 3.5. Synthesis of Carbinol ***12***

Michael adduct 11 (0.496 g, 2.10 mmol, 1.0 equiv.) was dissolved in hexane (10 mL) at ambient temperature and stirred for 5 min. The solution was then cooled to 0 °C and DIBAL-H (1M in hexane) (4.2 mL, 4.20 mmol, 2.0 equiv.) was added dropwise over 5 min. The reaction was then left to warm to r.t. After 1 h the reaction was cooled back to 0 °C and quenched with sat. aq. NH_4_Cl (5 mL). The reaction mixture was allowed to warm to ambient temperature whereby a cloudy suspension occurred. This suspension was poured over sat. aq. NH_4_Cl (20 mL) and the organic layer separated. The aqueous layer was extracted with EtOAc (2 × 50 mL) and the organic layers combined, washed with H_2_O (1 × 100 mL), brine (1 × 100 mL), dried over MgSO_4_, filtered and concentrated in vacuo to give a crude cloudy oil. This was then purified by column chromatography on silica (hexane/EtOAc 95:5) to afford compound **12** as a colourless oil. Yield: 0.400 g, (92%); ${\left[\alpha \right]}_{D}^{26}=+104{}^{\circ}$ (c=0.8, CHCl_3_); ^1^H-NMR (400 MHz, CDCl_3_) 5.77–5.51 (m, 2H), 3.77–3.58 (m, 2H), 2.38–2.22 (m, 1H), 2.22–2.05 (m, 2H), 2.05–1.71 (m, 4H) 1.71–1.52 (m, 2H), 1.52–1.35 (2H) 1.35–1.14 (m, 6H), 0.88 (t, *J* = 7.1 Hz, 3H); ^13^C-NMR (100 MHz, CDCl_3_) δ 125.3, 124.9, 63.3, 53.7, 38.1, 36.8, 36.3, 35.5, 35.4, 30.8, 26.6, 22.9, 21.6, 14.1; IR (neat, cm^−1^) 3328 (br.), 3020 (w), 2925 (s); HRMS (EI+): Exact mass calculated for C_14_H_24_O [*M*]^+^: 208.1827, found 208.1832; TLC (hexane/EtOAc 4:1, KMnO_4_ stain): R_f_ = 0.40. The enantiomeric excess was determined by chiral GLC analysis (CP-Chirasil-DEX CB, using the following program: 60 °C (45 min)—1 degrees/min to 160 °C—160 °C (5 min)): *t_r_*(*e_1_*, major) = 65.08 min and *t_r_*(*e_2_*, minor) = 65.67 min; *ee*: > 99% [104].

### 3.6. Synthesis of 3,5-Dinitrobenzoate ***12-DNB***

To a stirring solution of carbinol **12** (0.129 g, 0.546 mmol, 1.0 equiv.) in dry DCM (20 mL) was added Et_3_N (0.23 mL, 1.64 mmol, 3.0 equiv.) dropwise. The solution was then cooled to 0 °C and 3,5-dinitrobenzoyl chloride (0.215 g, 0.933 mmol, 1.7 equiv.) was added in one portion. The reaction was slowly warmed to ambient temperature and monitored by TLC until completion. After 2 h, the reaction mixture was poured over H_2_O (10 mL) and the organic layer separated. The aqueous layer was then extracted with DCM (2 × 10 mL) and the organic layers combined. The organic layers were then washed with H_2_O (1 × 30 mL), brine (1 × 30 mL), dried with MgSO_4_, filtered and concentrated in vacuo to form a crude orange oil. This was purified by column chromatography on silica (hexane/EtOAc, 95:5) to afford the compound **12-DNB** as a white powder. Yield: 0.193 g (88%), ${\left[\alpha \right]}_{D}^{26}=+42{}^{\circ}$ (c=0.8, CHCl_3_); ^1^H-NMR (400 MHz, CDCl_3_) δ 9.23 (t, J=2.2 Hz, 1H), 9.14 (d, J=2.2 Hz, 2H), 5.70–5.61 (m, 2H), 4.49 (s, 1H), 4.47 (d, J=1.9 Hz, 1H), 2.37–2.10 (m, 4H), 2.02–1.88 (m, 2H), 1.88–1.68 (m, 3H), 1.58–1.43 (m, 2H), 1.40–1.23 (m, 5H), 0.89 (t, J=6.7 Hz, 3H); ^13^C-NMR (100 MHz, CDCl_3_) δ 162.5, 148.7, 134.1, 129.3, 125.4, 124.3, 122.3, 67.7, 49.5, 38.6, 36.9, 36.7, 35.5, 35.4, 30.8, 26.5, 22.9, 21.8, 14.1; IR (neat, cm^−1^) 3098 (w), 3020 (w), 2931 (m), 1723 (s), 1538 (s); HRMS (EI+): Exact mass calculated for C_21_H_26_N_2_O_6_ [*M*]^+^: 402.1791, found 402.1797; m.p.: 117 °C; TLC (hexane/EtOAc 4:1, KMnO_4_ stain): R_f_ = 0.75. [105]

### 3.7. Synthesis of Aldehyde ***13***

#### 3.7.1. Synthesis of Mesylate ***pre*-13a**

To a stirring solution of carbinol **12** (0.400 g, 1.92 mmol, 1.0 equiv.) in dry CH_2_Cl_2_ (5 mL) at ambient temperature, was added Et_3_N (0.54 mL, 3.84 mmol, 2.0 equiv.) dropwise. This solution was left stirring for 5 min then cooled to 0 °C. Then methanesulfonyl chloride (0.45 mL, 5.76 mmol, 3.0 equiv.) was added dropwise and the reaction was left at 0 °C for 10 min then warmed to ambient temperature and left overnight. The reaction mixture turned colourless to yellow. Then, brine (10 mL) was added dropwise and the volatiles concentrated in vacuo to afford a yellow liquid. This was poured over EtOAc (50 mL) and sat. aq. NaHCO_3_ (50 mL) was added. The organic layer was separated and the aqueous layer extracted with EtOAc (2 × 50 mL). The organic layers were combined and washed with brine (1 × 50 mL), dried over MgSO_4_, filtered and concentrated in vacuo to afford a crude yellow oil. This was then purified by column chromatography on silica (hexane/EtOAc 95:5) to afford the compound ***pre*-13a** as a colourless oil. Yield: 0.497 g, (90%); ${\left[\alpha \right]}_{D}^{26}=+79{}^{\circ}$ (c=0.8, CHCl_3_); ^1^H-NMR (400 MHz, CDCl_3_) δ 5.60–5.50 (m, 2H), 4.21–4.12 (m, 2H), 2.94 (s, 3H), 2.25–2.11 (m, 1H), 2.11–1.99 (m, 2H), 1.98–1.83 (m, 2H), 1.83–1.76 (m, 1H), 1.71–1.55 (m, 3H), 1.40–1.30 (m, 2H), 1.28–1.10 (m, 5H), 0.82 (t, *J* = 7.0 Hz, 3H); ^13^C-NMR (100 MHz, CDCl_3_) δ 125.3, 124.4, 70.3, 50.0, 38.1, 37.4, 36.6, 36.4, 35.4, 35.3, 30.7, 26.4, 22.8, 21.4, 14.1; IR (neat, cm^−1^) 3020 (w), 2925 (m), 1354 (s); HRMS (EI+): Exact mass calculated for C_15_H_26_O_3_S_2_ [*M*]^+^: 286.1603, found 286.1627; TLC (hexane/EtOAc 4:1, KMnO_4_ stain): R_f_ = 0.50.

#### 3.7.2. Synthesis of Nitrile ***pre*-13b**

To a stirring solution of mesylate *pre*-13a (0.497 g, 1.74 mmol, 1.0 equiv.) in dry DMSO (30 mL) was added solid KCN (0.675 g, 10.4 mmol, 6.0 equiv.) in one portion. The reaction mixture was then heated to 70 °C for 2 h. The reaction mixture changed from colourless to yellow. Then, the reaction was cooled to r.t. and H_2_O (5 mL) was added dropwise. The reaction mixture turned from yellow to colourless. This was then poured over EtOAc (20 mL) and the organic layer separated. The aqueous layer was extracted with EtOAc (2 × 20 mL) and the organic layers combined. They were then washed with brine (1 × 50 mL), dried over MgSO_4_, filtered and concentrated in vacuo to afford a crude brown oil. This was then purified by column chromatography on silica (hexane/EtOAc 98:2) to give the compound ***pre*-13b** as a colourless oil. Yield: 0.325, (86%); ${\left[\alpha \right]}_{D}^{26}=+111{}^{\circ}$ (c=0.8, CHCl_3_); ^1^H-NMR (400 MHz, CDCl_3_) δ 5.82–5.47 (m, 2H), 2.42–2.28 (m, 2H), 2.18–2.14 (m, 2H), 2.04–1.85 (m, 3H), 1.74–1.65 (m, 3H), 1.51–1.35 (m, 2H), 1.33–1.19 (m, 5H), 0.89 (t, *J* = 7.0 Hz, 3H) ^13^C-NMR (100 MHz, CDCl_3_) δ 125.4, 124.2, 119.6, 47.3, 41.4, 37.9, 35.8, 35.7, 35.1, 30.6, 26.5, 22.8, 21.5, 17.7, 14.1; IR (neat, cm^−1^) 3026 (w), 2919 (s), 2248 (w), 1465 (w) 1436 (w); HRMS (EI+): Exact mass calculated for C_15_H_23_N [*M*]^+^: 217.1830, found 217.1845; TLC (hexane/EtOAc 4:1, KMnO_4_ stain): R_f_ = 0.80.

#### 3.7.3. Synthesis of Aldehyde **13**

A stirring solution of nitrile ***pre*-13b** (0.322 g, 1.48 mmol, 1.0 equiv.) in hexane (10 mL) was cooled to −78 °C. Then DIBAL-H (1M in hexane) (2.20 mL, 2.22 mmol, 1.5 equiv.) was added dropwise over 5 min and the reaction left to stir for 20 min. Then sat. aq. Rochelle salt (5 mL) was added dropwise to the reaction mixture and then left to warm to ambient temperature. The resulting cloudy suspension was poured over EtOAc (20 mL) and sat. aq. Rochelle salt (20 mL). The organic layer was separated and the aqueous phase extracted with EtOAc (2 × 20 mL). The organic phases were combined and washed with brine (1 × 50 mL), dried over MgSO_4_, filtered and concentrated in vacuo to afford a crude cloudy oil. This was then purified by column chromatography on silica (hexane/EtOAc, 95:5) to afford the compound **13** as a colourless oil. Yield: 0.253 mg, (78%); ${\left[\alpha \right]}_{D}^{26}=+101{}^{\circ}$ (c=0.8, CHCl_3_); ^1^H-NMR (400 MHz, CDCl_3_) δ 9.79 (t, *J* = 2.3 Hz, 1H), 5.68–5.51 (m, 2H), 2.49–2.44 (m, 2H), 2.35–2.23 (m, 1H), 2.22–2.12 (m, 1H), 2.10–1.98 (m, 2H), 1.92–1.78 (m, 2H), 1.74–1.60 (m, 3H), 1.47–1.35 (m, 2H), 1.35–1.15 (m, 5H), 0.88 (t, *J* = 7.0 Hz, 3H); ^13^C-NMR (100 MHz, CDCl_3_) δ 202.9, 125.3, 124.7, 45.2, 44.7, 41.4, 37.5, 35.7, 35.6, 34.9, 30.8, 26.7, 22.9, 22.1, 14.1; IR (neat, cm^−1^) 3020 (w), 2919 (m), 2712 (w), 1723 (s); HRMS (EI+): Exact mass calculated for C_15_H_24_O [*M*]^+^: 220.1827, found 220.1824; TLC (hexane/EtOAc 4:1, KMnO_4_ stain): R_f_ = 0.80.

### 3.8. Synthesis of Alkyne ***14***

To a stirring solution of aldehyde **13** (0.253 g, 1.15 mmol, 1.0 equiv.) in dry MeOH (6 mL) at 0 °C was added solid K_2_CO_3_ (0.381 mg, 2.76 mmol, 2.4 equiv.) in one portion and Ohira-Bestmann reagent (10% *w*/*w* in MeCN) (3.9 mL, 3.32 g, 1.73 mmol, 1.5 equiv.). The suspension was then warmed to ambient temperature and left stirring 1 h. After analysis by TLC the mixture was treated with sat. aq. NaHCO_3_ (20 mL), and the resulting mixture poured over CH_2_Cl_2_ (20 mL). The organic phase was separated and the aqueous phase was washed with CH_2_Cl_2_ (3 × 10 mL). The organic phases were then combined, dried over Na_2_SO_4_, filtered and concentrated in vacuo to afford a crude oil. This was purified by column chromatography on silica (hexane/EtOAc, 95:5) to afford the compound **14** as a colourless oil. Yield: 0.193 g, (78%); ${\left[\alpha \right]}_{D}^{26}=+119{}^{\circ}$ (c=0.8, CHCl_3_); ^1^H-NMR (400 MHz, CDCl_3_) δ 5.72–5.58 (m, 2H), 2.35–2.24 (m, 2H), 2.18–2.09 (m, 3H), 2.07–1.98 (m, 1H), 1.91 (t, *J* = 2.7 Hz, 1H), 1.89–1.83 (m, 1H), 1.83–1.75 (m, 1H), 1.75–1.55 (m, 3H), 1.55–1.45 (m, 1H), 1.45–1.36 (m, 1H), 1.36–1.13 (m, 5H), 0.89 (t, *J* = 7.1 Hz, 3H); ^13^C-NMR (100 MHz, CDCl_3_) δ; 125.2, 125.0, 84.4, 67.9, 50.2, 41.2, 37.7, 36.1, 35.9, 35.2, 30.8, 26.8, 22.9, 21.4, 18.9, 14.1; IR (neat, cm^−1^) 3311 (m), 3020 (w), 2919 (s), 1432 (m); HRMS (EI+): Exact mass calculated for C_16_H_24_ [*M*]^+^: 216.1878, found 216.1867; TLC (hexane, KMnO_4_ stain and anisaldehyde dip): R_f_ = 0.25.

### 3.9. Synthesis of exo-Mucosin 1*

#### 3.9.1. Synthesis of Ethyl Ester **15**

To a stirring solution of Cp_2_ZrCl_2_ (0.400 g, 1.37 mmol, 2.0 equiv.) in dry THF (8 mL) at 0 °C was added DIBAL-H (1M in hexane) (1.37 mL, 1.37 mmol, 2.0 equiv.) via dropwise addition. The resulting homogenous mixture was then protected from light and stirred at 0 °C for 1 h after which time a colourless heterogeneous mixture formed. Then alkyne **14** (0.148 g, 0.684 mmol, 1.0 equiv.) dissolved in dry THF (4 mL) was added dropwise to the reaction mixture at 0 °C. After 1 h at 0 °C iodine (0.260 g, 1.02 mmol, 1.5 equiv.) was added in one portion to the homogeneous yellow reaction mixture. The reaction mixture was then warmed to ambient temperature and stirred for 1 h. To the preformed vinyl iodide was successively added 4-ethoxy-4-oxobutylzinc bromide solution (0.5M in THF) (2.7 mL, 1.37 mmol, 2.0 equiv.) dropwise and (Ph_3_P)_4_Pd (0.079 g, 0.068 mmol, 0.1 equiv.) in one portion. The resulting light brown mixture was stirred at ambient temperature for 1 h and monitored by TLC. Once the reaction had gone to completion 1 M HCl (10 mL) was added dropwise and the reaction poured over Et_2_O (15 mL). The aqueous phase was extracted with Et_2_O (3 × 50 mL) and the organic phases combined, dried over MgSO_4_, filtered and concentrated in vacuo to form a crude brown oily mixture. This oily mixture was purified by column chromatography on silica (hexane/EtOAc, 95:5) to afford the compound **15** as a colourless oil. Yield: 0.196 g, (86%); ${\left[\alpha \right]}_{D}^{26}=+67{}^{\circ}$ (*c* = 0.8, CHCl_3_); ^1^H-NMR (400 MHz, CDCl_3_) δ 5.69–5.56 (m, 2H), 5.48–5.33 (m, 2H), 4.12 (q, *J* = 7.1 Hz, 2 H), 2.31–2.21 (appt, *J* = 7.5 Hz, 3H), 2.16–1.82 (m, 8H), 1.75–1.50 (m, 6H), 1.48–1.41 (m, 1H), 1.39–1.22 (m, 9H), 0.88 (t, *J* = 7.1 Hz, 3H); ^13^C-NMR (100 MHz, CDCl_3_) δ 173.8, 131.2, 129.1, 125.3, 125.1, 60.2, 51.6, 41.3, 37.3, 36.2, 35.6, 35.5, 33.7, 33.0, 31.9 31.0, 26.9, 24.8, 23.0, 21.7, 14.2, 14.1; IR (neat, cm^−1^) 3020 (w), 2925 (m), 1734 (s); HRMS (EI+): Exact mass calculated for C_22_H_36_O_2_ [*M*]^+^: 332.2715, found 332.2709; TLC (hexane/EtOAc 95:5, KMnO_4_ stain): R_f_ = 0.65.

#### 3.9.2. Synthesis of *exo*-Mucosin 1*

To a stirring solution of ethyl ester **15** (0.177 g, 0.533 mmol, 1.0 equiv.) in THF/MeOH/H_2_O (2:2:1) (5 mL) at ambient temperature was added lithium hydroxide monohydrate (0.783 mg, 18.7 mmol, 35.0 equiv.) in one portion. The reaction mixture was left stirring and monitored by TLC. Left overnight, the reaction had gone to completion and was acidified to pH 2 by 1M HCl (5 mL). The reaction mixture was then poured over EtOAc (5 mL) and the aqueous phase extracted with EtOAc (3 × 5 mL). The organic phases were combined and washed with brine (1 × 20 mL), dried over MgSO_4_, filtered and concentrated in vacuo to provide a colourless oil. This was purified by column chromatography on silica (hexane/EtOAc, 3:2) to afford the compound 1* as a colourless oil. Yield: 0.154 g, (95%); ${\left[\alpha \right]}_{D}^{26}=+77{}^{\circ}$ (c=0.8, hexane); ^1^H-NMR (400 MHz, CDCl_3_) δ 11.63 (br, 1H), 5.67–5.56 (m, 2H), 5.50–5.34 (m, 2H), 2.34 (t, *J* = 7.5 Hz, 2H), 2.33–2.22 (m, 1H), 2.15–2.12 (m, 8H), 1.77–1.49 (m, 6H), 1.48–1.40 (m,1H), 1.40–1.08 (m, 6H), 0.88 (t, *J* = 6.7 Hz, 3H); ^13^C-NMR (100 MHz, CDCl_3_) δ 180.3, 131.4, 128.9, 125.3, 125.1, 51.6, 41.3, 37.3, 36.2, 35.6, 35.5, 33.4, 33.0, 31.8, 31.0, 26.9, 24.5, 23.0, 21.7, 14.2; IR (neat, cm^−1^) 3020 (w), 2925 (m), 1712 (s); HRMS (EI+): Exact mass calculated for C_20_H_32_O_2_ [*M*]^+^: 304.2402, found 304.2400; TLC (hexane/EtOAc 3:2, KMnO_4_ stain): R_f_ = 0.40.

#### 3.9.3. Synthesis of Methyl Ester 2*

To a stirring solution of *exo*-mucosin 1* (0.023 g, 0.076 mmol, 1.0 equiv.) in toluene/MeOH (3:2) (5 mL) at ambient temperature was added TMS diazomethane solution (2M in hexane) (0.06 mL, 0.113 mmol, 1.5 equiv.) dropwise over 2 min. The reaction mixture bubbled and turned transparent yellow. The reaction was monitored by TLC and after 1 h had gone to completion. The reaction mixture was then concentrated in vacuo and directly purified by column chromatography on silica (hexane/EtOAc, 95:5) to afford the compound 2* as a colourless oil. Yield: 23 mg, (96%); ${\left[\alpha \right]}_{D}^{26}=+64{}^{\circ}$ (*c* = 0.8, hexane); ^1^H-NMR (400 MHz, CDCl_3_) δ 5.69–5.56 (m, 2H), 5.49–5.32 (m, 2H), 3.66 (s, 3H), 2.29 (t, *J* = 7.5 Hz, 2H), 2.26–2.21 (m, 1H), 2.13–1.82 (m, 8H), 1.75–1.63 (m, 3H), 1.63–1.50 (m, 3H), 1.40–1.08 (m, 7H), 0.88 (t, *J* = 7.1 Hz, 3H); ^13^C-NMR (100 MHz, CDCl_3_) δ; 174.2, 131.2, 129.0, 125.3, 125.1, 51.6, 51.4, 41.3, 37.2, 36.2, 35.5, 35.4, 33.4, 33.0, 31.9, 31.0, 26.9, 24.7, 23.0, 21.7, 14.1; IR (neat, cm^−1^) 3020 (w), 2925 (s), 1745 (s); HRMS (EI+): Exact mass calculated for C_21_H_34_O_2_ [*M*]^+^: 318.2559, found 318.2552; TLC (hexane/EtOAc 95:5, KMnO_4_ stain): R_f_ = 0.65.

## 4. Conclusions

To recapitulate our findings, we have investigated the *anti*-diastereomer 2* of the proposed structure **2**. Through the execution of 13 discrete linear steps, the target molecule was obtained in an overall yield of 11% and in multi-milligram quantities. By incorporating a Michael acceptor motif, we have revealed an innate topological bias of the *cis*-fused bicyclo[4.3.0]non-3-ene system. Combined with our previously developed three step one-pot alkyne iteration, we have shown that *exo*-mucosin 1* is not identical to the natural product. In terms of the synthetic sequence detailed by Whitby and co-workers [37], our findings must necessarily have some mechanistic implications. Thus, we conclude that zirconium induced co-cyclisation did not deliver any of the two *anti*-diastereomers, **1** and 1* respectively, by the published procedure. Achieving a synthesis that establishes the true structure of mucosin will therefore also provide mechanistic insight into this matter. Based on the present work and what has previously been published [36,37], the fusion geometry of the hydrindane core embedded within mucosin is most likely *trans*.

## Figures and Tables

**Table 1:** Comparative ^13^C-NMR of methyl ester 2* (δ-values). ^†,‡^

Entry	Casapullo et al. [35]	Whitby et al. [37]	Previous Work [36]	This Work
1	174.2	174.2	174.2	174.2
2	130.0	130.3	130.4	***131.2***
3	129.8	129.8	129.9	***129.0***
4	127.0	127.3	***126.3***	***125.3***
5	127.0	127.1	***126.1***	***125.1***
6	52.1	52.2	***51.4***	***51.6***
7	51.4	51.4	51.0	51.4
8	47.1	47.2	***44.0***	***41.3***
9	42.1	42.3	***40.3***	***37.2***
10	39.9	40.1	***38.1***	***36.2***
11	36.7	37.0	***37.7***	***35.5***
12	36.5	36.74	***37.1***	***35.4***
13	36.4	36.68	***34.9***	***33.4***
14	33.2	33.4	33.4	33.0
15	32.0	32.4	31.9	31.9
16	31.7	31.9	***31.0***	***31.0***
17	31.5	31.6	***27.8***	***26.9***
18	30.7 ^§^	30.7	***27.7***	***24.7***
19	24.5	24.7	24.8	***23.0***
20	22.6	22.9	22.9	***21.7***
21	13.8	14.1	14.1	14.1

^†^ The italic bold numbers indicate deviating δ-values compared to Ref. [35]. ^‡^ Upon request, we have not been able to procure the original ^13^C-spectrum from the authors quoted in Ref. [35] for comparison. ^§^ According to communication rendered in the supporting information accompanying Ref. [37], the resonance at δ 30.7 had been omitted in Ref. [35], while an additional signal at δ 36.3 was observed. The data were subsequently revised and this fact is not touched upon in the main paper.

## Supplementary Materials

Electronic supplementary information is available online with full experimental procedures and characterisation data for all new compounds; crystal data and refinement details are included to give evidence of the relative stereochemistry of the late stage intermediate **12**.
