# Supplementary material for: Stereopermutation on the Putative Structure of the Marine Natural Product Mucosin
Source: Molecules. 2017 Oct 13;22(10):1720. doi: 10.3390/molecules22101720 (PMC6151738; doi:10.3390/molecules22101720)
Supplement: Supplementary file 1 [file molecules-22-01720-s001.pdf]

Stereopermutation on the Putative Structure of the Marine Natural Product Mucosin

Simen G. Antonsen,[1] Harrison C. Gallantree-Smith,[1] Carl H. Görbitz,[2] Trond V. Hansen,[1,3] Yngve H. Stenstrøm[1] and Jens M. J. Nolsøe[1]*

[1] *Faculty of Chemistry, Biotechnology and Food Science, Norwegian University of Life Sciences, PO box 5003, 1432 Ås, Norway*

[2] *Department of Chemistry, University of Oslo, PO box 1033, 0315 Oslo, Norway*

[3] *Department of Pharmaceutical Chemistry, University of Oslo, PO box 1068, 0316 Oslo, Norway*

**E-mail: jens.mj.nolsoe@nmbu.no*

Supporting Information

Table of Contents:

General Information........................ S1
Preparation of *meso*-ketone **4** (Scheme S1)........................ S3
NMR spectra of compounds in sequential order **S2**, **S3** and **4** (Figures S1-S6)........................ S5
MS spectra of compounds in sequential order **S2**, **S3** and **4** (Figures S7-S9)........................ S8
IR spectra of compounds in sequential order **S2**, **S3** and **4** (Figures S10-S12)........................ S10
Preparation of *exo*-mucosin **1*** and the methyl ester **2*** (Scheme S2)........................ S12
NMR spectra of compounds in sequential order **9-15**, **1*** and **2*** (Figures S13-S38)........................ S20
MS spectra of compounds in sequential order **9-15**, **1*** and **2*** (Figures S39-S51)........................ S33
IR spectra of compounds in sequential order **9-15**, **1*** and **2*** (Figures S52-S64)........................ S40
GLC chromatograms of late stage intermediate **12** (Figures S65-S68)........................ S47
Preparation of 3,5-dinitrobenzoate derivative of **12** (Scheme S3)........................ S51
NMR spectra of compound 3,5-dinitrobenzoate derivative of **12** (Figures S69 and S70)........................ S52
MS spectrum of compound 3,5-dinitrobenzoate derivative of **12** (Figure S71)........................ S53
IR spectrum of compound 3,5-dinitrobenzoate derivative of **12** (Figures S72)........................ S54
X-ray crystallography on 3,5-dinitrobenzoate derivative of **12** (Figures S73 and S74)........................ S55
References........................ S61

General Information

All commercially available reagents and solvents were used in the form they were supplied without any further purification. (+)-Bis[(*R*)-1-phenylethyl]amine hydrochloride (optical purity ≥ 99% *ee* by GLC) was purchased from Sigma-Aldrich. The stated yields are based on isolated material. The melting points are uncorrected. Thin layer chromatography was performed on silica gel 60 F_{254} aluminum-backed plates fabricated by Merck. Flash column chromatography was performed on silica gel 60 (40-63 µm) fabricated by Merck. NMR spectra were recorded on a Bruker Ascend™ 400 at 400 MHz for ^{1}H NMR and at 100 MHz for ^{13}C NMR. Coupling constants (*J*) are reported in hertz and chemical shifts are reported in parts per million (δ) relative to the central residual protium solvent resonance in ^{1}H NMR ($CDCl_3$ = δ 7.27) and the central carbon solvent resonance in ^{13}C NMR ($CDCl_3$ = δ 77.00 ppm). The following abbreviation, appt, has been used to designate an apparent triplet. Mass spectra were recorded at 70 eV on Waters Prospec Q spectrometer using EI as the method of ionization. IR spectra (4000–600 cm−1) were recorded on a Perkin-Elmer Spectrum BX series FT-IR spectrophotometer using a reflectance cell (HATR). Optical rotations were measured using a 1 mL cell with a 1.0 dm path length on a Perkin Elmer 341 polarimeter using the stated solvents. Determination of enantiomeric excess was performed by GLC on an Agilent Technologies 7820A GC instrument with split (1:30) injection, FID detector and equipped with a chiral

stationary phase (Agilent J&W GC columns, CP-Chirasil-DEX CB, 25 m, 0.25 mm, 0.25 µm) applying the conditions stated. X-ray crystallography was performed on a Bruker D8 Venture diffractometer with InCoatec ImuS Microfocus radiation source and Photon 100 CMOS detector. Data collection with Apex2 [1], data integration and cell refinement with SAINT,1 absorption correction by SADABS [1], structure solution with SHELXT [2], structure refinement with SHELXL [3]. Molecular graphics from Mercury [4].

Preparation of *meso*-ketone (4):

Step 1: *Step 2:* *Step 3:*

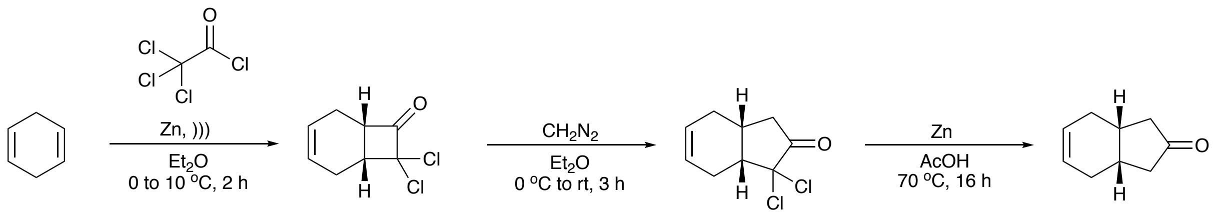

S1 **S2** **S3** **4**

Scheme S-1 Synthetic route to *meso*-ketone **4**.

***rac*-(1*R*,6*S*)-8,8-Dichlorobicyclo[4.2.0]oct-3-en-7-one (S2).[5]**

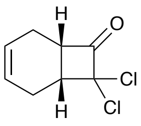

S2

1,4-Cyclohexadiene (5 g, 62.5 mmol, 1.0 equiv.) was added to a suspension of zinc powder (8.2 g, 125 mmol, 2.0 equiv.) in dry Et_2O (100 mL) and sonicated at 0 °C for 15 min. Then trichloroacetylchloride (22.8 g, 125 mmol, 2.0 equiv.) in dry Et_2O (100 mL) was added dropwise over 2 h while the reaction mixture was still sonicating. After completed addition the reaction mixture was sonicated for a further 2 h at 0-10 °C. The colour changed from colourless to dark yellow. The sonication was then stopped and the reaction mixture filtered and concentrated *in vacuo*. The resulting orange slurry was diluted in Et_2O (400 mL) and washed with H_2O (2 x 400 mL) and sat. aq. $NaHCO_3$ (1 x 400 mL). The organic layer was dried ($MgSO_4$), filtered and concentrated *in vacuo*. The resulting crude dark yellow oil was purified by column chromatography on silica (hexane/EtOAc 99:1) to afford the title compound as a colourless oil. All spectroscopic and physical data were in full agreement with those reported in the literature.[5] Yield: 8.67 g (73%); ^{1}H NMR (400 MHz, $CDCl_3$) δ 5.90-5.81 (m, 2H), 4.07-4.01 (m, 1H), 3.32 (ddt, *J* = 2.0, 7.9, 10.4 Hz, 1H), 2.63-2.50 (m, 2H), 2.39-2.32 (m, 1H), 2.17-2.10 (m, 1H); ^{13}C NMR (100 MHz, $CDCl_3$) δ 198.3, 127.3, 126.3, 88.5, 53.7, 45.2, 23.1, 21.; IR (neat, cm^{-1}) 3041 (w), 2939 (w), 2895 (w), 2841 (w) 1799 (s), 1644 (w), 1433 (m); HRMS (EI+): Exact mass calculated for $C_8H_8OCl_2$ $[M]^+$: 189.9952, found 189.9953; TLC (hexane/EtOAc 4:1, $KMnO_4$ stain): R_f = 0.65.

***rac*-(1*R*,6*R*)-7,7-Dichlorobicyclo[4.3.0]non-3-en-8-one (S3).**

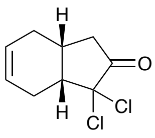

S3

To a stirring solution of *rac*-(1*R*,6*S*)-8,8-dichlorobicyclo[4.2.0]oct-3-en-7-one **S3** (2 g, 10.47 mmol, 1.0 equiv.) in dry Et_2O (50 mL), at 0 °C, was added diazomethane (2.47g, 104.7 mmol, 10.0 equiv.) in dry Et_2O (50 mL) dropwise over 15 min. The reaction mixture bubbled and turned a deep golden yellow colour. After 30 min the reaction was warmed to room temperature and left to stir for 2 h. The reaction was then quenched with glacial AcOH (5 mL) dropwise until there was no more gas evolution and the colour changed from golden yellow to almost colourless. The resulting mixture was then washed with H_2O (2 x 300 mL), sat. aq. $NaHCO_3$ (1 x 300 mL), brine (1 x 300 mL), dried with $MgSO_4$, filtered and concentrated *in vacuo*. The resulting dark yellow oil was purified by column chromatography on silica (hexane/EtOAc 9:1) to afford the title compound as a colourless oil. Yield: 1.6 g (75%); ^{1}H NMR (400 MHz, $CDCl_3$) δ; 5.64-5.57 (m, 2H), 2.86-2.80 (m, 1H), 2.74-2.69 (m, 1H), 2.54 (dd, *J* = 7.5, 19.2 Hz, 1H), 2.38-2.29 (m, 2H), 2.07-2.00 (m, 2H), 1.72-1.64 (m, 1H); ^{13}C NMR (100 MHz, $CDCl_3$) δ

201.7, 123.9, 123.1, 89.5, 46.7, 36.6, 28.1, 25.8, 23.5; IR (neat, cm^{-1}) 3033 (m), 2916 (m), 2842 (m), 1764 (s) 1662 (w) 1434 (m), 1402 (m); HRMS (EI+): Exact mass calculated for $C_9H_{10}OCl_2$ $[M]^+$: 204.0109, found 204.0103; TLC (hexane/EtOAc 4:1, $KMnO_4$ stain): R_f = 0.60.

***meso*-(1*S*,6*R*)-Bicyclo[4.3.0]non-3-ene-8-one (4).[6]**

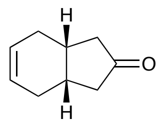

4

To a stirring suspension of zinc powder (1.71 g, 26.34 mmol, 2.0 equiv.) in glacial AcOH (50 mL) was added *rac*-(1*R*,6*R*)-7,7-Dichlorobicyclo[4.3.0]non-3-en-8-one **S3** (2.7 g, 13.17 mmol, 1.0 equiv.) in glacial AcOH (30 mL) dropwise. The resulting reaction mixture was stirred for 16 h at 70 °C. The reaction mixture was then cooled to room temperature and filtered to remove the resulting solid. The filtrate was diluted with CH_2Cl_2 (200 mL) and washed with H_2O (2 x 300 mL), sat. aq. $NaHCO_3$ (1 x 300 mL), brine (1 x 300 mL), dried with $MgSO_4$, filtered and concentrated *in vacuo*. The resulting crude pale yellow oil was purified by column chromatography on silica (hexane/EtOAc 95:5) to give the *meso* compound as a colourless oil. All spectroscopic and physical data were in full agreement with those reported in the literature.[6] Yield: 2.45 g (72%); ^{1}H NMR (400 MHz, $CDCl_3$) δ 5.70-5.69 (m, 2H), 2.46-2.41 (m, 2H), 2.34-2.25 (m, 4H), 2.10 (dd, *J* = 6.4, 18.6 Hz, 2H) 1.89-1.83 (m, 2H); ^{13}C NMR (100 MHz, $CDCl_3$) δ 219.6 124.6 (2C), 44.6 (2C), 32.3 (2C), 26.2 (2C); IR (neat, cm^{-1}) 3024 (m), 2834 (m), 2901 (s), 1744 (s), 1655 (w), 1439 (m), 1407 (s); HRMS (EI+): Exact mass calculated for $C_9H_{12}O$ $[M]^+$: 136.0888, found 136.0983; TLC (hexane/EtOAc 4:1, $KMnO_4$ stain): R_f = 0.51.

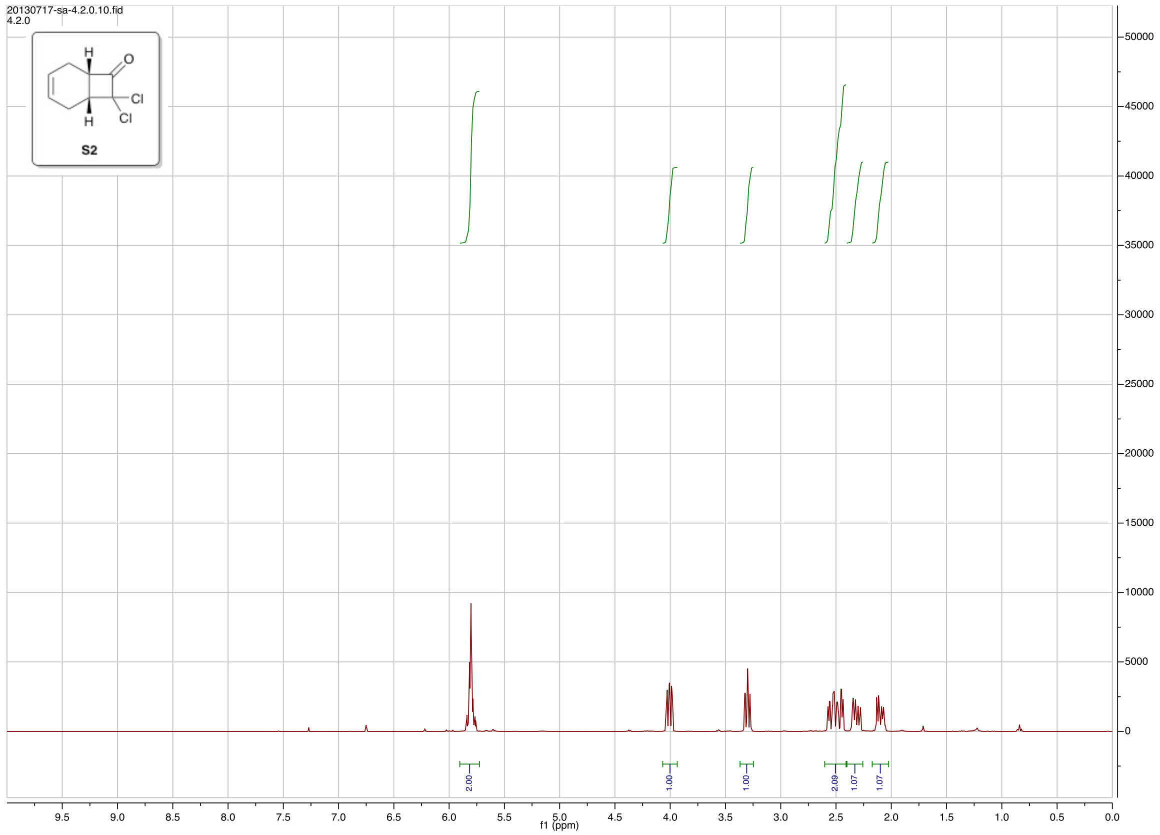

Figure S-1 ^{1}H-NMR spectrum of compound **S2**.

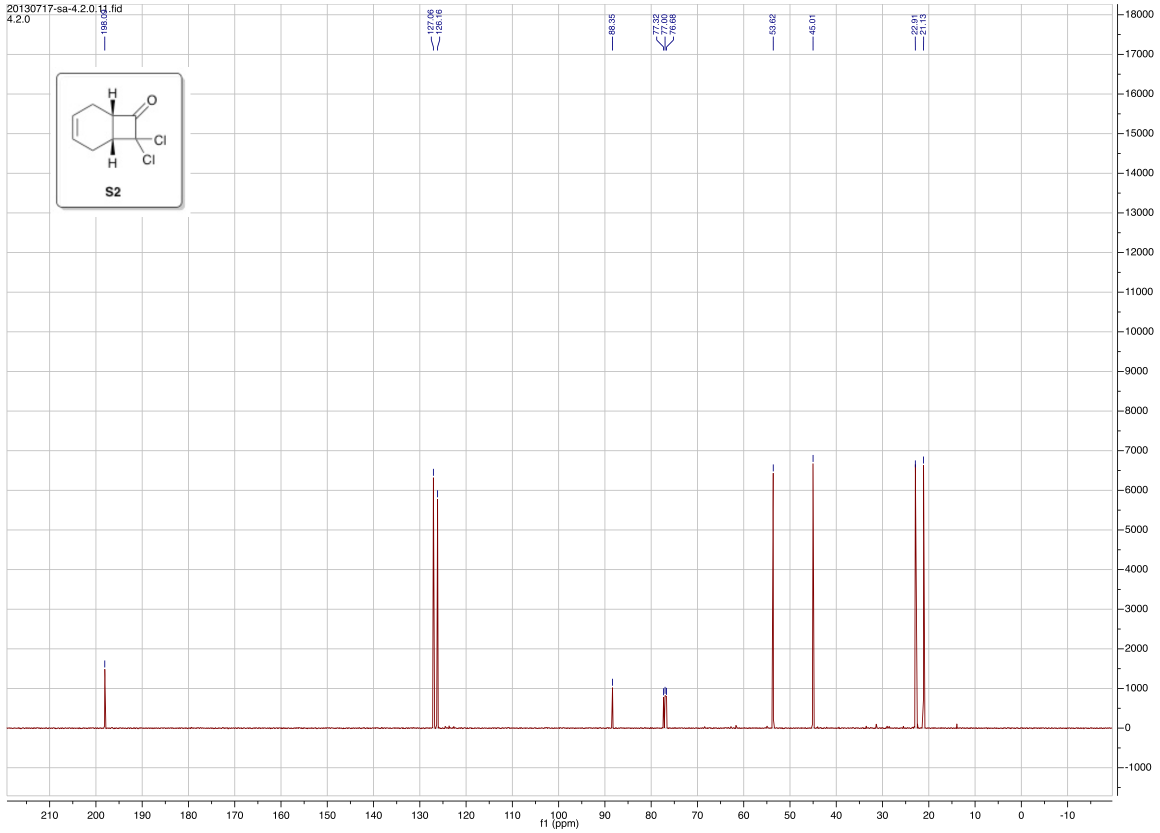

Figure S-2 ^{13}C-NMR spectrum of compound **S2**.

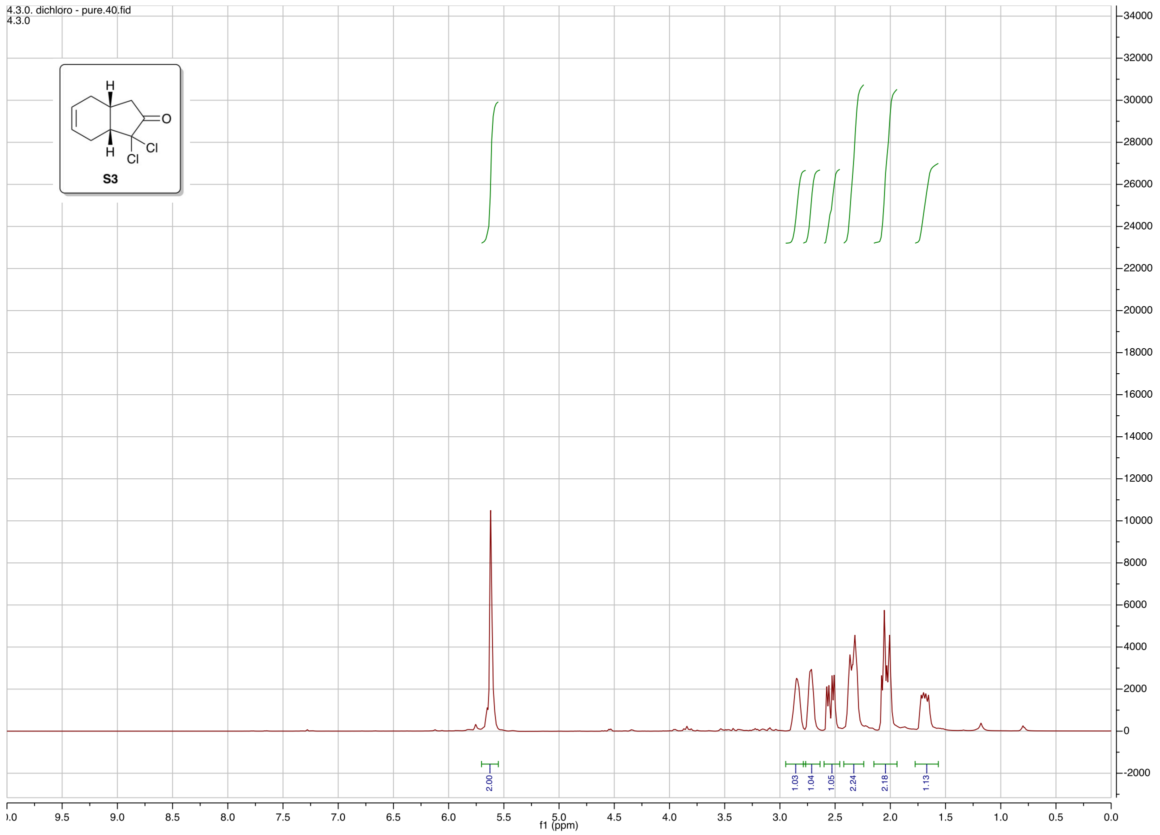

Figure S-3 ^{1}H-NMR spectrum of compound **S3**.

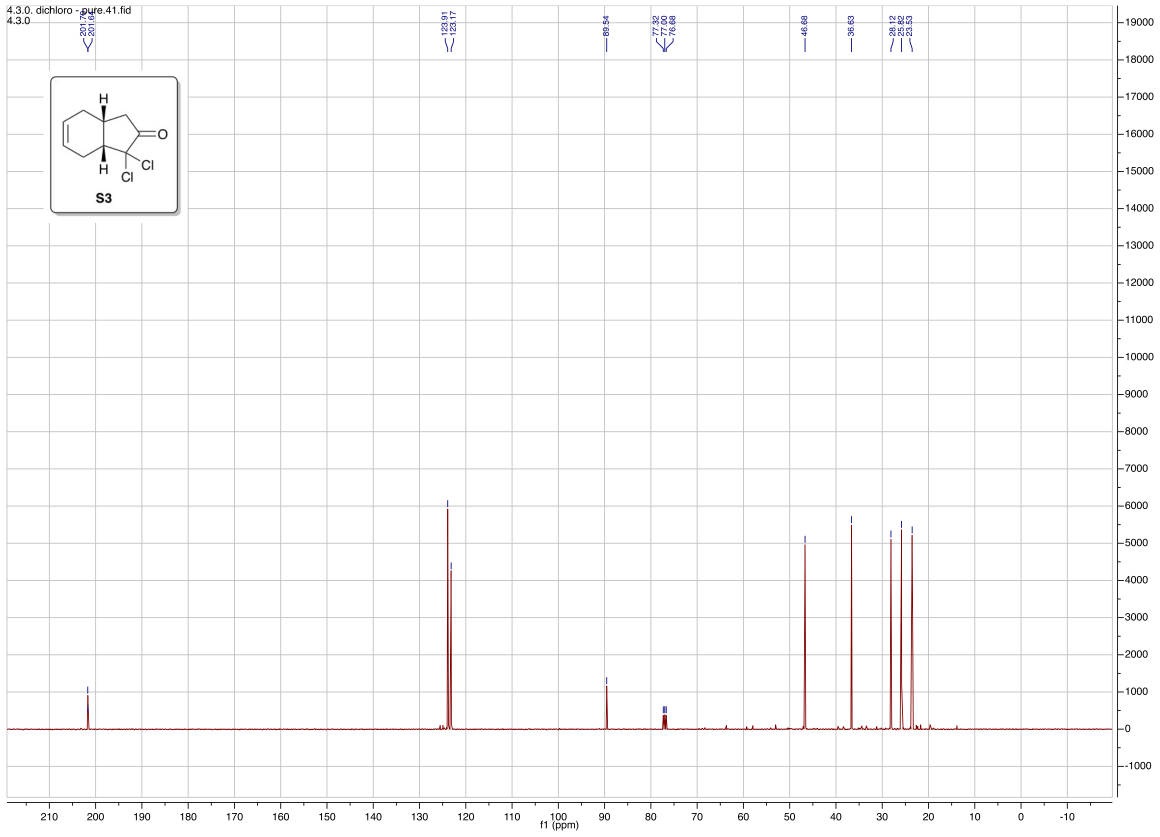

Figure S-4 ^{13}C-NMR spectrum of compound **S3**.

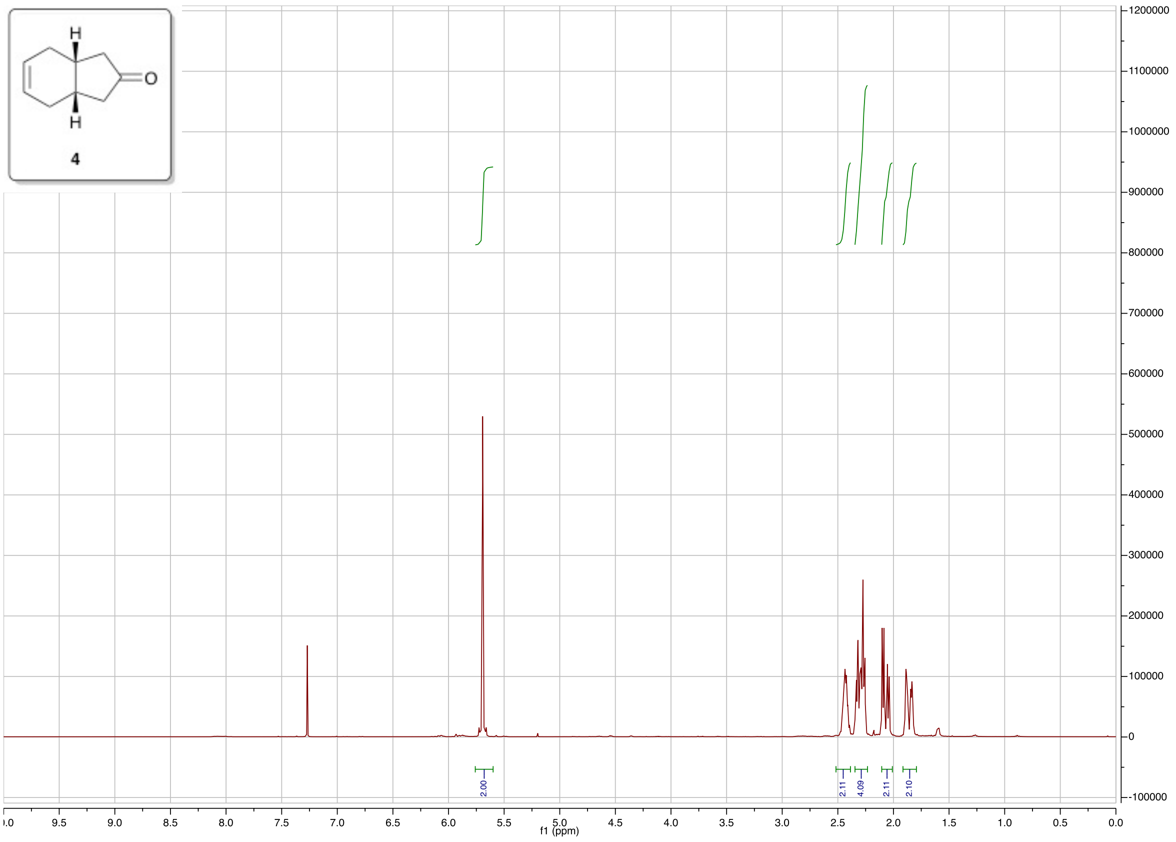

Figure S-5 ^{1}H-NMR spectrum of compound **4**.

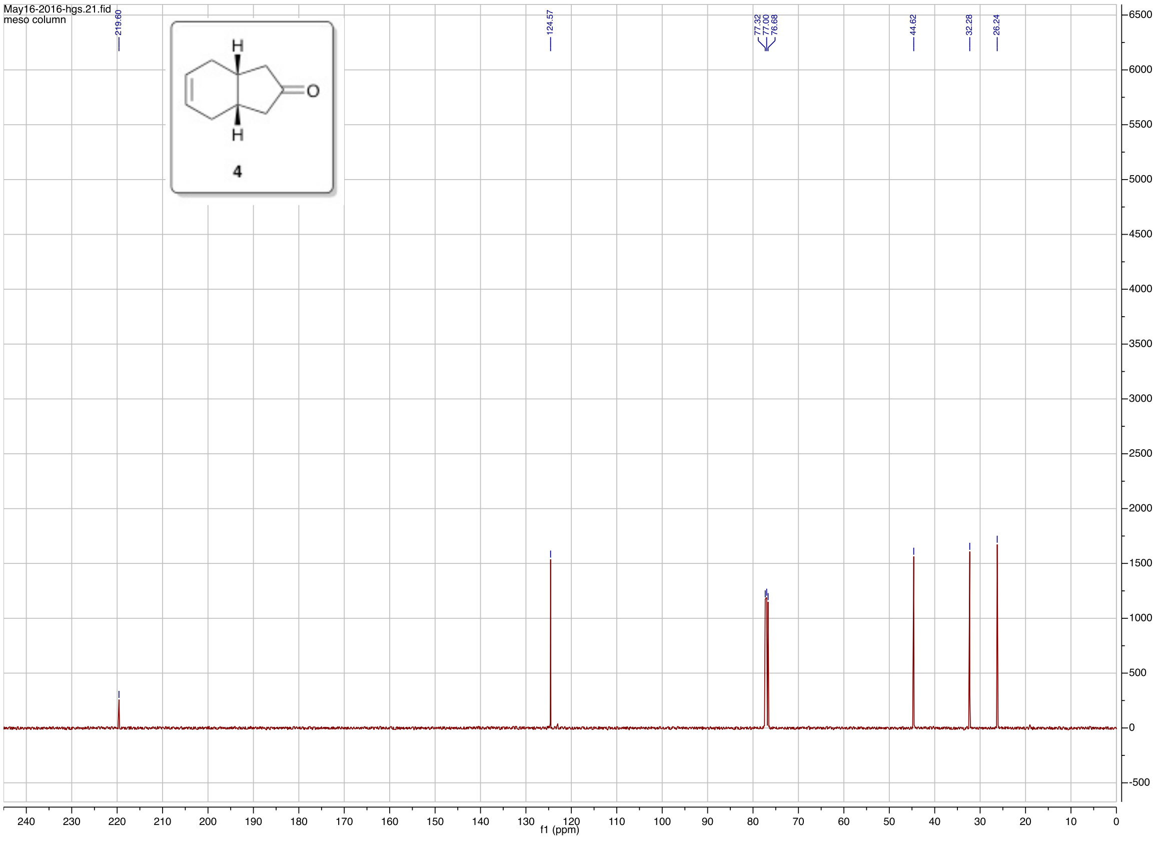

Figure S-6 ^{13}C-NMR spectrum of compound **4**.

Elemental Composition Report

Page 1

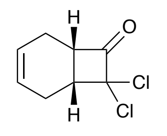

S2

Single Mass Analysis
Tolerance = 10.0 PPM / DBE: min = -1.5, max = 50.0
Isotope cluster parameters: Separation = 1.0 Abundance = 1.0%

Monoisotopic Mass, Odd and Even Electron Ions
44 formula(e) evaluated with 1 results within limits (up to 50 closest results for each mass)

Sample 1 C8OCl2H8 MW 191
DEHGS2016021901 540 (6.602)

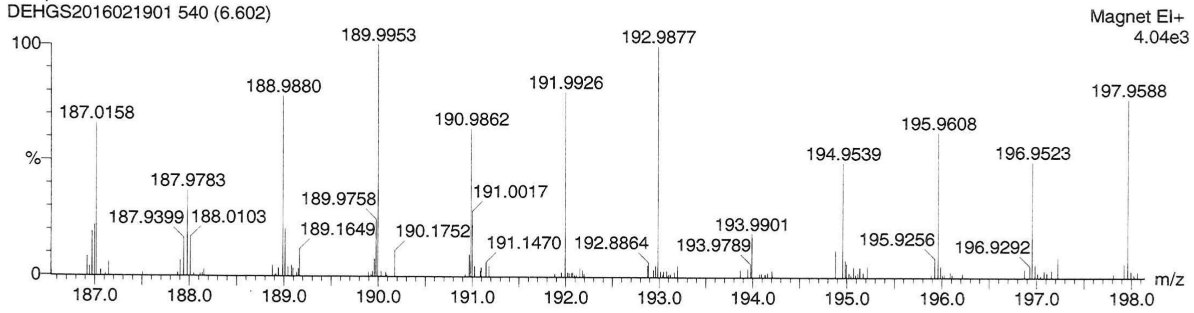

Minimum:				-1.5		
Maximum:		200.0	10.0	50.0		
Mass	Calc. Mass	mDa	PPM	DBE	Score	Formula
189.9953	189.9952	0.1	0.4	4.0	1	C8 H8 O Cl2

Figure S-7 HRMS of compound **S2**.

Elemental Composition Report

Page 1

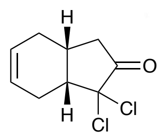

S3

Single Mass Analysis
Tolerance = 10.0 PPM / DBE: min = -1.5, max = 50.0
Isotope cluster parameters: Separation = 1.0 Abundance = 1.0%

Monoisotopic Mass, Odd and Even Electron Ions
48 formula(e) evaluated with 1 results within limits (up to 50 closest results for each mass)

Sample 2 C9H10OCl2 MW 205
DEHGS2016021902 515 (12.982)

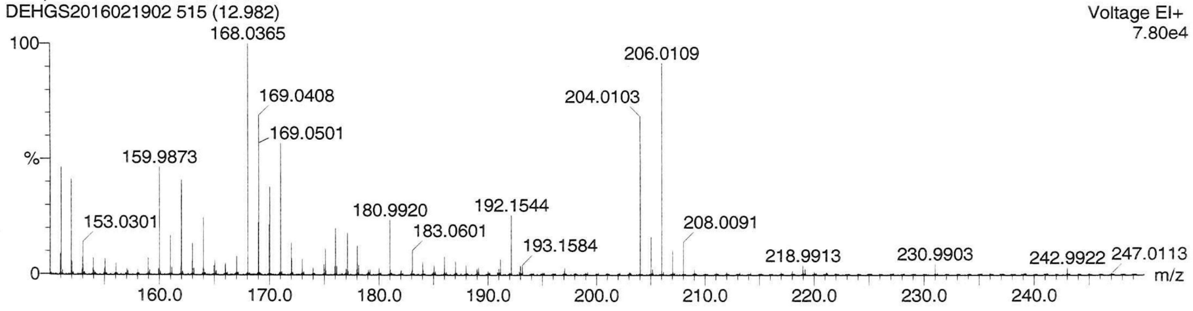

Minimum:				-1.5		
Maximum:		200.0	10.0	50.0		
Mass	Calc. Mass	mDa	PPM	DBE	Score	Formula
204.0103	204.0109	-0.6	-2.8	4.0	1	C9 H10 O Cl2

Figure S-8 HRMS of compound **S3**.

Elemental Composition Report

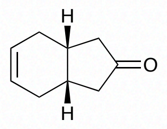

4

Page 1

Single Mass Analysis

Tolerance = 10.0 PPM / DBE: min = -1.5, max = 50.0
Isotope cluster parameters: Separation = 1.0 Abundance = 1.0%

Monoisotopic Mass, Odd and Even Electron Ions
11 formula(e) evaluated with 1 results within limits (up to 50 closest results for each mass)

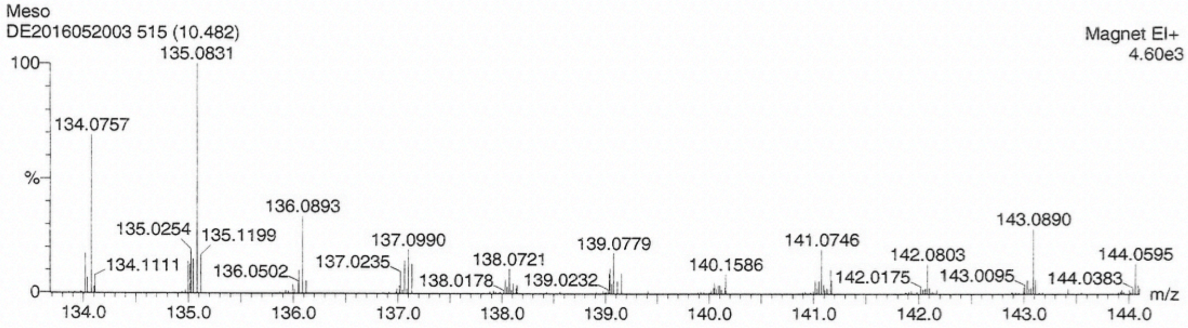

Minimum:				-1.5		
Maximum:		200.0	10.0	50.0		
Mass	Calc. Mass	mDa	PPM	DBE	Score	Formula
136.0893	136.0888	0.5	3.6	4.0	1	C9 H12 O

Figure S-9 HRMS of compound **4**.

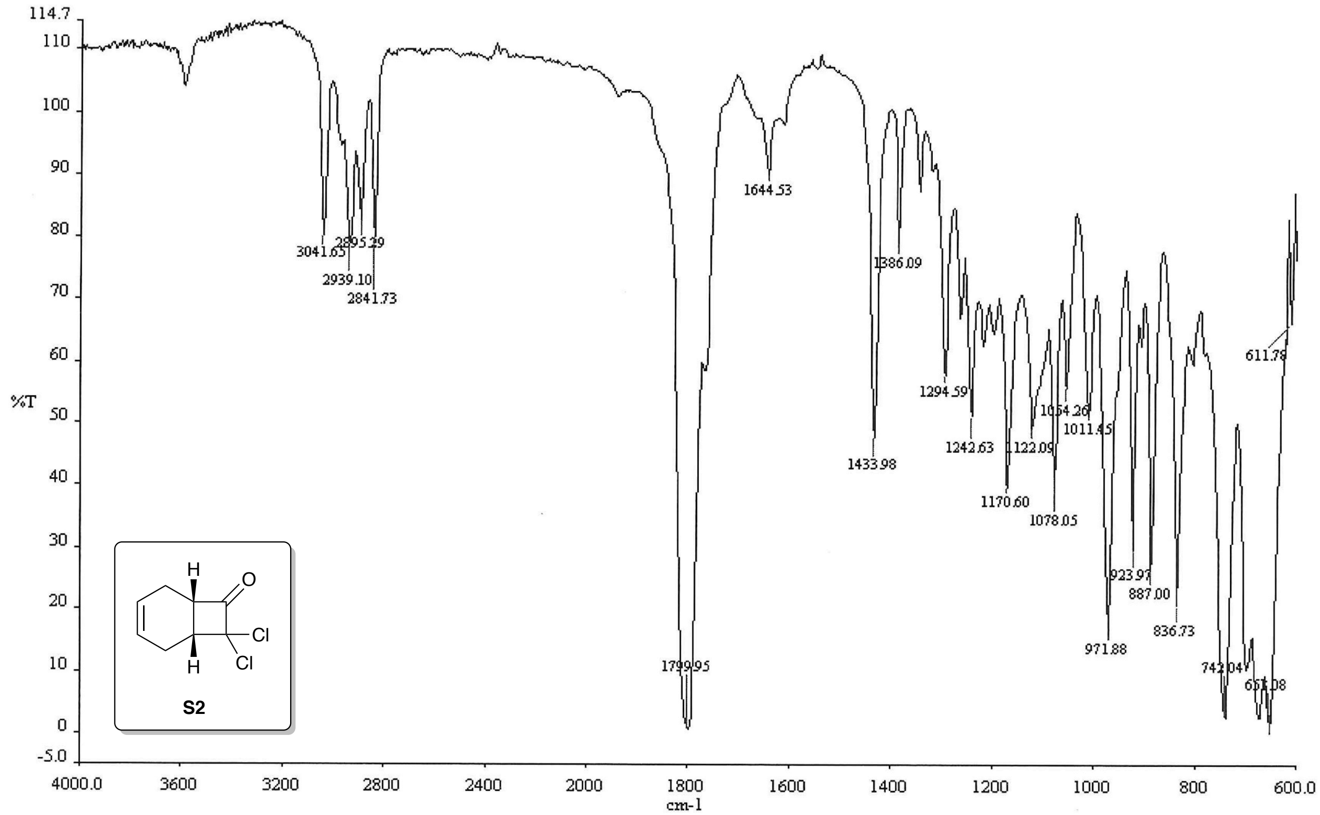

Figure S-10 IR of compound **S2**.

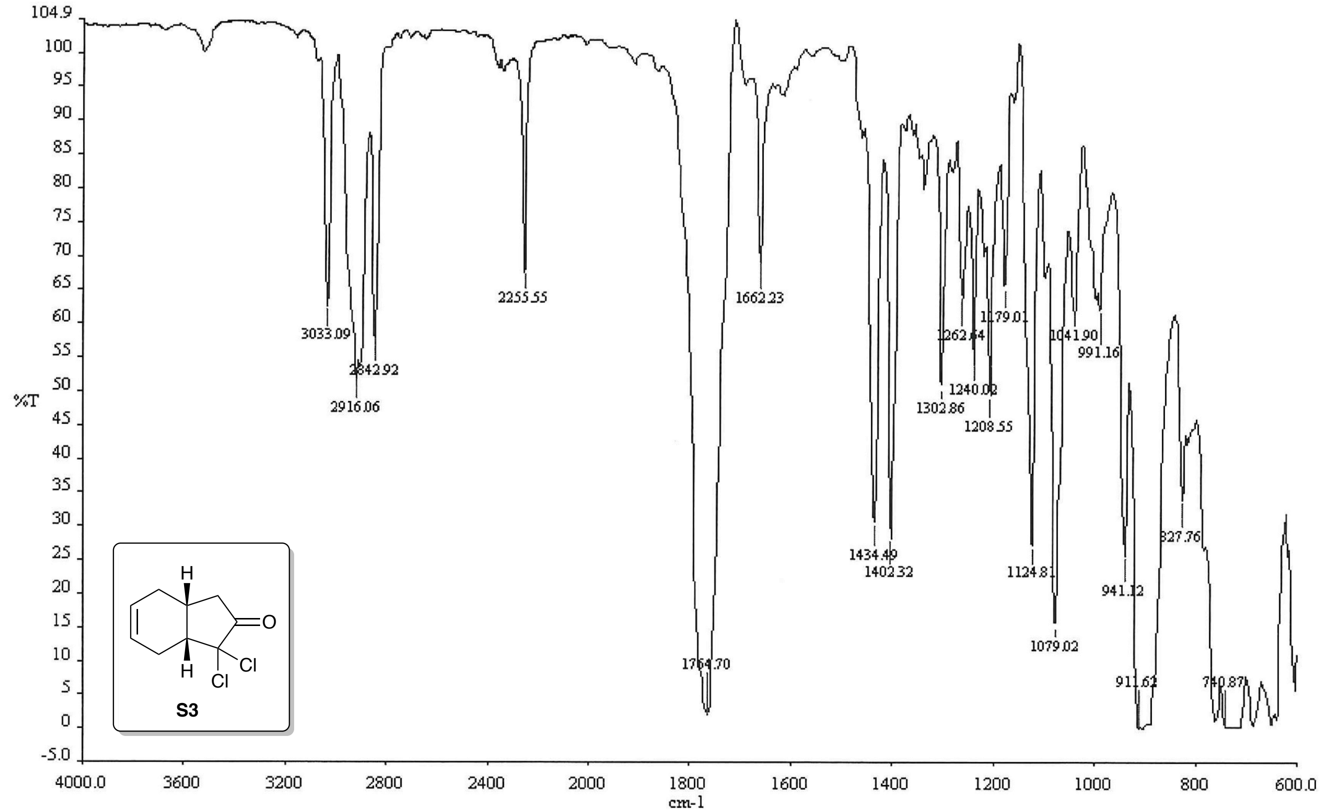

Figure S-11 IR of compound **S3**.

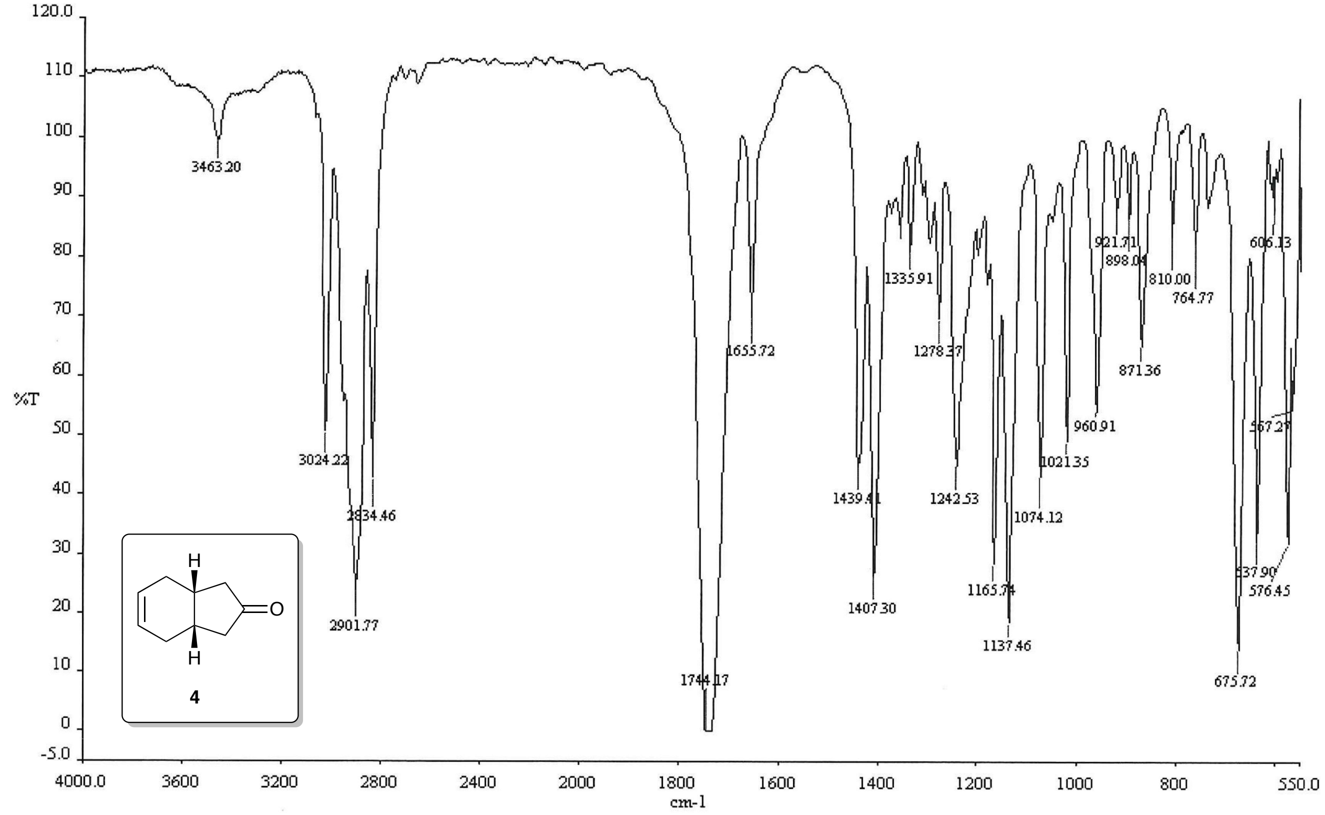

Figure S-12 IR of compound **4**.

Preparation of *exo*-mucosin (1*) and the methyl ester (2*):

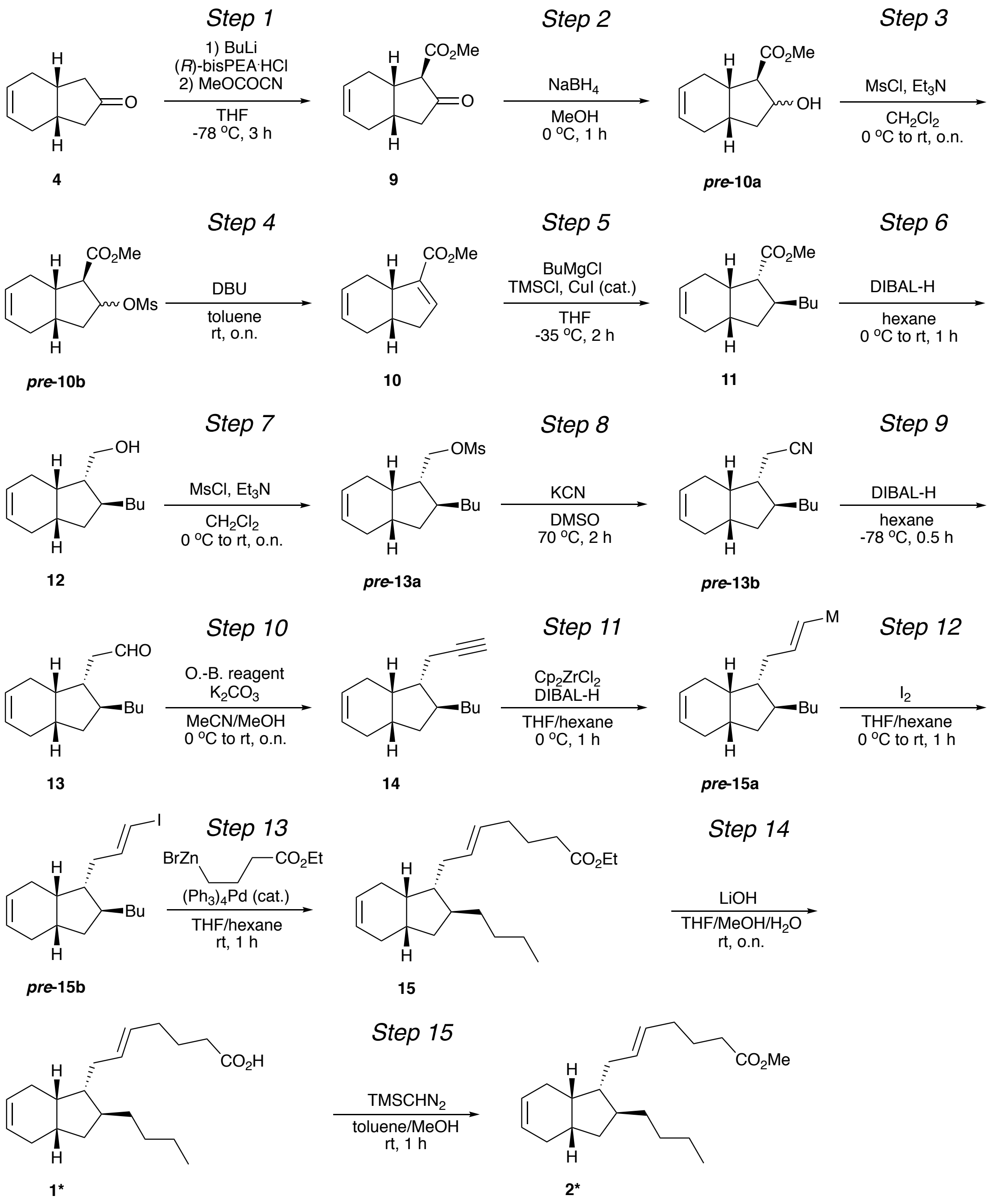

Scheme S-2 Synthetic route to *exo*-mucosin **1*** and its methyl ester **2***.

Methyl (1*S*,6*S*,7*R*)-8-oxobicyclo[4.3.0]non-3-ene-7-carboxylate (9).[7]

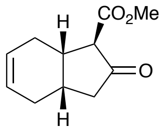

9

(+)-Bis[(*R*)-1-phenylethyl]amine hydrochloride (2.5 g, 9.60 mmol, 1.58 equiv.) was added in one portion to dry THF (10 mL) at room temperature and stirred for 5 min. The stirring suspension was then cooled to -78 °C and BuLi (2.5M in hexane) (7.67 mL, 19.18 mmol, 3.16 equiv.) was added dropwise. The suspension changed colour from cloudy white to pale orange. After stirring at -78 °C for 15 min the suspension was warmed to room temperature whereby a transparent yellow solution was formed. This was then cooled to -78 °C again and *meso*-(1*S*,6*R*)-bicyclo[4.3.0]non-3-ene-8-one **4** (0.826 g, 6.07 mmol, 1.0 equiv.) was added dropwise over 10 min in dry THF (10 mL). This mixture was then stirred for 45 min whereby a purple colour evolved. Methyl cyanoformate (0.96 mL, 12.14 mmol, 2.0 equiv.) was then added dropwise over 5 min. and the mixture immediately turned bright yellow in colour. This mixture was left stirring for 2.5 h and then quenched by addition of H_2O (2 mL) at -78 °C. The mixture was then warmed to r.t and extracted with EtOAc (2 x 50 mL). The resulting organic layer was then washed with H_2O (2 x 100 mL), 0.5 M HCl (1 x 100 mL) and brine (1 x 100 mL). The organic layer was then dried over $MgSO_4$, filtered and concentrated *in vacuo*. The resulting crude keto-ester was purified by column chromatography (hexane/EtOAc 5:1) to form a colourless oil. This oil was then recrystallised from hexane at 0°C, filtered and air dried to obtaine the title compound as white crystals. All spectroscopic and physical data were in full agreement with those reported in the literature.[7] Yield: 0.812 g (69%); $[\alpha]_D^{26} - 161$ (c = 0.1, $CHCl_3$); ^{1}H NMR (400 MHz, $CDCl_3$) δ 5.73-5.66 (m, 2H), 3.76 (s, 3H), 3.04 (d, *J* = 11.1 Hz, 1H), 2.88-2.83 (m, 1H), 2.52-2.38 (m, 3H), 2.33-2.21 (m, 2H), 2.04 (dd, *J* = 1.9, 18.2 Hz, 1H), 1.67-1.61 (m, 1H); ^{13}C NMR (100 MHz, $CDCl_3$) δ 211.6, 169.7, 124.9, 123.9, 57.7, 52.4, 46.6, 37.3, 29.7, 26.8, 25.3; IR (neat, cm^{-1}) 3034 (w), 2945 (m), 2908 (m), 2837 (w), 1751 (s), 1718 (s) 1656 (w) 1433 (s) 1404 (m); HRMS (EI+): Exact mass calculated for $C_{11}H_{14}O_3$ $[M]^+$: 194.0943, found 194.0933; m.p.: 59-61 °C; TLC (hexane/EtOAc 4:1, $KMnO_4$ stain): R_f = 0.42.

***rac*-Methyl (1*S*,6*S*,7*R*)-8-oxobicyclo[4.3.0]non-3-ene-7-carboxylate (9).**

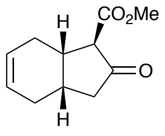

9

LDA (1M in THF/Hexanes) (1.65 mL, 1.65 mmol, 1.5 equiv.) was added dropwise to dry THF (5 mL) at -78 °C and stirred for 30 min. Then *meso*-(1*S*,6*R*)-bicyclo[4.3.0]non-3-ene-8-one **4** (0.150 g, 1.10 mmol, 1.0 equiv.) was added dropwise in dry THF (5 mL) over 5 min and left to stir for 45 min. To the resulting yellow solution was added methyl cyanoformate (0.174 mL, 2.2 mmol, 2.0 equiv.) dropwise over 5 min and the reaction changed from yellow to colourless. After 30 min and monitoring the reaction via TLC the reaction was quenched at -78 °C by sat. aq. NH_4Cl (2 mL) and the reaction mixture was left to slowly warm to room temperature. The reaction mixture was then poured over H_2O (1 x 20 mL) and the organic phase separated. The aqueous phase was then extracted with EtOAc (2 x 20 mL). The organic phases were then combined, washed with H_2O (2 x 50 mL), brine (1 x 50 mL), dried over $MgSO_4$, filtered and concentrated *in vacuo* to from a crude yellow oil. This yellow oil was purified by column chromatography on silica (hexane/EtOAc, 5:1) to afford the racemic keto-ester. This was recrystallised in the same fashion as the optically active keto ester to afford pure white crystals. Yield: 0.166 g, (78%).

The material was used in the preparation of racemic reference material for chiral GLC analysis.

Methyl (1*S*,6*S*,7*R*,8*RS*)-8-hydroxybicyclo[4.3.0]non-3-ene-7-carboxylate (*pre*-10a).

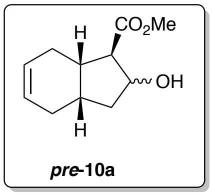

To stirring solution of methyl (1*S*,6*S*,7*R*)-8-oxobicyclo[4.3.0]non-3-ene-7-carboxylate **9** (1.40 g, 7.21 mmol, 1.0 equiv.) in MeOH (50 mL) at 0 °C was added $NaBH_4$ (0,410 g, 10.8 mmol, 1.5 equiv.). The reaction was monitored by TLC and was deemed complete after 1 h. Then, dilute aq. HCl (10 mL, 1 M) was added drowise at 0 °C. The quenched reaction was concentrated *in vacuo* to afford a crude mixture. This was poured over Et_2O (50 mL), whereupon water (50 mL) was added. The organic layer was separated and the aqueous layer was extracted with Et_2O (2 x 50 mL). The organic layers were combined, washed with brine (1 x 100 mL), dried over $MgSO_4$, filtered and concentrated *in vacuo* to afford a colourless, oily, residue. The crude was purified by column chromatography on silica (hexane/EtOAc 7:3) to afford the C8-epimeric title compound as colourless oil. Yield: 1.07 g (76%); ^{1}H NMR (400 MHz, $CDCl_3$) δ 5.81-5.71 (m, 2H), 4.57-4.39 (m, 1H), 3.73 (s, 3H), 2.65-2.50 (m, 1H), 2.38-2.10 (m, 6H), 2.10-1.84 (m, 2H), 1.52-1.42 (m, 1H); ^{13}C NMR (100 MHz, $CDCl_3$) δ 175.5 (major), 174.8 (minor), 127.5 (minor), 126.7 (minor), 126.6 (major), 125.6 (major), 75.3 (major), 73.0 (minor), 57.3 (major), 53.9 (minor), 51.8 (major), 51.7 (minor), 42.1 (minor), 40.9 (major), 39.0 (major), 38.0 (minor), 34.0 (minor), 33.6 (major), 27.9 (minor), 27.7 (major), 26.3 (major), 26.2 (minor); IR (neat, cm^{-1}) 3439 (br), 3026 (w), 2914 (w), 2841 (w), 1712 (s), 1438 (m); HRMS (EI+): Exact mass calculated for $C_{11}H_{16}O_3$ $[M]^+$: 196.1099, found 196.1087; TLC (hexane/EtOAc 4:1, $KMnO_4$ stain): R_f = 0.30.

Following the same procedure as above, racemic synthesis was performed to obtain reference material for chiral GLC analysis.

Methyl (1*S*,6*S*,7*R*,8*RS*)-8-((methylsulfonyl)oxy)bicyclo[4.3.0]non-3-ene-7-carboxylate (*pre*-10b).

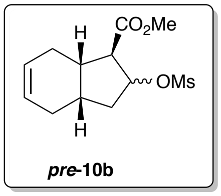

To stirring solution of methyl (1*S*,6*S*,7*R*,8*RS*)-8-hydroxybicyclo[4.3.0]non-3-ene-7-carboxylate ***pre*-10a** (1.07 g, 5.45 mmol, 1.0 equiv.) in dry CH_2Cl_2 (50 mL) at ambient temperature was added Et_3N (1.14 ml, 8.18 mmol, 1.5 equiv.) in a dropwise manner. The resulting mixture was left stirring for 5 min. and then cooled to 0 °C. Subsequently, methanesulfonyl chloride (0.51 mL, 6.59 mmol, 1.2 equiv.) was added in a dropwise manner and the reaction mixture was left stirring for 10 min. with continued cooling. Then, the cooling was discontinued and the reaction mixture was allowed to attain ambient temperature over night. At this point the reaction mixture had turned from colourless to yellow. Brine (20 mL) was added in a dropwise manner and the volatiles were removed *in vacuo*. The resulting yellow liquid was poured over EtOAc (50 mL) and satd. aq. $NaHCO_3$ (50 mL) was added. The organic layer was separated and the aqueous layer was extracted with EtOAc (2 x 50 mL). The organic layers were combined and washed with brine (1 x 100 mL), dried over $MgSO_4$, filtered and concentrated *in vacuo* to afford a yellow, oily, residue. The crude was purified by column chromatography on silica (hexane/EtOAc 4:1) to afford the C8-epimeric title compound as a yellow oil. Yield: 1.16 g (77%); ^{1}H NMR (400 MHz, $CDCl_3$) δ 5.81-5.77 (m, 0.3H, minor), (m, 1.7H, major), 5.37-5.31 (m, 1H), 3.74 (s, 2.6H, major), 3.72 (s, 0.4H, minor), 3.01 (s, 2.6H, major), 2.96 (s, 0.4H, minor), 2.88-2.82 (m, 1H), 2.40-2.06 (m, 6H), 2.02-1.87 (m, 1H), 1.86-1.78 (m, 1H); ^{13}C NMR (100 MHz, $CDCl_3$) δ 173.9, 127.3 (minor), 126.8 (minor), 125.6 (major), 124.4 (major), 83.9 (major), 83.8 (minor), 54.6 (major), 53.6 (minor), 52.2 (major), 51.9 (minor), 40.9 (minor), 39.8 (major), 39.1 (major), 38.3 (minor), 37.9 (major), 36.4 (minor), 33.9 (major), 33.5 (minor), 27.6 (minor), 26.6

(major), 26.0 (minor), 25.4 (major); IR (neat, cm^{-1}) 3031 (w), 2942 (w), 2847 (w), 1734 (s), 1438 (w), 1354 (s); HRMS (EI+): Exact mass calculated for $C_{12}H_{18}O_5S$ $[M]^+$: 274.0875, found 274.0865; TLC (hexane/EtOAc 4:1, $KMnO_4$ stain): R_f = 0.45.

Following the same procedure as above, racemic synthesis was performed to obtain reference material for chiral GLC analysis.

Methyl (1*S*,6*S*)-bicyclo[4.3.0]non-3,7-diene-7-carboxylate (10).

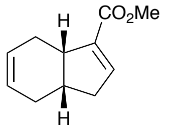

10

To a stirring solution of methyl (1*S*,6*S,*7*R*,8*RS*)-8-((methylsulfonyl)oxy)bicyclo[4.3.0]non-3-ene-7-carboxylate ***pre*-10b** (1.40 g, 5.10 mmol, 1.0 equiv.) in dry toluene (30 mL) at ambient temperature was added DBU (1.73 mL, 11.6 mmol, 2.3 equiv.) in a dropwise manner over 5 min. The reaction mixture was stirred over night at the stated conditions. Having deemed the reaction complete by TLC, water (10 mL) and dilute aq. HCl (10 ml, 0.5 M) was added. The resulting mixture was poured over Et_2O (20 mL) and the organic layer was separated. The aqueous layer was extracted with Et_2O (2 x 30 mL). The organic layers were combined, washed in succession with water (1 x 50 mL) and brine (1 x 50 mL), dried over $MgSO_4$, filtered and concentrated *in vacuo* to obtain a oily residue. The crude was purified by column chromatography on silica (hexane/EtOAc 9:1) to afford the title compound as colourless oil. Yield: 0.866 g, (95%); $[\alpha]_D^{26} + 180$ (c = 0.8, $CHCl_3$); ^1H NMR (400 MHz, $CDCl_3$) δ 6.80-6.69 (m, 1H), 5.95-5.84 (m, 1H), 5.84-5.74 (m, 1H), 3.74 (s, 3H), 3.04-3.92 (m, 1H), 2.67-2.51 (m, 2H), 2.50-2.36 (m, 1H), 2.34-2.12 (m, 2H), 2.00-1.82 (m, 2H); ^{13}C NMR (100 MHz, $CDCl_3$) δ 165.6, 143.5, 140.8, 128.3, 127.0, 51.2, 41.2, 39.4, 36.0, 27.8, 26.6; IR (neat, cm^{-1}) 3031 (w), 2931 (w), 2841 (w), 1712 (s), 1628 (w), 1438 (m); HRMS (EI+): Exact mass calculated for $C_{11}H_{14}O_2$ $[M]^+$: 178.0994, found 178.1000; TLC (hexane/EtOAc 4:1, $KMnO_4$ stain): R_f = 0.60.

Following the same procedure as above, racemic synthesis was performed to obtain reference material for chiral GLC analysis.

Methyl (1*S*,6*S*,7*R*,8*S*)-8-butylbicyclo[4.3.0]non-3-ene-7-carboxylate (11).

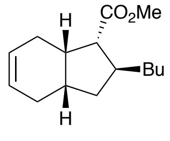

11

To a solution of methyl (1S,6S)-bicyclo[4.3.0]non-3,7-diene-7-carboxylate **10** (0.421 g, 2.36 mmol, 1.0 equiv.) in dry THF (20 mL) at -35 °C was added in succession CuI (0.045 g, 0.24 mmol, 0.1 equiv.) and TMSCl (0.641 g, 0.75 mL, 5.90 mmol, 2.5 equiv.). The resulting slightly heterogenous mixture caused by suspended CuI was stirred for 5 min, whereupon BuMgCl (2.0 M in THF) (2.36 mL, 4.72 mmol, 2.0 equiv.) was added in a dropwise manner during the course of 2h, maintaining the temperature between at -35 °C. Initial colours cycled between clear and yellow, but gradually took a transient purple hue while reverting to clear. Upon completing the addition, the purple colour persisted (cloudy amethyst). At this point TLC revealed that the starting material had been consumed. The reaction was treated with aq. satd. NH_4Cl (5 mL) and diluted with Et_2O/water (30 mL, 2:1). The phases were separated and the aq. phase was extracted with Et_2O (3 x 20 mL). The combined org. phases were washed with brine (15 ml), dried over $MgSO_4$, filtered and the solvent was evaporated *in vacuo*. The residue was purified by column chromatography on silica (hexane/EtOAc 90:10) to afford the title compound as a colourless oil. Yield: 0.496 g (81%); $[\alpha]_D^{26} + 63$ ($c = 0.8$, $CHCl_3$); ^1H NMR (400 MHz, $CDCl_3$) δ 5.66-5.56 (m, 2H), 3.67 (s, 3H),

2.58-2.46 (m, 2H), 2.36-2.16 (m, 3H), 1,95-1.68 (m, 4H), 1.45-1.35 (m, 2H), 1.35-1.15 (m, 5H), 0.87 (t, J = 7.0 Hz, 3H); ^{13}C NMR (100 MHz, $CDCl_3$) δ 174.8, 124.7, 124.6, 56.0, 51.3, 38.9, 37.4, 36.8, 36.0, 35.3, 30.4, 26.5, 22.8, 22.6, 14.1; IR (neat, cm^{-1}) 3020 (w), 2925 (m), 1734 (s); HRMS (EI+): Exact mass calculated for $C_{15}H_{24}O_2$ $[M]^+$: 236.1776, found 236.1763; TLC (hexanes/EtOAc 80:20, $KMnO_4$ stain): R_f = 0.70.

Following the same procedure as above, racemic synthesis was performed to obtain reference material for chiral GLC analysis.

(1*S*,6*S*,7*R*,8*S*)-8-Butyl-7-(hydroxymethyl)bicyclo[4.3.0]non-3-ene (12).

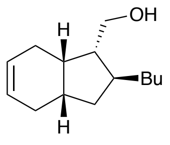

12

Methyl (1*S*,6*S*,7*R*,8*S*)-8-butylbicyclo[4.3.0]non-3-ene-7-carboxylate **11** (0.496 g, 2.10 mmol, 1.0 equiv.) was dissolved in hexane (10 mL) at room temperature and stirred for 5 min. The solution was then cooled to 0 °C and DIBAL-H (1M in hexane) (4.2 mL, 4.20 mmol, 2.0 equiv.) was added dropwise over 5 min. The reaction was then left to warm to r.t. After 1 h the reaction was cooled back to 0 °C and quenched with sat. aq. NH_4Cl (5 mL). The reaction mixture was allowed to warm to room temperature whereby a cloudy suspension occurred. This suspension was poured over sat. aq. NH_4Cl (20 mL) and the organic layer separated. The aqueous layer was extracted with EtOAc (2 x 50 mL) and the organic layers combined, washed with H_2O (1 x 100 mL), brine (1 x 100 mL), dried over $MgSO_4$, filtered and concentrated *in vacuo* to give a crude cloudy oil. This was then purified by column chromatography on silica (hexane/EtOAc 95:5) to afford the title compound as a colourless oil. Yield: 0.400 g, (92%); $[\alpha]_D^{26} + 104$ ($c\ =\ 0.8$, $CHCl_3$); ^{1}H NMR (400 MHz, $CDCl_3$) 5.77-5.51 (m, 2H), 3.77-3.58 (m, 2H), 2.38-2.22 (m, 1H), 2.22-2-05 (m, 2H), 2.05-1.71 (m, 4H) 1.71-1.52 (m, 2H), 1.52-1.35 (2H) 1.35-1.14 (m, 6H), 0.88 (t, J = 7. Hz, 3H); ^{13}C NMR (100 MHz, $CDCl_3$) δ 125.3, 124.9, 63.3, 53.7, 38.1, 36.8, 36.3, 35.5, 35.4, 30.8, 26.6, 22.9, 21.6, 14.1; IR (neat, cm^{-1}) 3328 (br.), 3020 (w), 2925 (s); HRMS (EI+): Exact mass calculated for $C_{14}H_{24}O$ $[M]^+$: 208.1827, found 208.1832; TLC (hexane/EtOAc 4:1, $KMnO_4$ stain): R_f = 0.40. The enantiomeric excess was determined by chiral GLC analysis (CP-Chirasil-DEX CB, using the following program: 60 ^{o}C (45 min) - 1 degrees/min to 160 ^{o}C - 160 ^{o}C (5 min)): $t_r(e_1$, major) = 65.08 min and $t_r(e_2$, minor) = 65.67 min; *ee*: > 99%.

Following the same procedure as above, racemic synthesis was made to obtain reference material for chiral GLC analysis.

(1*S*,6*S*,7*R*,8*S*)-8-Butyl-7-((methylsulfonyl)oxymethyl)bicyclo[4.3.0]non-3-ene (*pre*-13a).

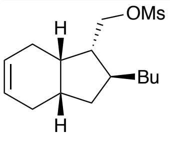

***pre*-13a**

To a stirring solution of (1*S*,6*S*,7*R*,8*S*)-8-butyl-7-(hydroxymethyl)bicyclo[4.3.0]non-3-ene **12** (0.400 g, 1.92 mmol, 1.0 equiv.) in dry CH_2Cl_2 (5 mL) at room temperature, was added Et_3N (0.54 mL, 3.84 mmol, 2.0 equiv.) dropwise. This solution was left stirring for 5 min then cooled to 0 °C. Then methanesulfonyl chloride (0.45 mL, 5.76 mmol, 3.0 equiv.) was added dropwise and the reaction was left at 0 °C for 10 min then warmed to room temperature and left for over night. The reaction mixture turned colourless to yellow. Then, brine (10 mL) was added dropwise and the volatiles concentrated *in vacuo* to afford a yellow liquid. This was poured over EtOAc (50 mL) and sat. aq. $NaHCO_3$ (50 mL) was added. The organic layer was separated and the aqueous layer extracted with EtOAc (2 x 50 mL). The organic layers were combined and washed with brine (1 x 50 mL), dried

over $MgSO_4$, filtered and concentrated *in vacuo* to afford a crude yellow oil. This was then purified by column chromatography on silica (hexane/EtOAc 95:5) to afford the title compound as a colourless oil. Yield: 0.497 g, (90%); $[\alpha]_D^{26} + 79$ ($c = 0.8$, $CHCl_3$); ^1H NMR (400 MHz, $CDCl_3$) δ 5.60-5.50 (m, 2H), 4.21-4.12 (m, 2H), 2.94 (s, 3H), 2.25-2.11 (m, 1H), 2.11-1.99 (m, 2H), 1.98-1.83 (m, 2H), 1.83-1.76 (m, 1H), 1.71-1.55 (m, 3H), 1.40-1.30 (m, 2H), 1.28- 1.10 (m, 5H), 0.82 (t, *J* = 7.0 Hz, 3H); ^{13}C NMR (100 MHz, $CDCl_3$) δ 125.3, 124.4, 70.3, 50.0, 38.1, 37.4, 36.6, 36.4, 35.4, 35.3, 30.7, 26.4, 22.8, 21.4, 14.1; IR (neat, cm^{-1}) 3020 (w), 2925 (m), 1354 (s); HRMS (EI+): Exact mass calculated for $C_{15}H_{26}O_3S_2$ $[M]^+$: 286.1603, found 286.1627; TLC (hexane/EtOAc 4:1, $KMnO_4$ stain): R_f = 0.50.

(1*S*,6*S*,7*R*,8*S*)-8-Butyl-7-(cyanomethyl)bicyclo[4.3.0]non-3-ene (*pre*-13b).

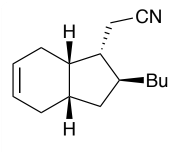

***pre*-13b**

To a stirring solution of (1*S*,6*S*,7*R*,8*S*)-8-butyl-7-((methylsulfonyl)oxymethyl)bicyclo[4.3.0]non-3-ene ***pre*-13a** (0.497 g, 1.74 mmol, 1.0 equiv.) in dry DMSO (30 mL) was added solid KCN (0.675 g, 10.4 mmol, 6.0 equiv.) in one portion. The reaction mixture was then heated to 70 °C for 2 h. The reaction mixture changed from colourless to yellow. Then, the reaction was cooled to r.t and H_2O (5 mL) was added dropwise. The reaction mixture turned from yellow to colourless. This was then poured over EtOAc (20 mL) and the organic layer separated. The aqueous layer was extracted with EtOAc (2 x 20 mL) and the organic layers combined. They were then washed with brine (1 x 50 mL), dried over $MgSO_4$, filtered and concentrated *in vacuo* to afford a crude brown oil. This was then purified by column chromatography on silica (hexane/EtOAc 98:2) to give the title compound as a colourless oil. Yield: 0.325, (86%); $[\alpha]_D^{26} + 111$ ($c = 0.8$, $CHCl_3$); ^1H NMR (400 MHz, $CDCl_3$) δ 5.82-5.47 (m, 2H), 2.42-2.28 (m, 2H), 2.18-2.14 (m, 2H), 2.04-1.85 (m, 3H), 1.74-1.65 (m, 3H), 1.51-1.35 (m, 2H), 1.33-1.19 (m, 5H), 0.89 (t, *J* = 7.0 Hz, 3H) ^{13}C NMR (100 MHz, $CDCl_3$) δ 125.4, 124.2, 119.6, 47.3, 41.4, 37.9, 35.8, 35.7, 35.1, 30.6, 26.5, 22.8, 21.5, 17.7, 14.1; IR (neat, cm^{-1}) 3026 (w), 2919 (s), 2248 (w), 1465 (w) 1436 (w); HRMS (EI+): Exact mass calculated for $C_{15}H_{23}N$ $[M]^+$: 217.1830, found 217.1845; TLC (hexane/EtOAc 4:1, $KMnO_4$ stain): R_f = 0.80.

(1*S*,6*S*,7*R*,8*S*)-8-Butyl-7-(formylmethyl)bicyclo[4.3.0]non-3-ene (13).

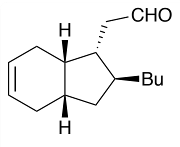

13

A stirring solution of (1*S*,6*S*,7*R*,8*S*)-8-butyl-7-(cyanomethyl)bicyclo[4.3.0]non-3-ene ***pre*-13b** (0.322 g, 1.48 mmol, 1.0 equiv.) in hexane (10 mL) was cooled to -78 °C. Then DIBAL-H (1M in hexane) (2.20 mL, 2.22 mmol, 1.5 equiv.) was added dropwise over 5 min and the reaction left to stir for 20 min. Then sat. aq. Rochelle salt (5 mL) was added dropwise to the reaction mixture and then left to warm to room temperature. The resulting cloudy suspension was poured over EtOAc (20 mL) and sat. aq. Rochelle salt (20 mL). The organic layer was separated and the aqueous phase extracted with EtOAc (2 x 20 mL). The organic phases were combined and washed with brine (1 x 50 mL), dried over $MgSO_4$, filtered and concentrated *in vacuo* to afford a crude cloudy oil. This was then purified by column chromatography on silica (hexane/EtOAc, 95:5) to afford the title compound as a colourless oil. Yield: 0.253 mg, (78%); $[\alpha]_D^{26} + 101$ ($c = 0.8$, $CHCl_3$); ^1H NMR (400 MHz, $CDCl_3$) δ 9.79 (t, *J* = 2.3 Hz, 1H), 5.68-5.51 (m, 2H), 2.49-2.44 (m, 2H), 2.35-2.23 (m, 1H), 2.22-2.12 (m, 1H), 2.10-1.98 (m, 2H), 1.92-1.78 (m, 2H), 1.74-1.60 (m, 3H), 1.47-1.35 (m, 2H), 1.35-1.15 (m, 5H), 0.88 (t, *J* = 7.0 Hz, 3H); ^{13}C NMR (100 MHz, $CDCl_3$) δ 202.9, 125.3, 124.7, 45.2, 44.7, 41.4, 37.5, 35.7, 35.6, 34.9, 30.8, 26.7, 22.9, 22.1, 14.1; IR (neat, cm^{-1})

3020 (w), 2919 (m), 2712 (w), 1723 (s); HRMS (EI+): Exact mass calculated for $C_{15}H_{24}O$ $[M]^+$: 220.1827, found 220.1824; TLC (hexane/EtOAc 4:1, $KMnO_4$ stain): R_f = 0.80.

(1*S*,6*S*,7*R*,8*S*)-8-Butyl-7-(prop-2'-yn-1'-yl)bicyclo[4.3.0]non-3-ene (14).

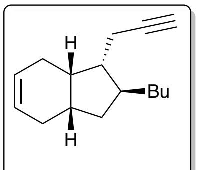

14

To a stirring solution of (1*S*,6*S*,7*R*,8*S*)-8-butyl-7-(formylmethyl)bicyclo[4.3.0]non-3-ene **13** (0.253 g, 1.15 mmol, 1.0 equiv.) in dry MeOH (6 mL) at 0 °C was added solid K_2CO_3 (0.381 mg, 2.76 mmol, 2.4 equiv.) in one portion and Ohira-Bestmann reagent (10% w/w in MeCN) (3.9 mL, 3.32 g, 1.73 mmol, 1.5 equiv.). The suspension was then warmed to room temperature and left stirring 1 h. After analysis by TLC the mixture was treated with sat. aq. $NaHCO_3$ (20 mL), and the resulting mixture poured over CH_2Cl_2 (20 mL). The organic phase was separated and the aqueous phase was washed with CH_2Cl_2 (3 x 10 mL). The organic phases were then combined, dried over Na_2SO_4, filtered and concentrated *in vacuo* to afford a crude oil. This was purified by column chromatography on silica (hexane/EtOAc, 95:5) to afford title compound as a colourless oil. Yield: 0.193 g, (78%); $[\alpha]_D^{26} + 119$ (c = 0.8, $CHCl_3$); ^1H NMR (400 MHz, $CDCl_3$) δ 5.72-5.58 (m, 2H), 2.35-2.24 (m, 2H), 2.18-2.09 (m, 3H), 2.07-1.98 (m, 1H), 1.91 (t, *J* = 2.7 Hz, 1H), 1.89-1.83 (m, 1H), 1.83-1.75 (m, 1H), 1.75-1.55 (m, 3H), 1.55-1.45 (m, 1H), 1.45-1.36 (m, 1H), 1.36-1.13 (m, 5H), 0.89 (t, *J* = 7.1 Hz, 3H); ^{13}C NMR (100 MHz, $CDCl_3$) δ; 125.2, 125.0, 84.4, 67.9, 50.2, 41.2, 37.7, 36.1, 35.9, 35.2, 30.8, 26.8, 22.9, 21.4, 18.9, 14.1; IR (neat, cm^{-1}) 3311 (m), 3020 (w), 2919 (s), 1432 (m); HRMS (EI+): Exact mass calculated for $C_{16}H_{24}$ $[M]^+$: 216.1878, found 216.1867; TLC (hexane, $KMnO_4$ stain and anisaldehyde dip): R_f = 0.25.

(1*S*,6*S*,7*R*,8*S*)-8-Butyl-7-((*E*)-7'-ethoxy-7'-oxohept-2'-enyl)bicyclo[4.3.0]non-3-ene (15).

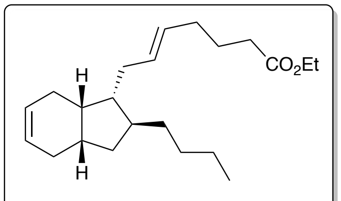

15

To a stirring solution of Cp_2ZrCl_2 (0.400 g, 1.37 mmol, 2.0 equiv.) in dry THF (8 ml) at 0 °C was added DIBAL-H (1M in hexane) (1.37 mL, 1.37 mmol, 2.0 equiv.) via dropwise addition. The resulting homogenous mixture was then protected from light and stirred at 0 °C for 1 h after which time a colourless heterogeneous mixture formed. Then (1*S*,6*S*,7*R*,8*S*)-8-butyl-7-(prop-2'-yn-1'-yl)bicyclo[4.3.0]non-3-ene **14** (0.148 g, 0.684 mmol, 1.0 equiv.) dissolved in dry THF (4 mL) was added dropwise to the reaction mixture at 0 °C. After 1 h at 0 °C iodine (0.260 g, 1.02 mmol, 1.5 equiv.) was added in one portion to the homogeneous yellow reaction mixture. The reaction mixture was then warmed to room temperature and stirred for 1 h. To the preformed vinyl iodide was successively added 4-ethoxy-4-oxobutylzinc bromide solution (0.5M in THF) (2.7 mL, 1.37 mmol, 2.0 equiv.) dropwise and $(Ph_3P)_4Pd$ (0.079 g, 0.068 mmol, 0.1 equiv.) in one portion. The resulting light brown mixture was stirred at room temperature for 1 h and monitored by TLC. Once the reaction had gone to completion 1M HCl (10 mL) was added dropwise and the reaction poured over Et_2O (15 mL). The aqueous phase was extracted with Et_2O (3 x 50 mL) and the organic phases combined, dried over $MgSO_4$, filtered and concentrated *in vacuo* to form a crude brown oily mixture. This oily mixture was purified by column chromatography on silica (hexane/EtOAc, 95:5) to afford the title compound as a colourless oil. Yield: 0.196 g, (86%); $[\alpha]_D^{26} + 67$ (c = 0.8, $CHCl_3$); ^1H NMR (400 MHz, $CDCl_3$) δ 5.69-5.56 (m, 2H), 5.48-5.33 (m, 2H), 4.12 (q, *J* = 7.1 Hz, 2 H), 2.31-2.21 (appt, *J* = 7.5 Hz, 3H), 2.16-1.82 (m, 8H), 1.75-1.50 (m, 6H), 1.48-1.41 (m, 1H), 1.39-1.22 (m, 9H), 0.88 (t, *J* = 7.1 Hz, 3H); ^{13}C NMR (100 MHz, $CDCl_3$) δ 173.8, 131.2, 129.1, 125.3, 125.1, 60.2, 51.6, 41.3, 37.3, 36.2, 35.6, 35.5, 33.7, 33.0, 31.9 31.0, 26.9, 24.8, 23.0, 21.7, 14.2, 14.1; IR (neat, cm^{-1}) 3020 (w), 2925 (m), 1734 (s); HRMS (EI+):

Exact mass calculated for $C_{22}H_{36}O_2$ $[M]^+$: 332.2715, found 332.2709; TLC (hexane/EtOAc 95:5, $KMnO_4$ stain): R_f = 0.65.

(1*S*,6*S*,7*R*,8*S*)-8-Butyl-7-((*E*)-7'-hydroxy-7'-oxohept-2'-enyl)bicyclo[4.3.0]non-3-ene (1*).

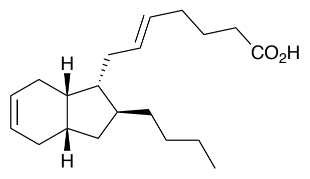

1*

To a stirring solution of the (1*S*,6*S*,7*R*,8*S*)-8-butyl-7-((*E*)-7'-ethoxy-7'-oxohept-2'-enyl)bicyclo[4.3.0]non-3-ene **15** (0.177 g, 0.533 mmol, 1.0 equiv.) in THF/MeOH/H_2O (2:2:1) (5 mL) at room temperature was added lithium hydroxide monohydrate (0.783 mg, 18.7 mmol, 35.0 equiv.) in one portion. The reaction mixture was left stirring and monitored by TLC. Left over night, the reaction had gone to completion and was acidified to pH 2 by 1M HCl (5 mL). The reaction mixture was then poured over EtOAc (5 mL) and the aqueous phase extracted with EtOAc (3 x 5 mL). The organic phases were combined and washed with brine (1 x 20 mL), dried over $MgSO_4$, filtered and concentrated *in vacuo* to provide a colourless oil. This was purified by column chromatography on silica (hexane/EtOAc, 3:2) to afford the title compound as a colourless oil. Yield: 0.154 g, (95%); $[\alpha]_D^{26} + 77$ (c = 0.8, hexane); ^{1}H NMR (400 MHz, $CDCl_3$) δ 11.63 (br, 1H), 5.67-5.56 (m, 2H), 5.50-5.34 (m, 2H), 2.34 (t, *J* = 7.5 Hz, 2H), 2.33-2.22 (m, 1H), 2.15-2.12 (m, 8H), 1.77-1.49 (m, 6H), 1.48-1.40 (m,1H), 1.40-1.08 (m, 6H), 0.88 (t, *J* = 6.7 Hz, 3H); ^{13}C NMR (100 MHz, $CDCl_3$) δ 180.3, 131.4, 128.9, 125.3, 125.1, 51.6, 41.3, 37.3, 36.2, 35.6, 35.5, 33.4, 33.0, 31.8, 31.0, 26.9, 24.5, 23.0, 21.7, 14.2; IR (neat, cm^{-1}) 3020 (w), 2925 (m), 1712 (s); HRMS (EI+): Exact mass calculated for $C_{20}H_{32}O_2$ $[M]^+$: 304.2402, found 304.2400; TLC (hexane/EtOAc 3:2, $KMnO_4$ stain): R_f = 0.40.

(1*S*,6*S*,7*R*,8*S*)-8-Butyl-7-((*E*)-7'-methoxy-7'-oxohept-2'-enyl)bicyclo[4.3.0]non-3-ene (2*).

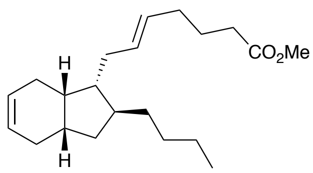

2*

To a stirring solution of (1*S*,6*S*,7*R*,8*S*)-8-butyl-7-((*E*)-7'-hydroxy-7'-oxohept-2'-enyl)bicyclo[4.3.0]non-3-ene **1*** (0.023 g, 0.076 mmol, 1.0 equiv.) in toluene/MeOH (3:2) (5 mL) at room temperature was added TMS diazomethane solution (2M in hexane) (0.06 mL, 0.113 mmol, 1.5 equiv.) dropwise over 2 min. The reaction mixture bubbled and turned transparent yellow. The reaction was monitored by TLC and after 1 h had gone to completion. The reaction mixture was then concentrated *in vacuo* and directly purified by column chromatography on silica (hexane/EtOAc, 95:5) to afford the title compound as a colourless oil. Yield: 23 mg, (96%); $[\alpha]_D^{26} + 64$ (c = 0.8, hexane); ^{1}H NMR (400 MHz, $CDCl_3$) δ 5.69-5.56 (m, 2H), 5.49-5.32 (m, 2H), 3.66 (s, 3H), 2.29 (t, *J* = 7.5 Hz, 2H), 2.26-2.21 (m, 1H), 2.13-1.82 (m, 8H), 1.75-1.63 (m, 3H), 1.63-1.50 (m, 3H), 1.40-1.08 (m, 7H), 0.88 (t, *J* = 7.1 Hz, 3H); ^{13}C NMR (100 MHz, $CDCl_3$) δ; 174.2, 131.2, 129.0, 125.3, 125.1, 51.6, 51.4, 41.3, 37.2, 36.2, 35.5, 35.4, 33.4, 33.0, 31.9, 31.0, 26.9, 24.7, 23.0, 21.7, 14.1; IR (neat, cm^{-1}) 3020 (w), 2925 (s), 1745 (s); HRMS (EI+): Exact mass calculated for $C_{21}H_{34}O_2$ $[M]^+$: 318.2559, found 318.2552; TLC (hexane/EtOAc 95:5, $KMnO_4$ stain): R_f =0.65.

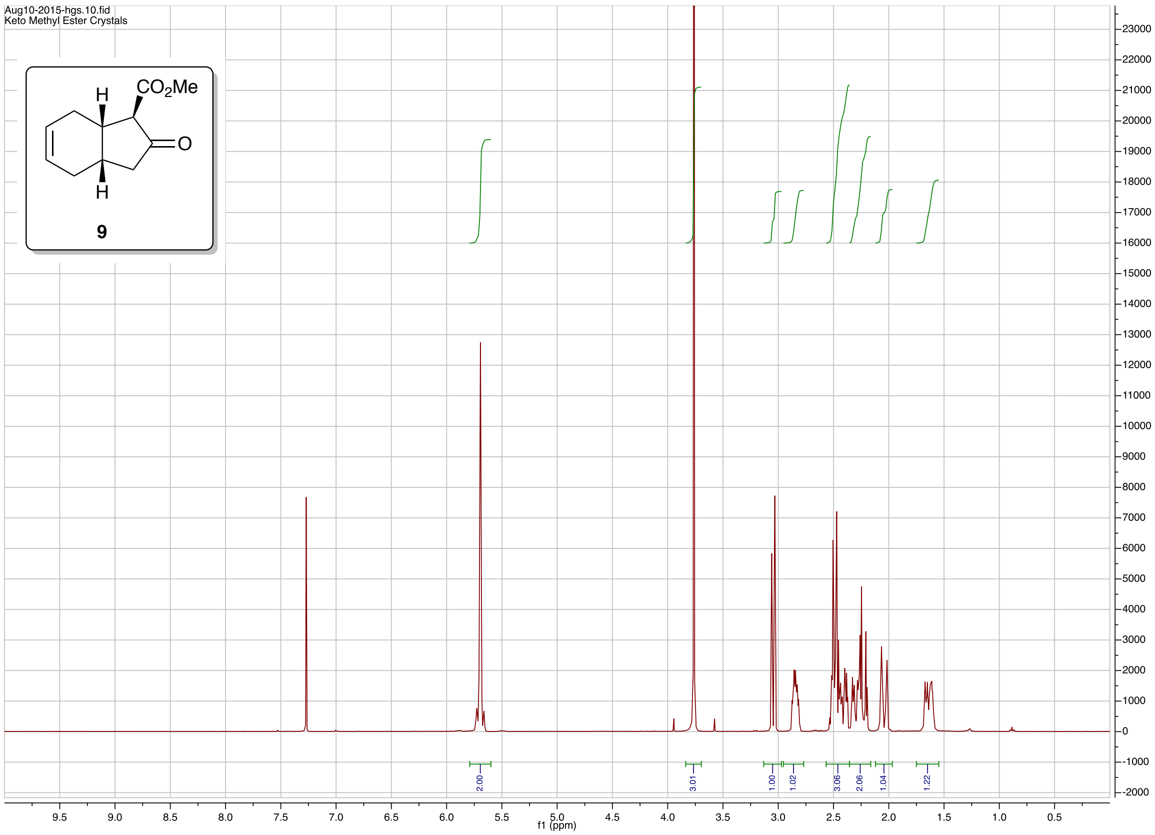

Figure S-13 ^{1}H-NMR spectrum of compound **9**.

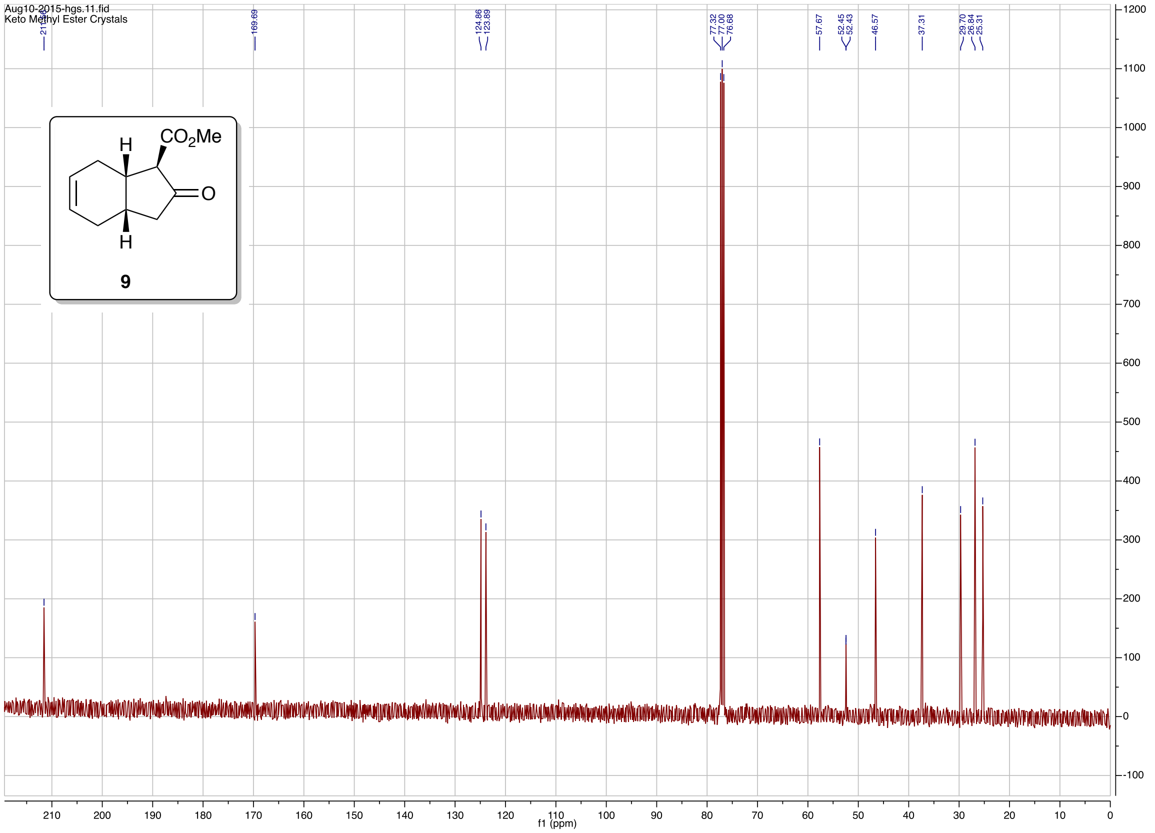

Figure S-14 ^{13}C-NMR spectrum of compound **9**.

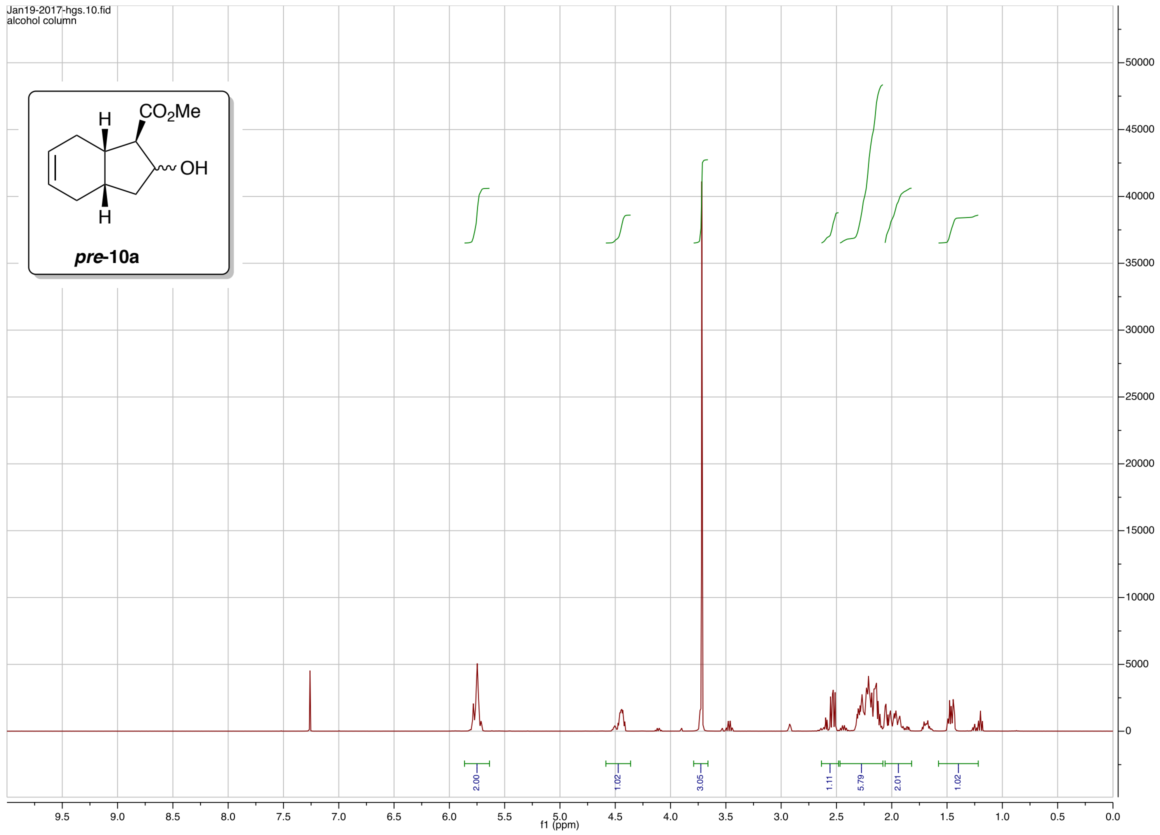

Figure S-15 ^{1}H-NMR spectrum of compound ***pre*-10a**.

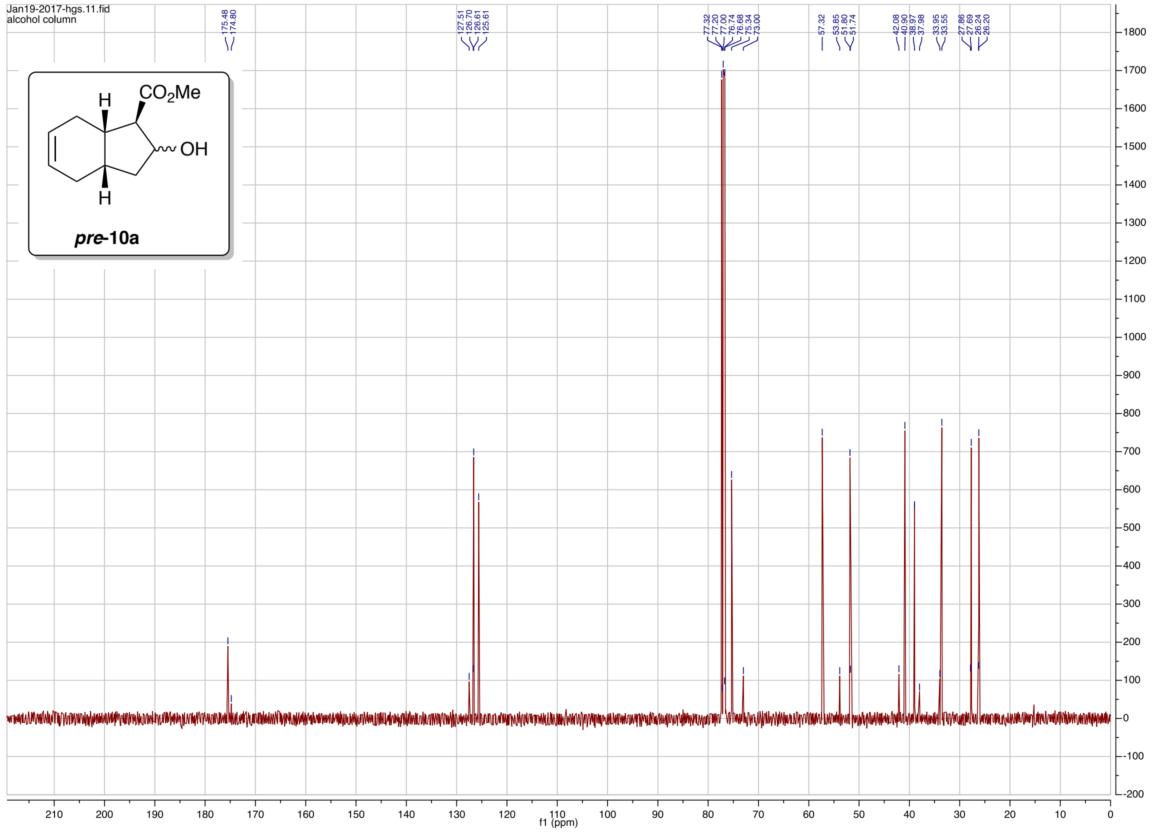

Figure S-16 ^{13}C-NMR spectrum of compound ***pre*-10a**.

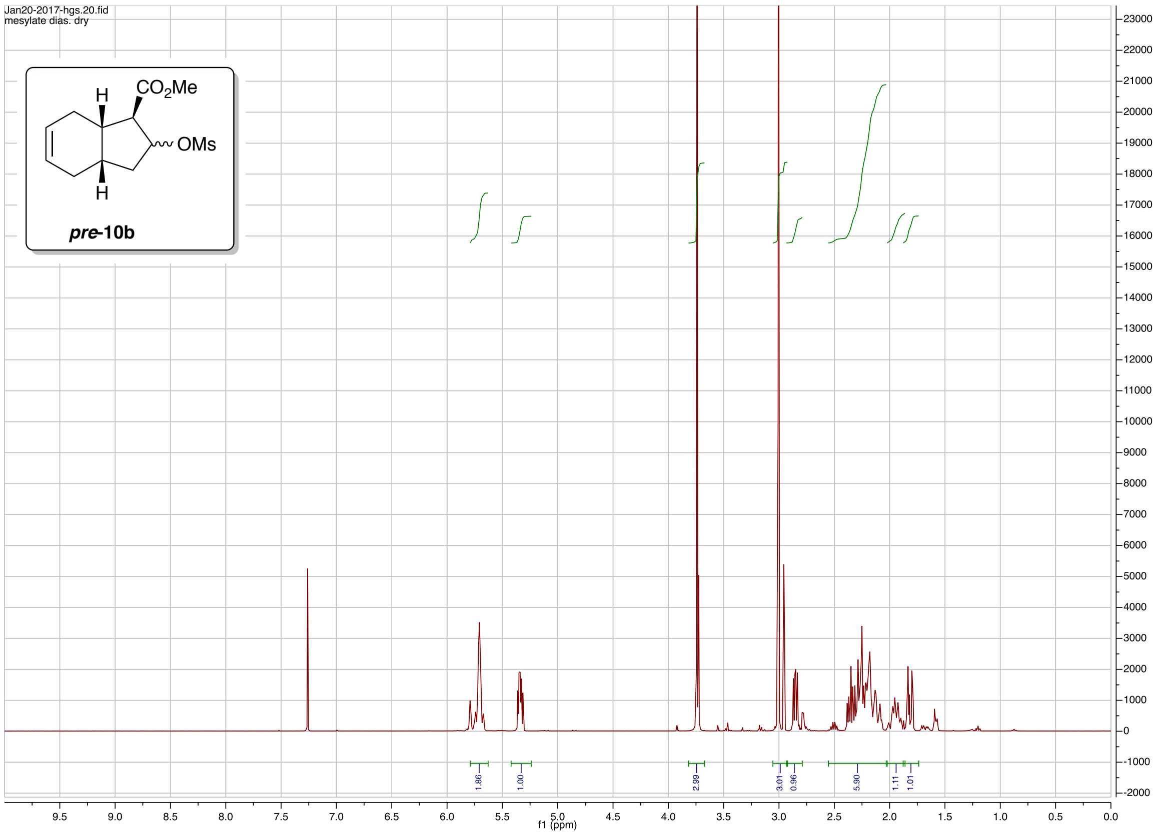

Figure S-17 ^{1}H-NMR spectrum of compound ***pre*-10b**.

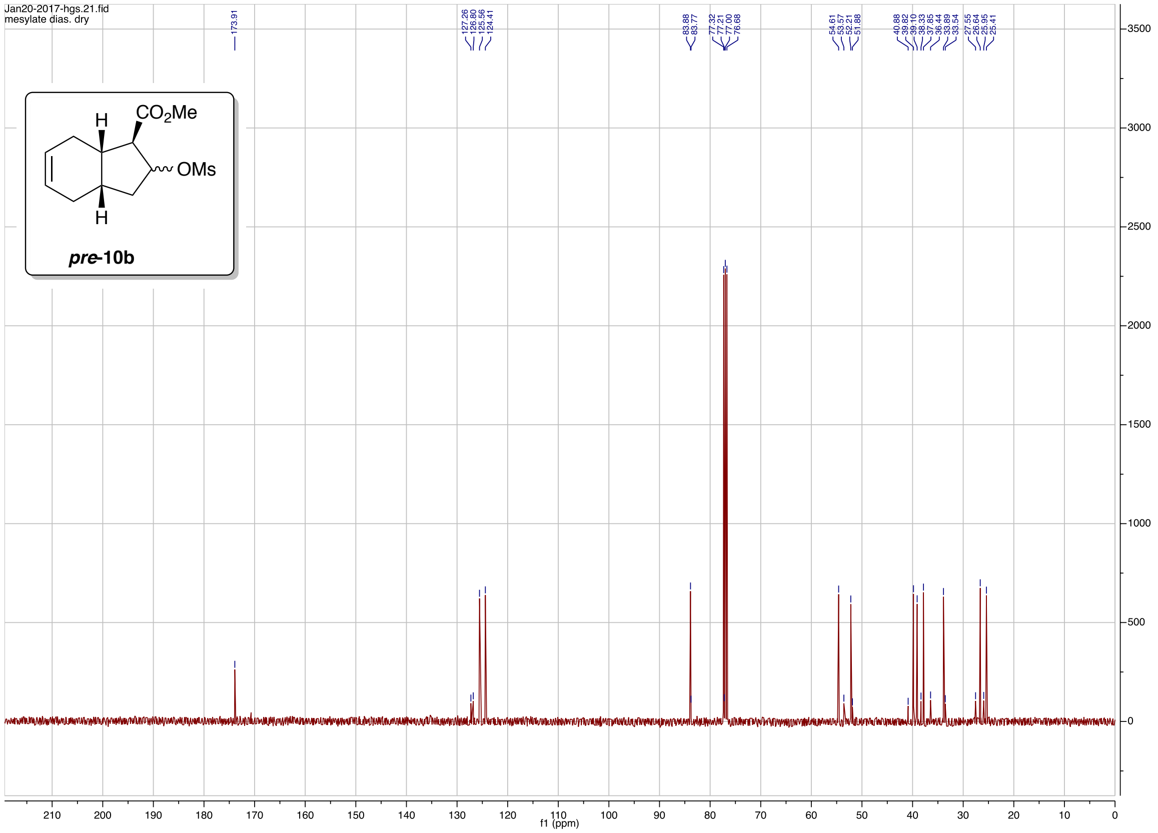

Figure S-18 ^{13}C-NMR spectrum of compound ***pre*-10b**.

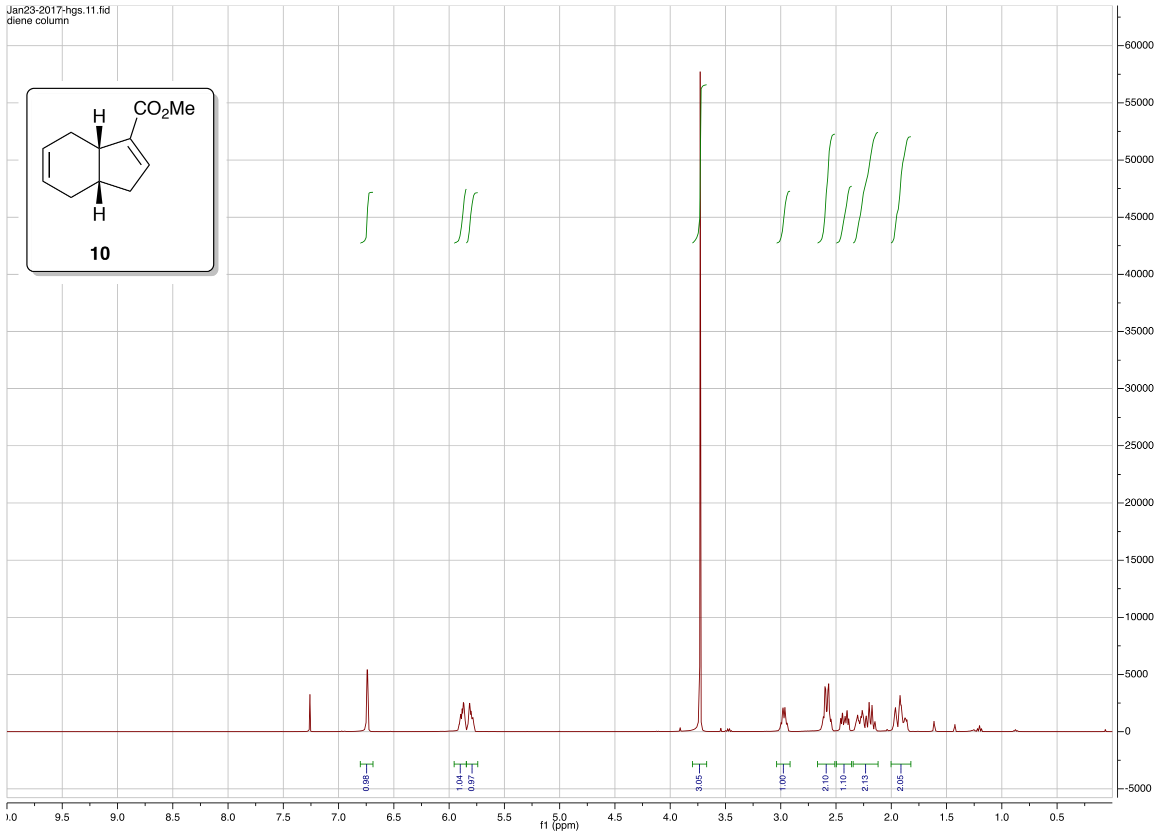

Figure S-19 ^{1}H-NMR spectrum of compound **10**.

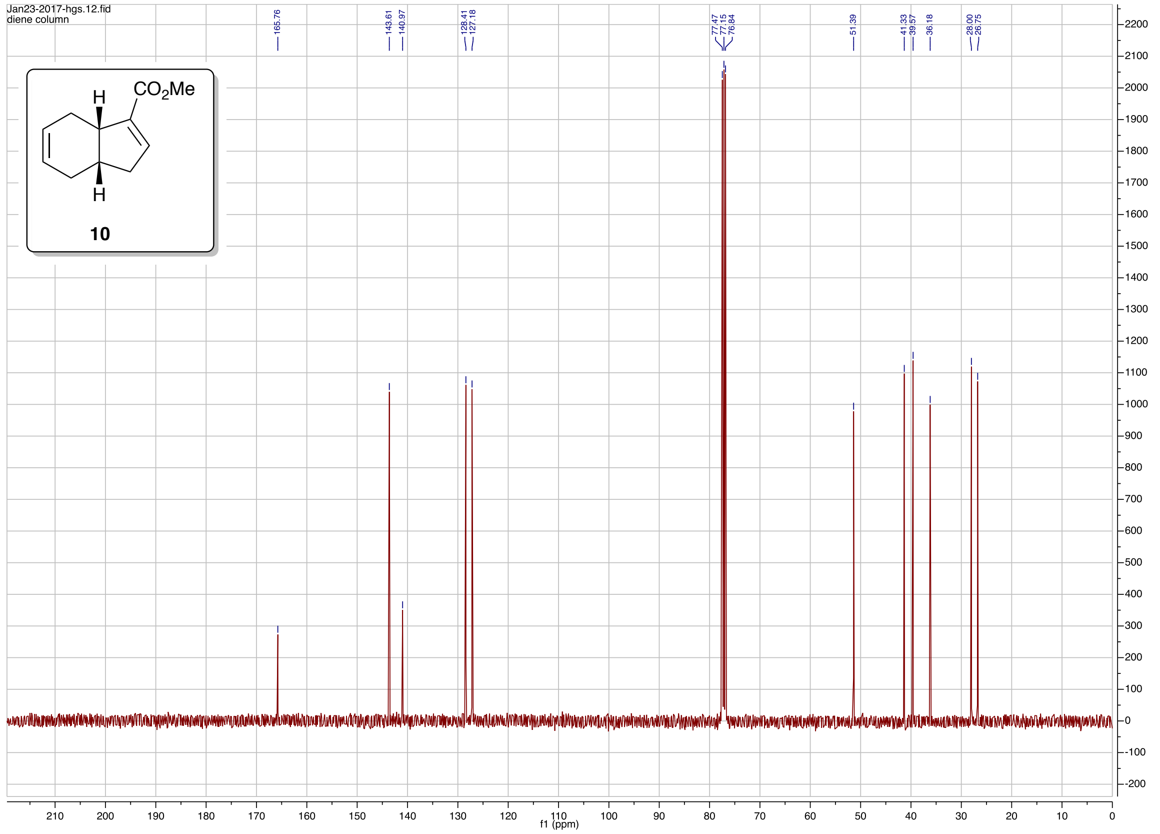

Figure S-20 ^{13}C-NMR spectrum of compound **10**.

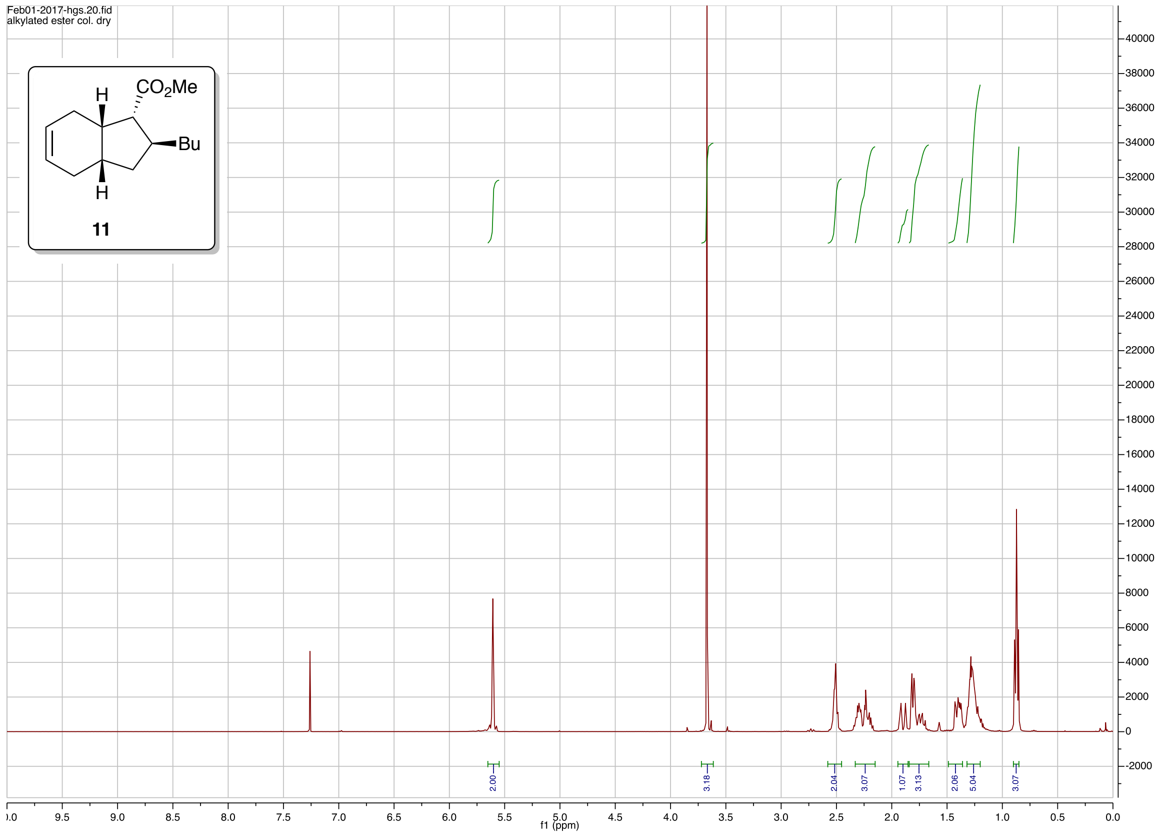

Figure S-21 ^{1}H-NMR spectrum of compound **11**.

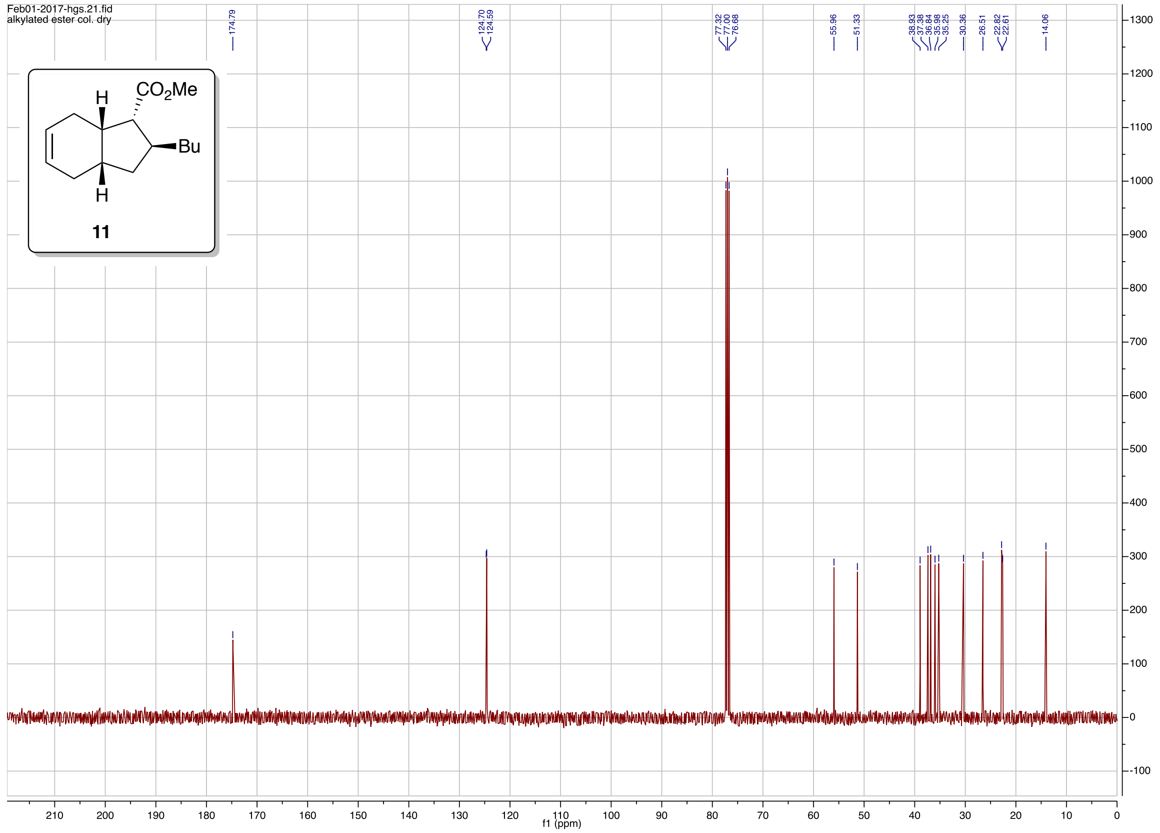

Figure S-22 ^{13}C-NMR spectrum of compound **11**.

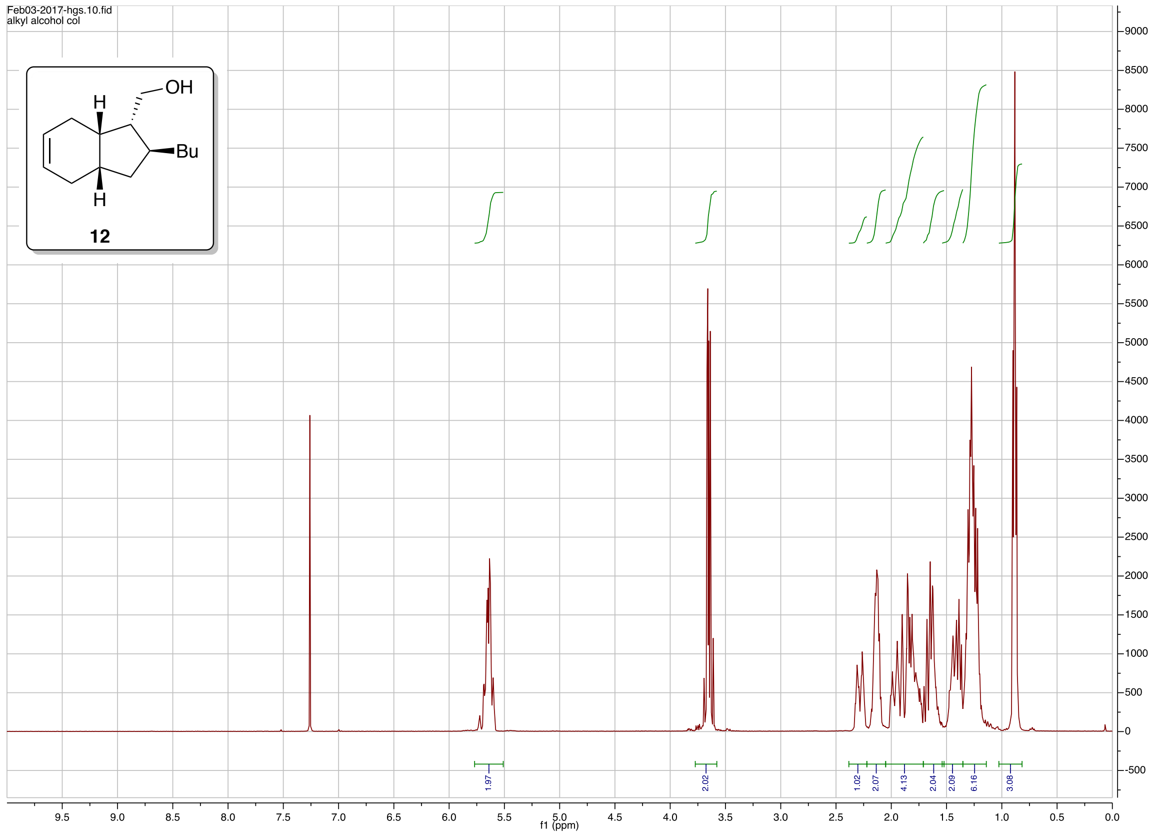

Figure S-23 ^{1}H-NMR spectrum of compound **12**.

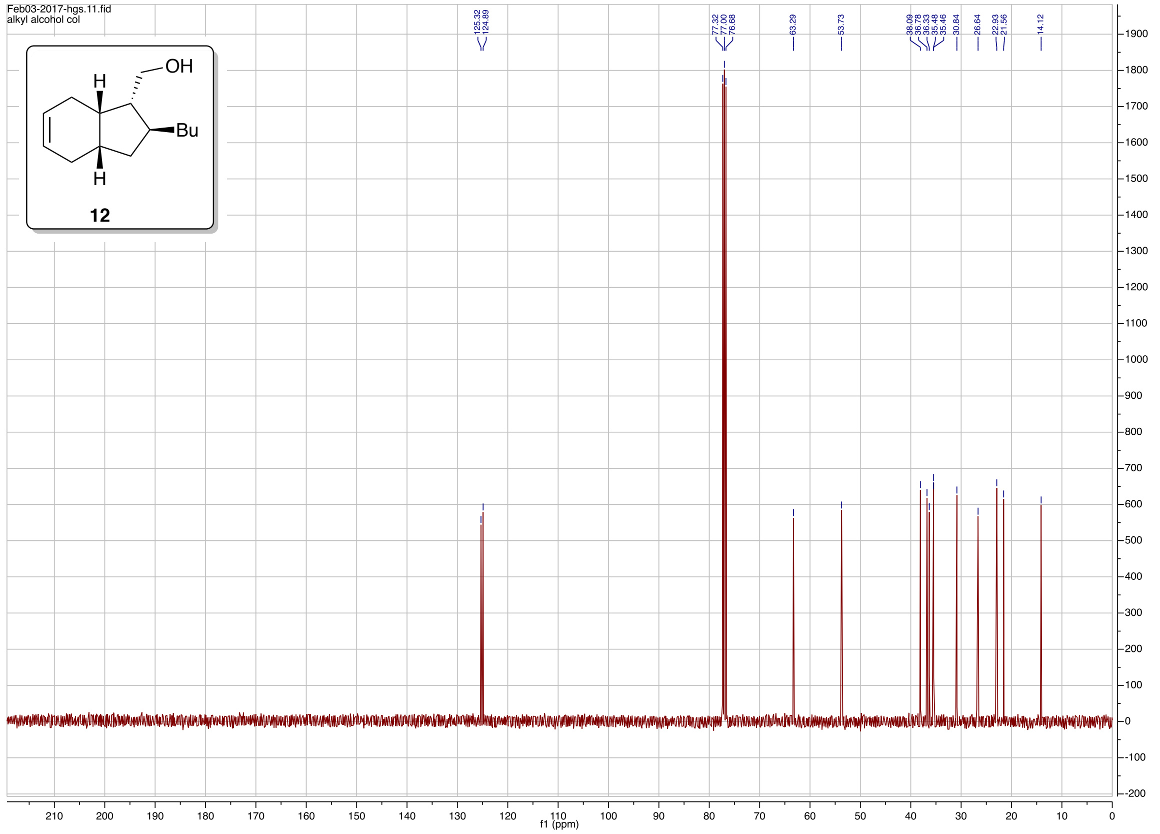

Figure S-24 ^{13}C-NMR spectrum of compound **12**.

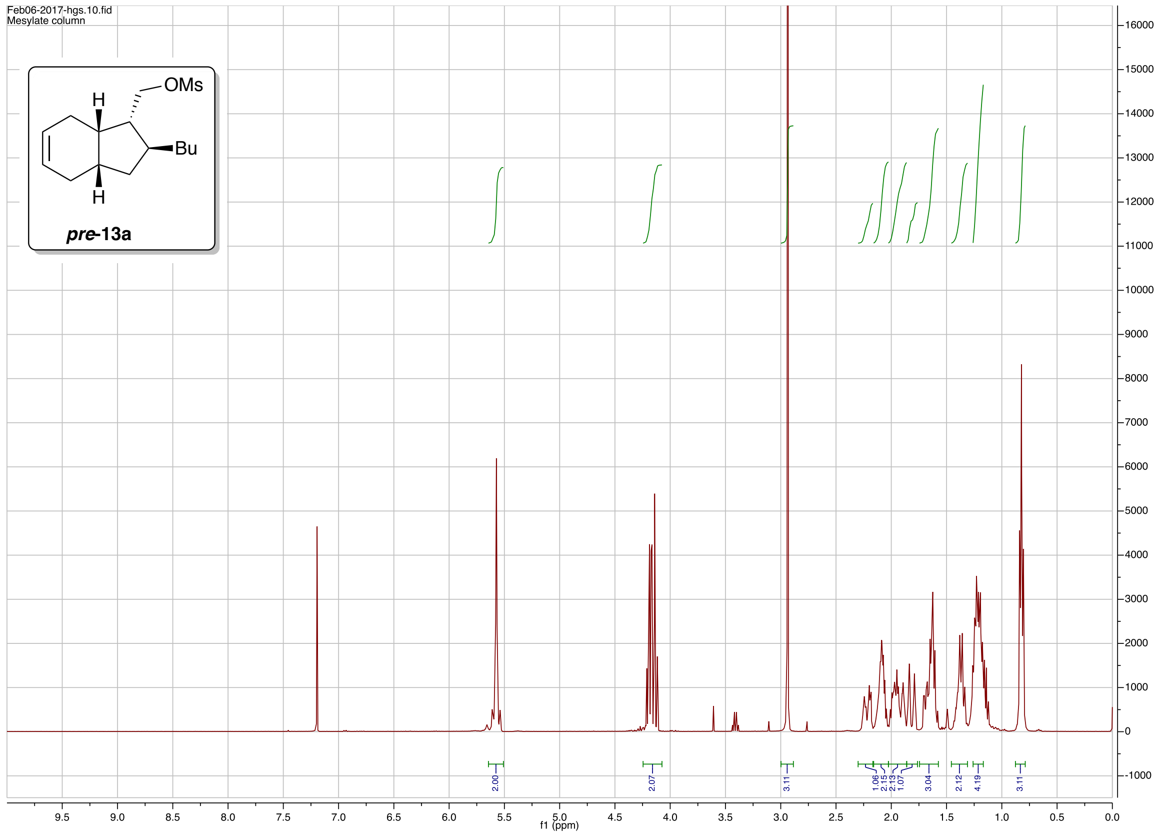

Figure S-25 ^{1}H-NMR spectrum of compound ***pre*-13a**.

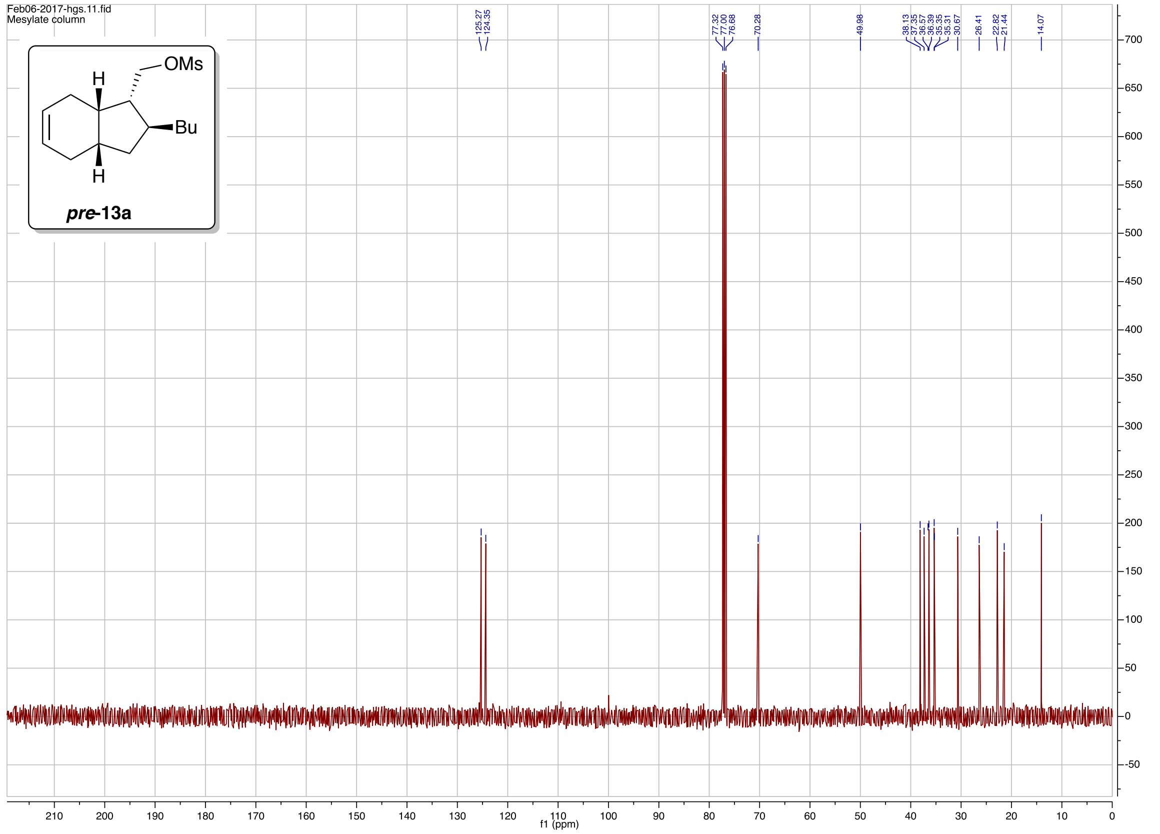

Figure S-26 ^{13}C-NMR spectrum of compound ***pre*-13a**.

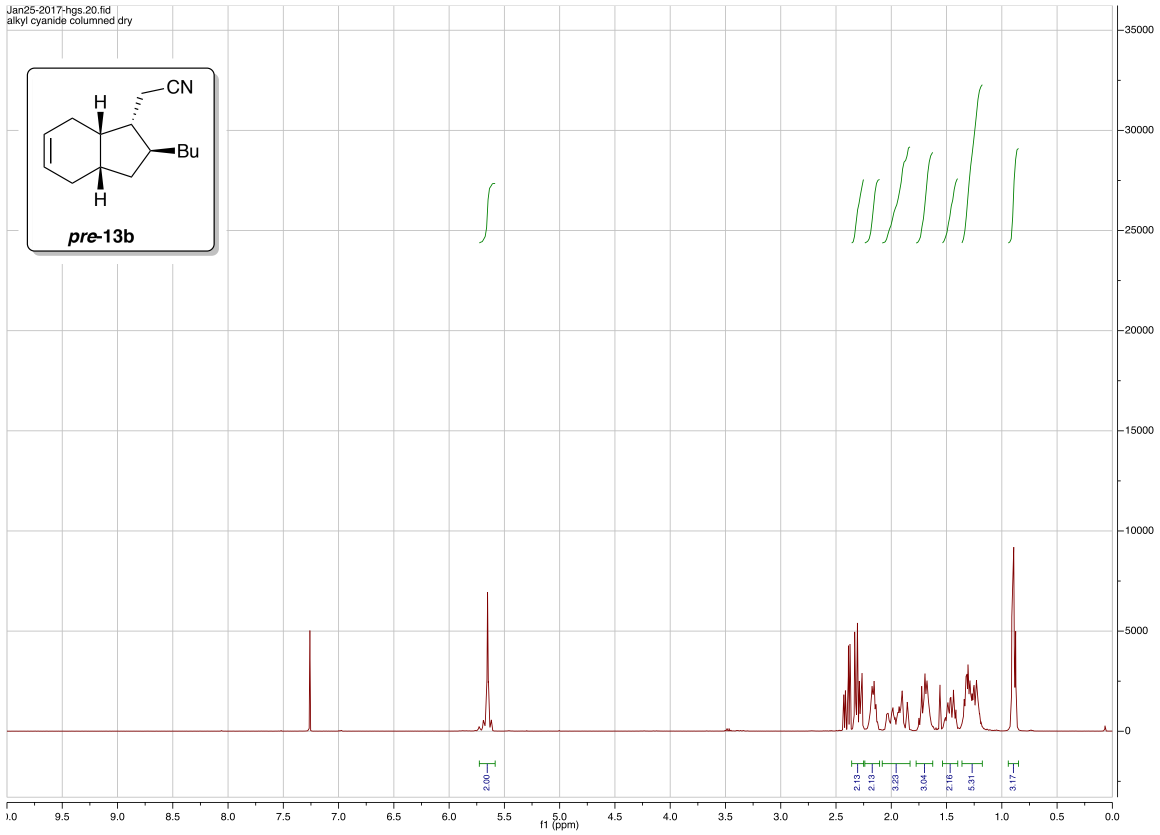

Figure S-27 ^{1}H-NMR spectrum of compound ***pre*-13b**.

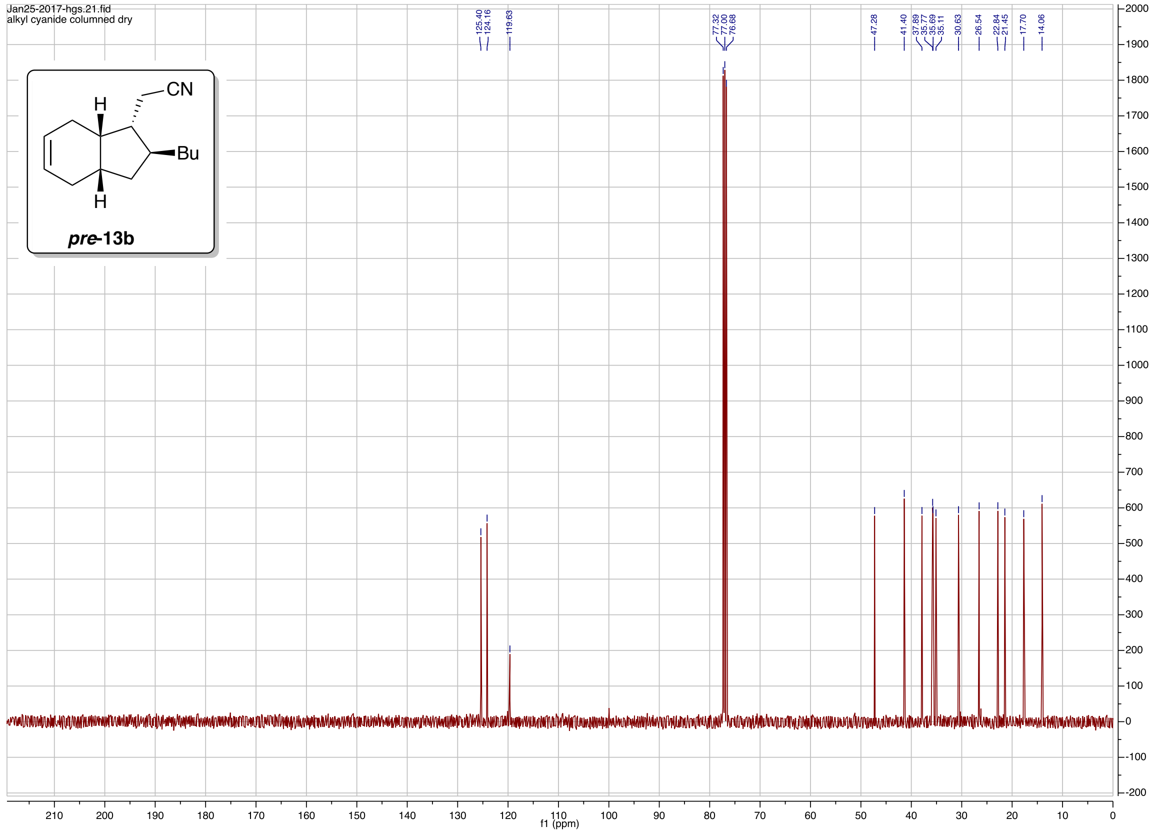

Figure S-28 ^{13}C-NMR spectrum of compound ***pre*-13b**.

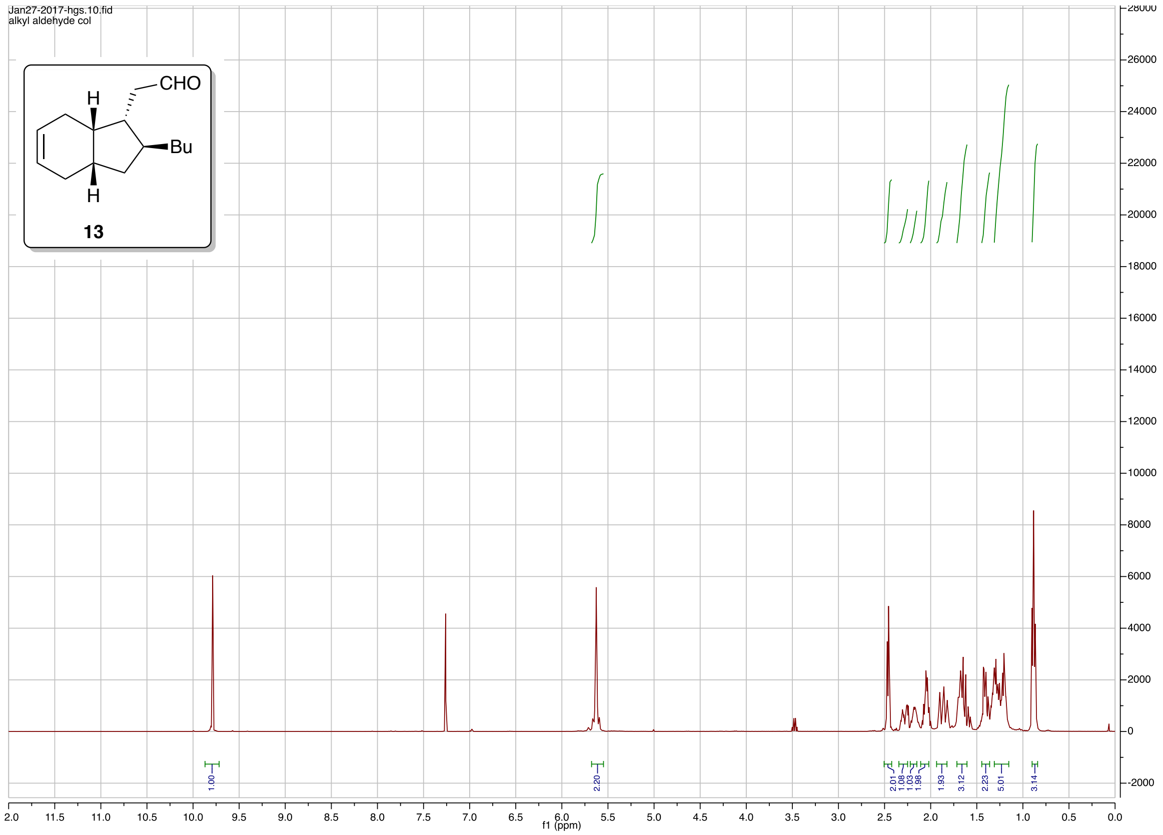

Figure S-29 ^{1}H-NMR spectrum of compound **13**.

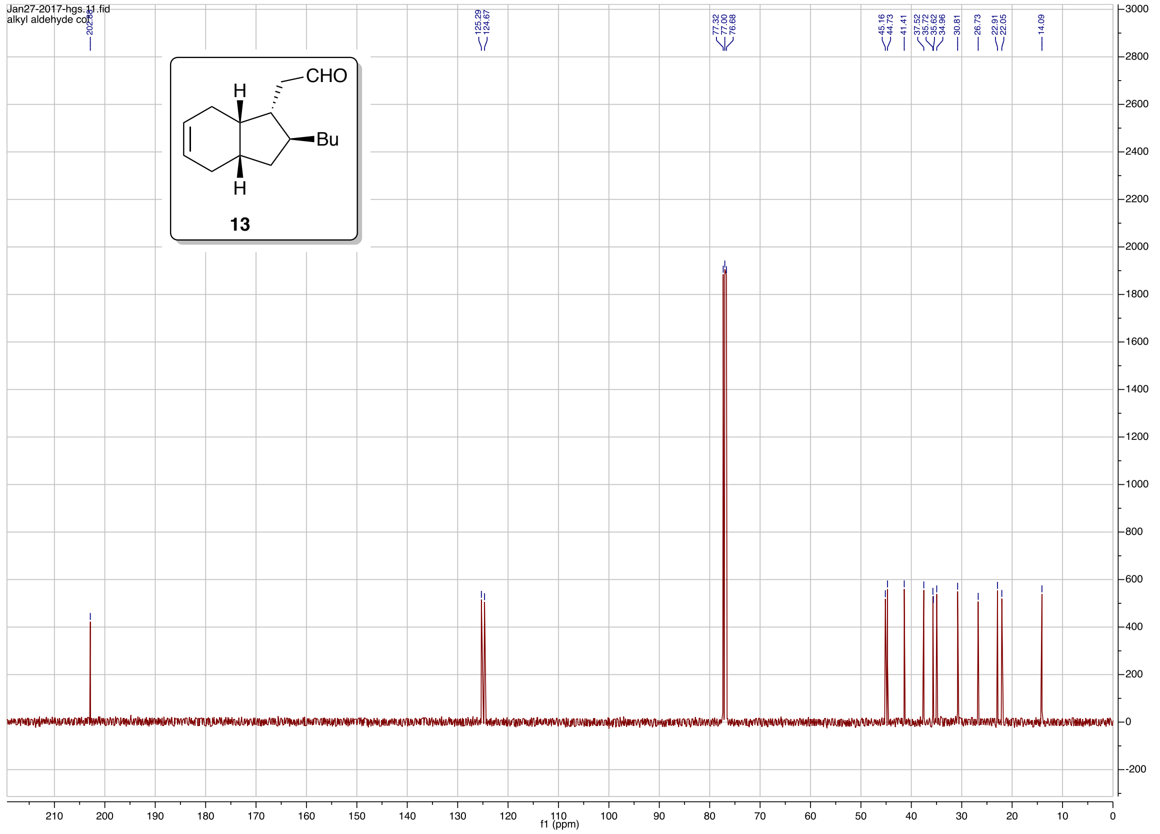

Figure S-30 ^{13}C-NMR spectrum of compound **13**.

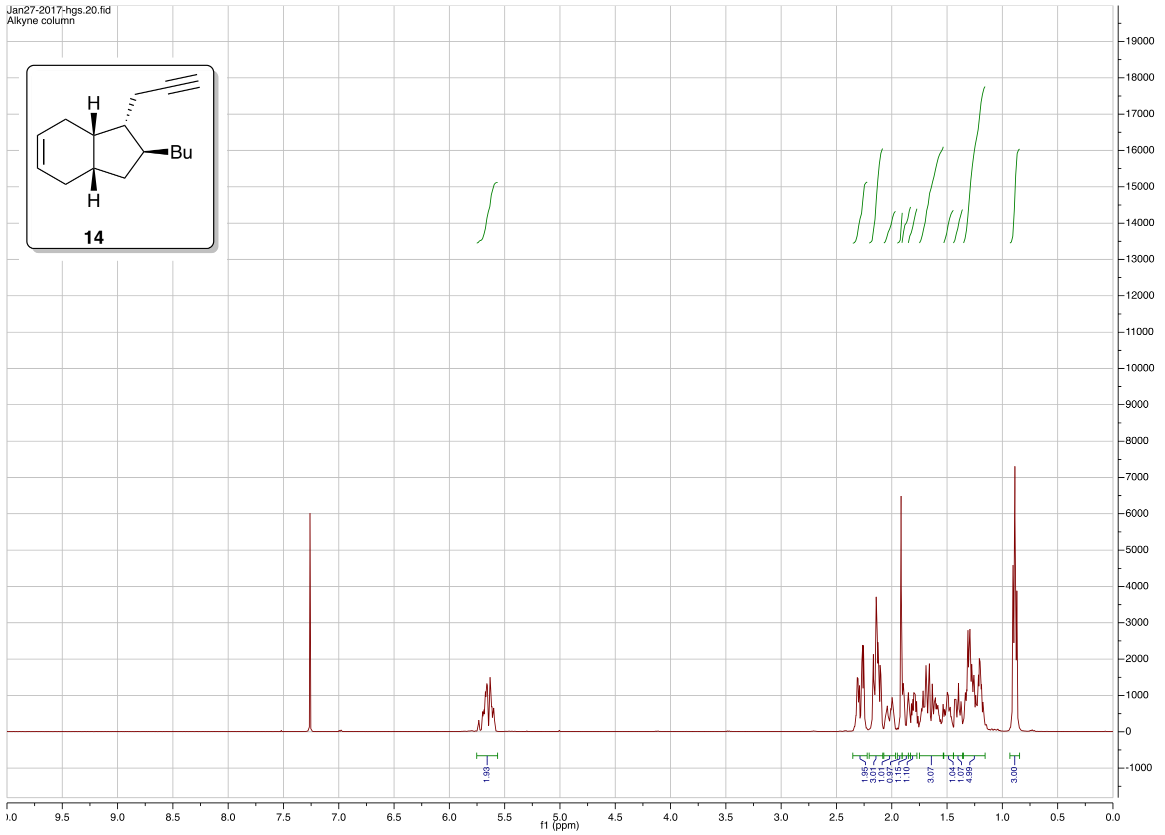

Figure S-31 ^{1}H-NMR spectrum of compound **14**.

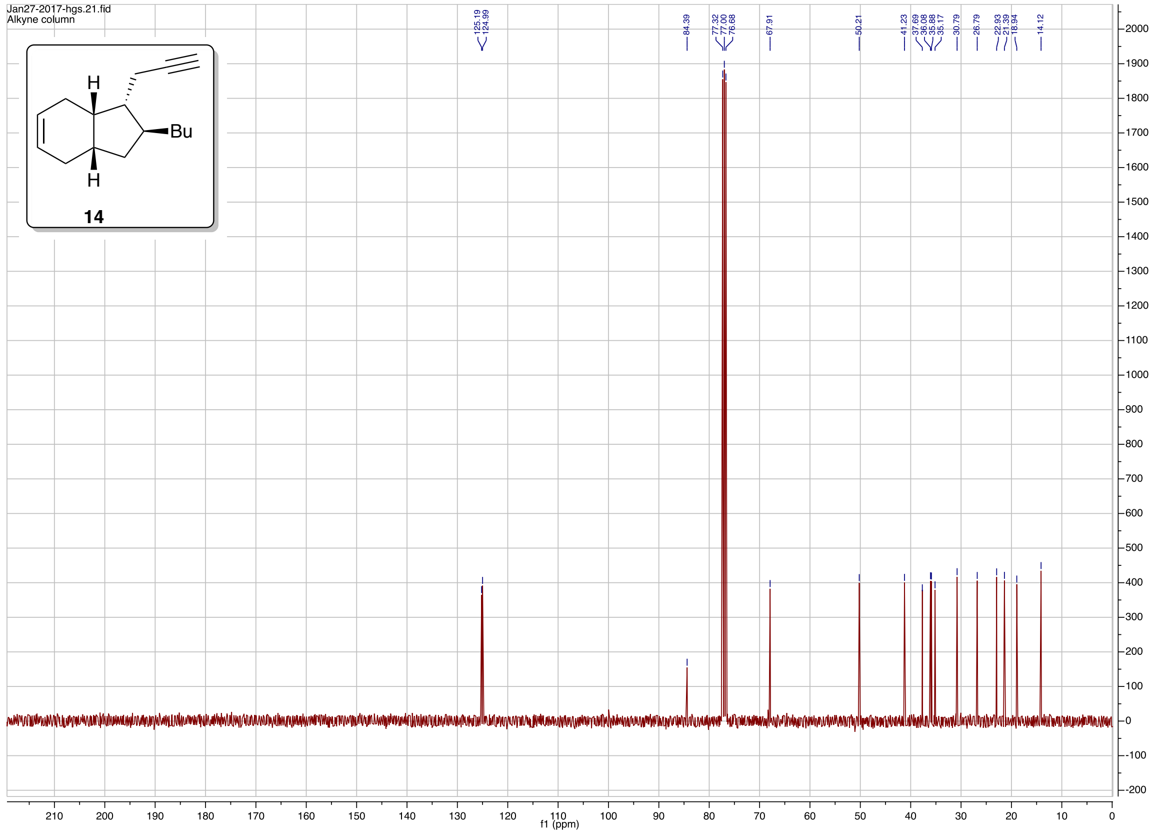

Figure S-32 ^{13}C-NMR spectrum of compound **14**.

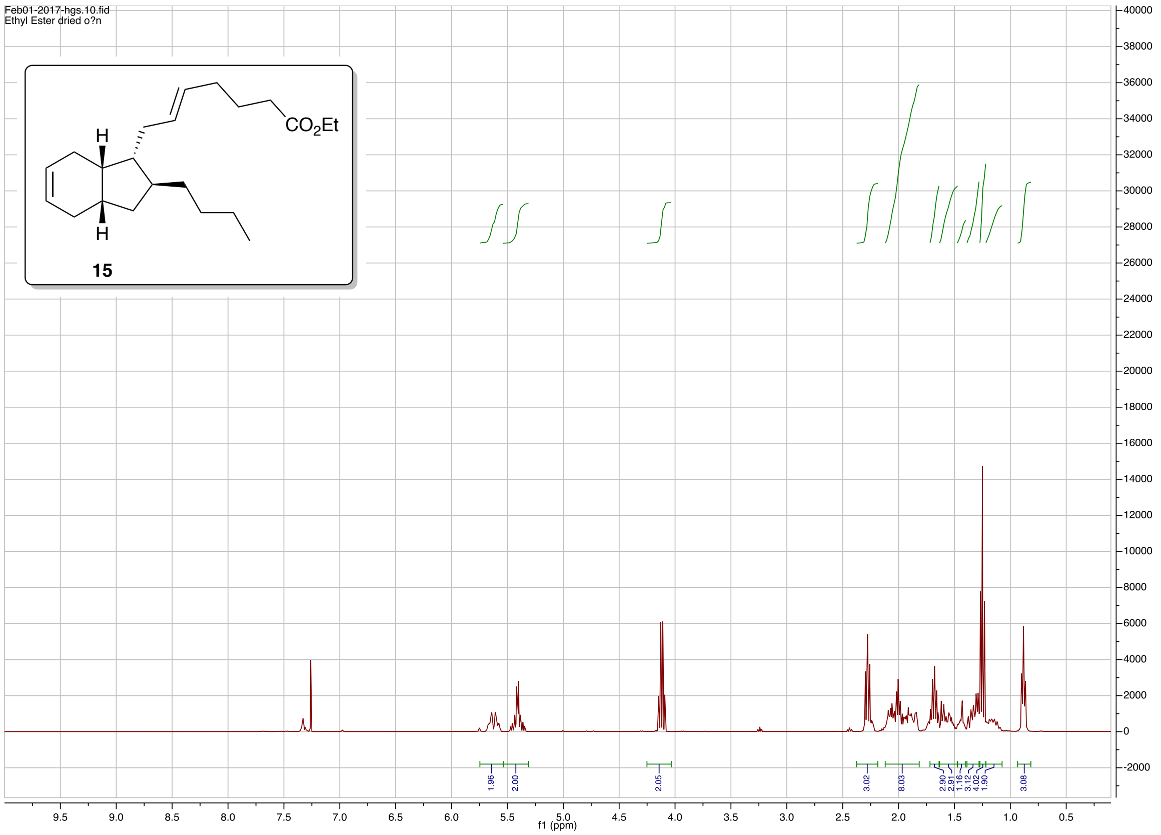

Figure S-33 ^{1}H-NMR spectrum of compound **15**.

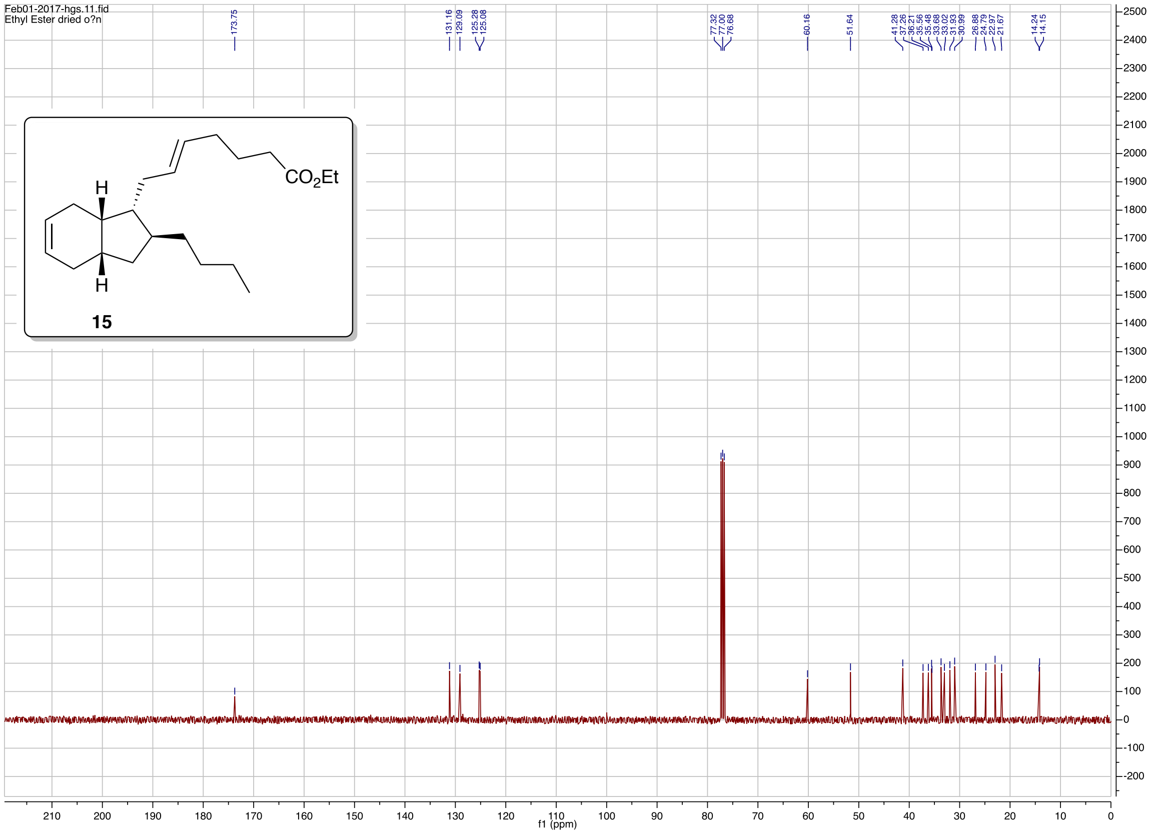

Figure S-34 ^{13}C-NMR spectrum of compound **15**.

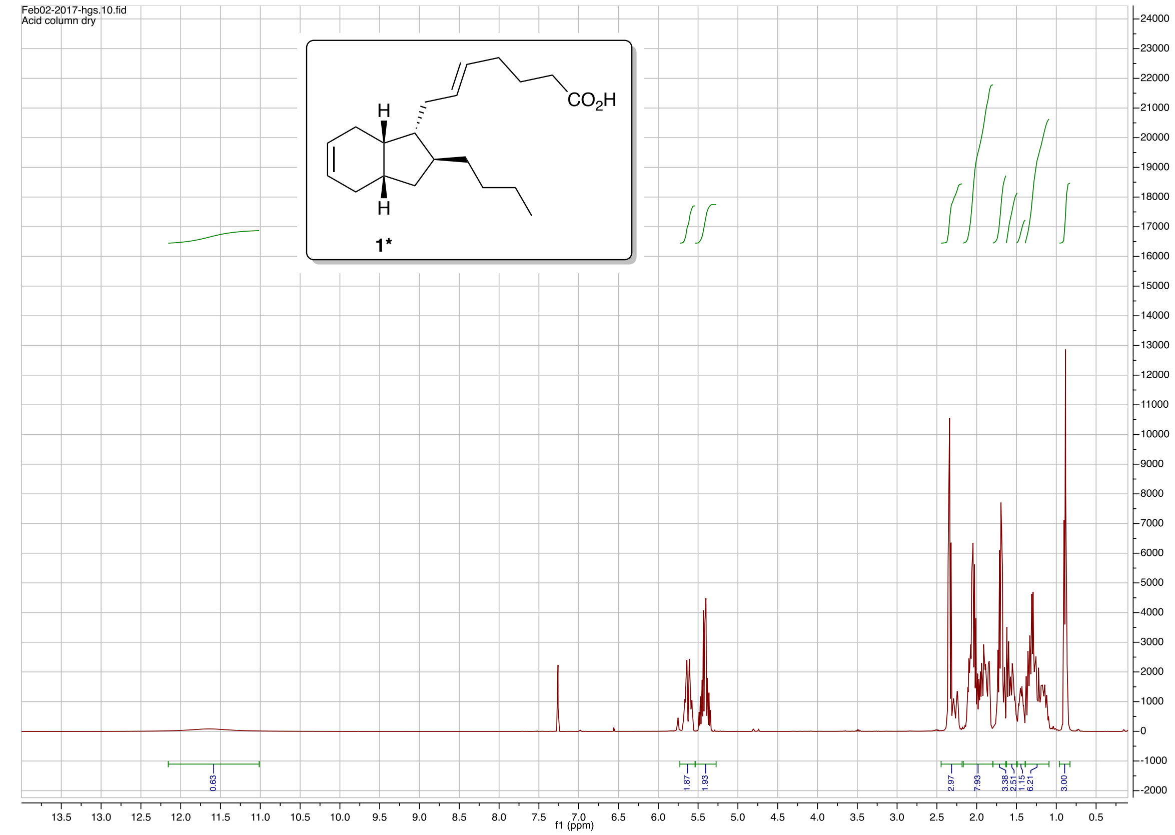

Figure S-35 ^{1}H-NMR spectrum of compound **1***.

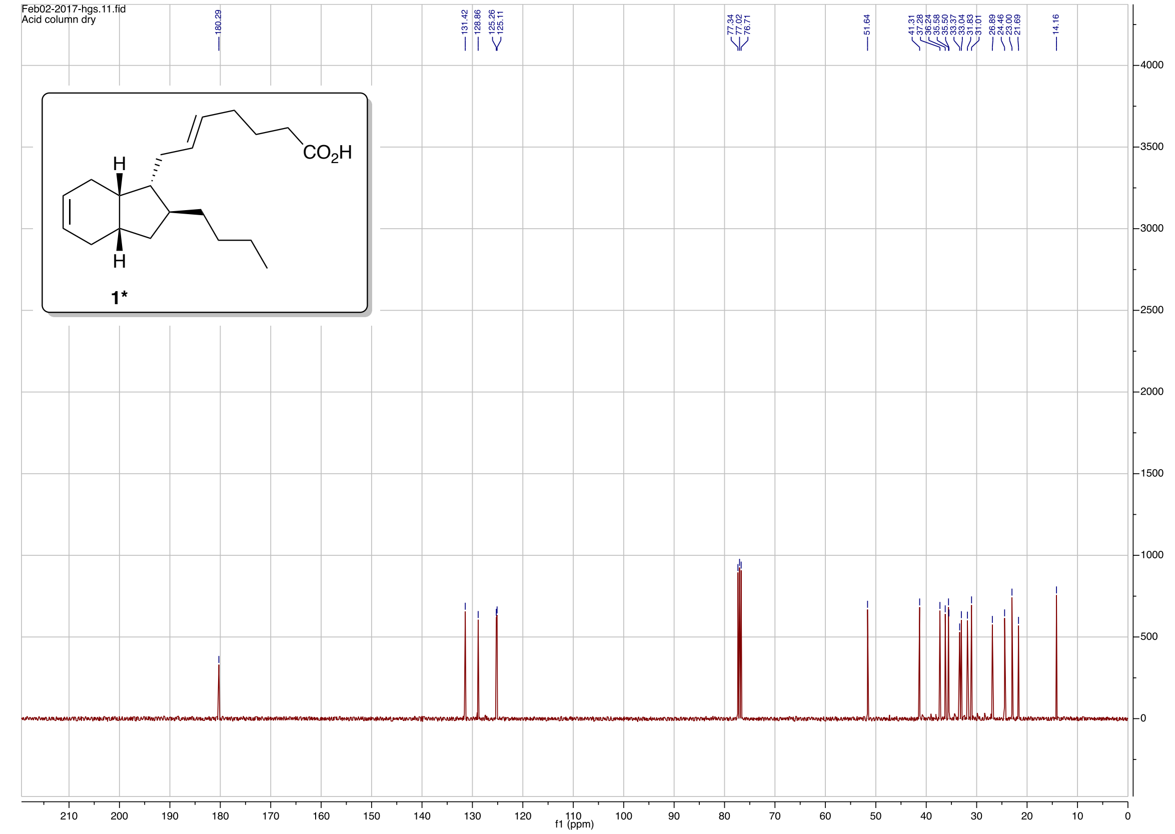

Figure S-36 ^{13}C-NMR spectrum of compound **1***.

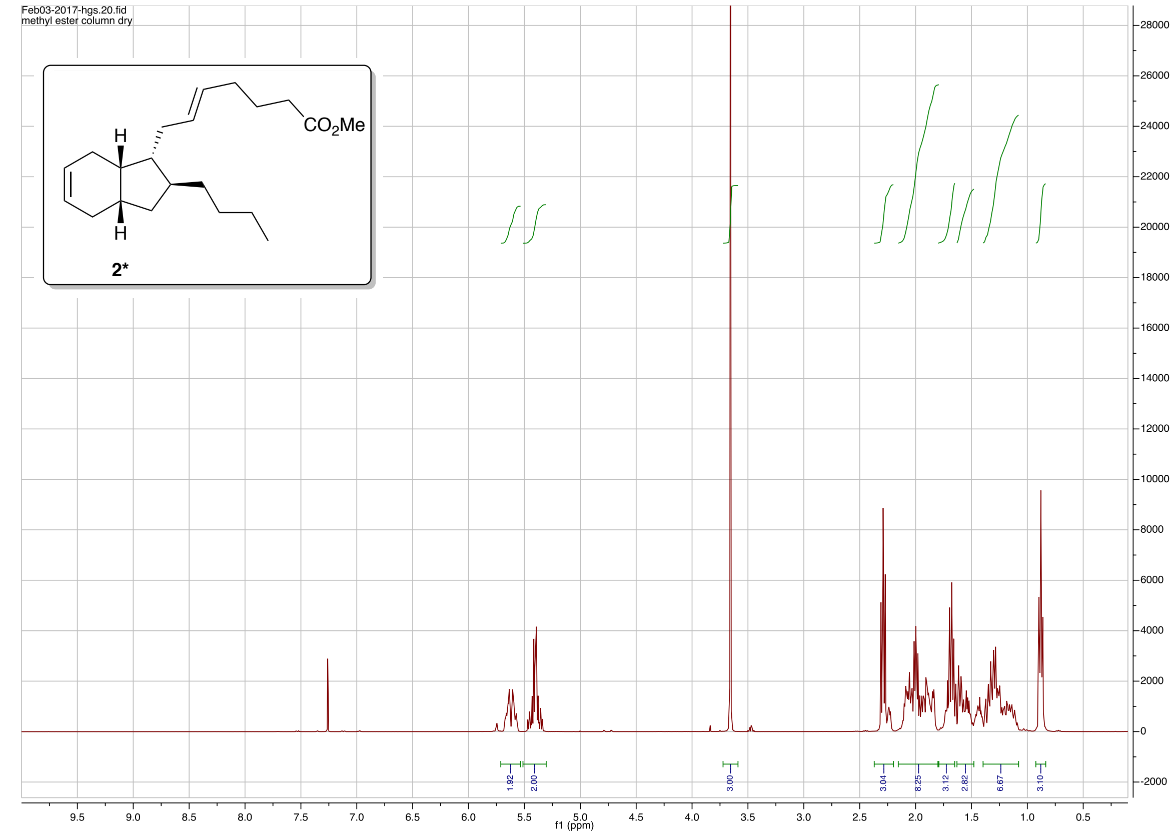

Figure S-37 ^{1}H-NMR spectrum of compound **2***.

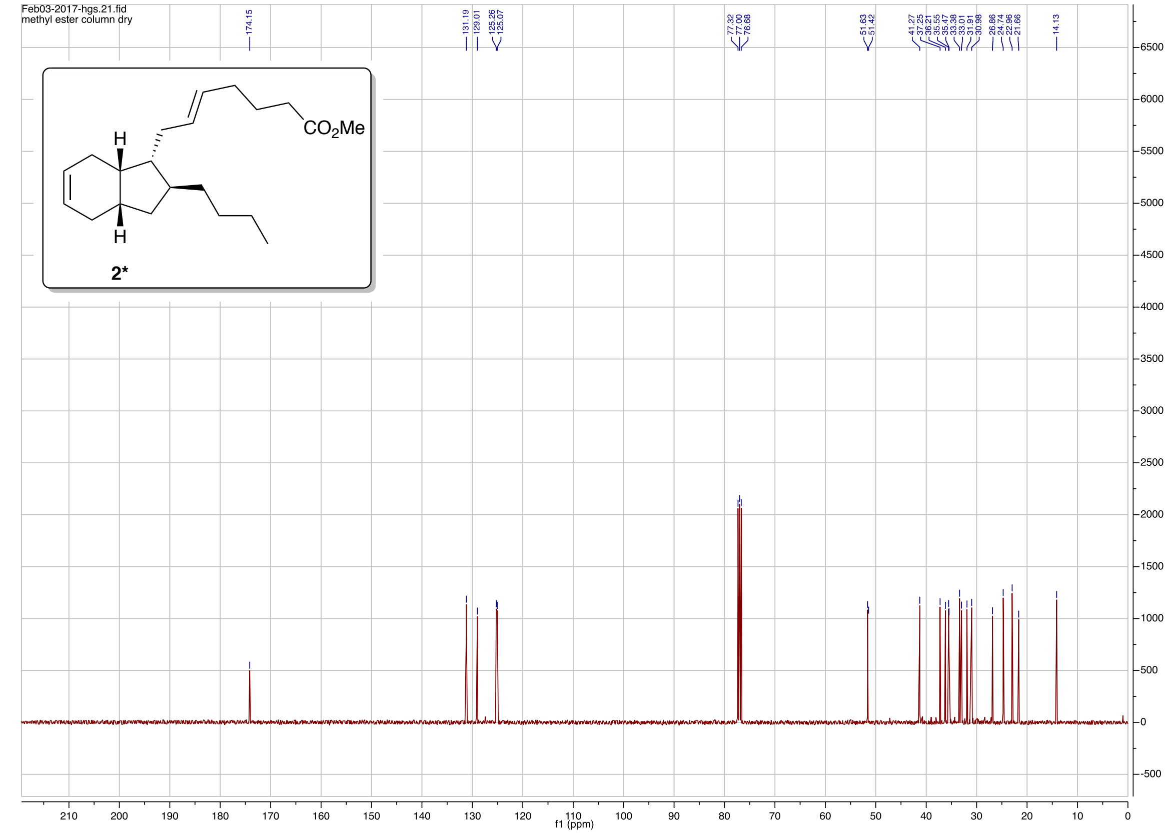

Figure S-38 ^{13}C-NMR spectrum of compound **2***.

Elemental Composition Report

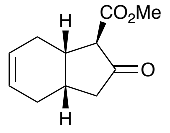

9

Single Mass Analysis

Tolerance = 10.0 PPM / DBE: min = -1.5, max = 50.0
Isotope cluster parameters: Separation = 1.0 Abundance = 1.0%

Monoisotopic Mass, Odd and Even Electron Ions
47 formula(e) evaluated with 1 results within limits (up to 50 closest results for each mass)

Sample 3 C11H14O3 MW 194
DEHGS2016021903 65 (2.723)

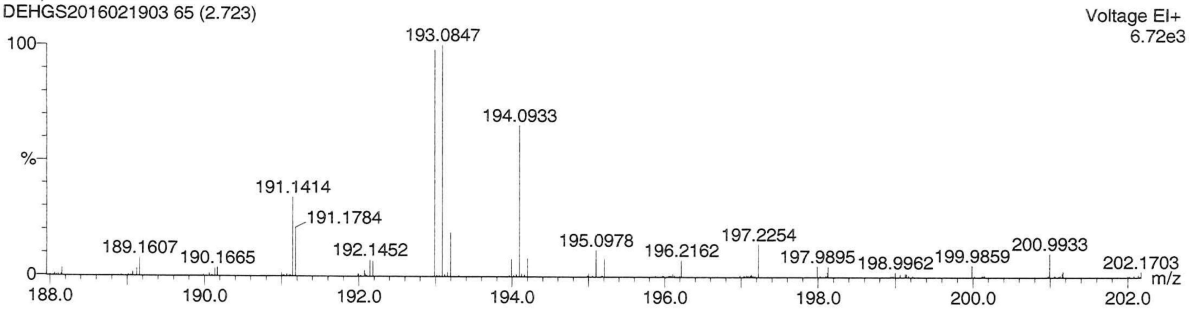

Minimum:				-1.5		
Maximum:		200.0	10.0	50.0		
Mass	Calc. Mass	mDa	PPM	DBE	Score	Formula
194.0933	194.0943	-1.0	-5.1	5.0	1	C11 H14 O3

Figure S-39 HRMS of compound **9**.

Elemental Composition Report

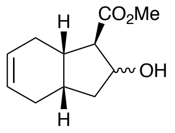

***pre*-10a**

Single Mass Analysis

Tolerance = 10.0 PPM / DBE: min = -1.5, max = 50.0
Isotope cluster parameters: Separation = 1.0 Abundance = 1.0%

Monoisotopic Mass, Odd and Even Electron Ions
18 formula(e) evaluated with 1 results within limits (up to 50 closest results for each mass)

1 C11H16O3
dehgs2017300101 325 (6.566)

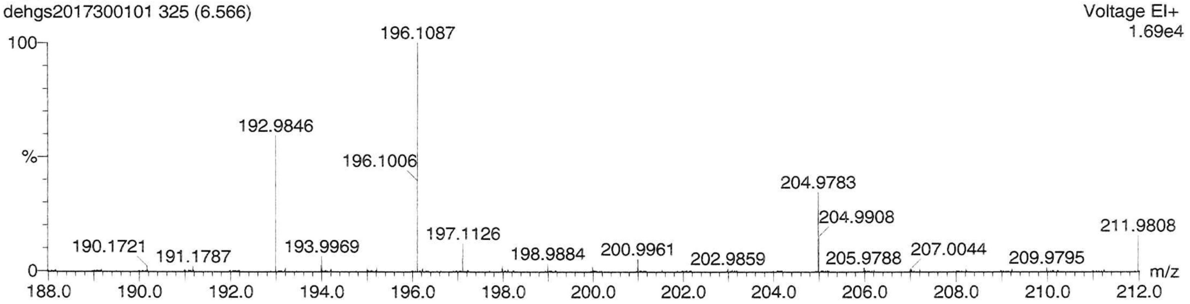

Minimum:				-1.5		
Maximum:		200.0	10.0	50.0		
Mass	Calc. Mass	mDa	PPM	DBE	Score	Formula
196.1087	196.1099	-1.2	-6.3	4.0	1	C11 H16 O3

Figure S-40 HRMS of compound ***pre*-10a**.

Elemental Composition Report

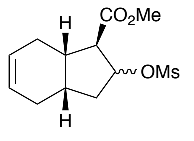

pre-10b

Page 1

Single Mass Analysis

Tolerance = 10.0 PPM / DBE: min = -1.5, max = 50.0
Isotope cluster parameters: Separation = 1.0 Abundance = 1.0%

Monoisotopic Mass, Odd and Even Electron Ions
31 formula(e) evaluated with 1 results within limits (up to 50 closest results for each mass)

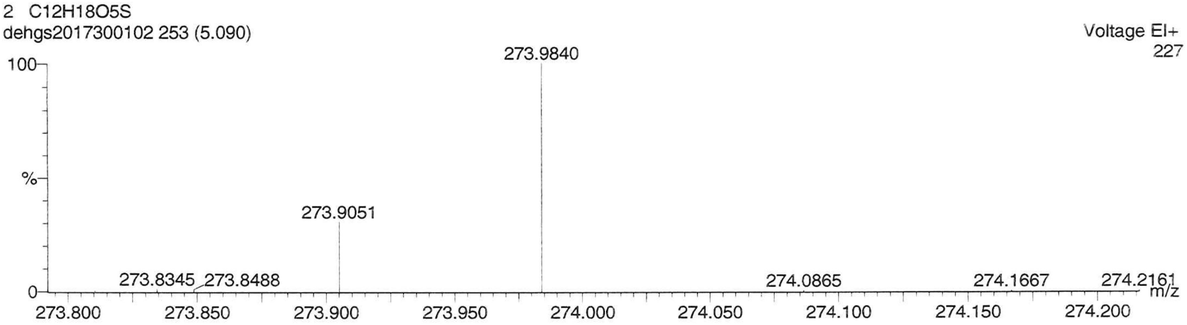

Minimum:				-1.5		
Maximum:		200.0	10.0	50.0		
Mass	Calc. Mass	mDa	PPM	DBE	Score	Formula
274.0865	274.0875	-1.0	-3.6	4.0	1	C12 H18 O5 S

Figure S-41 HRMS of compound ***pre*-10b**.

Elemental Composition Report

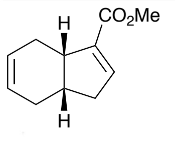

10

Page 1

Single Mass Analysis

Tolerance = 10.0 PPM / DBE: min = -1.5, max = 50.0
Isotope cluster parameters: Separation = 1.0 Abundance = 1.0%

Monoisotopic Mass, Odd and Even Electron Ions
21 formula(e) evaluated with 1 results within limits (up to 50 closest results for each mass)

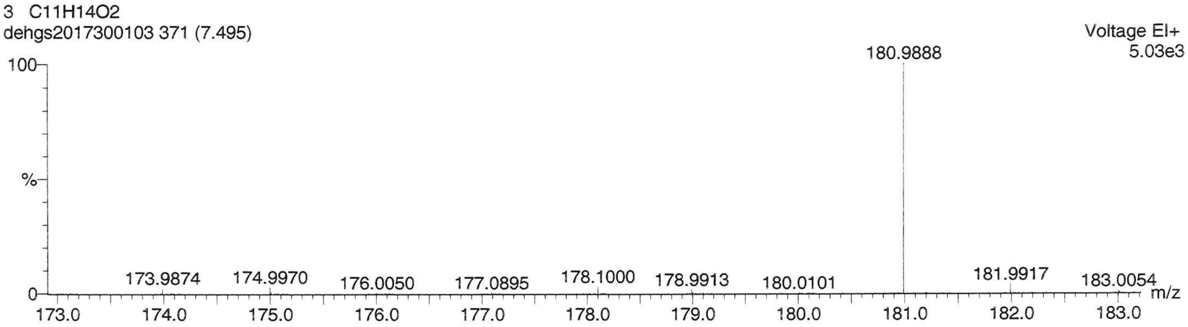

Minimum:				-1.5		
Maximum:		200.0	10.0	50.0		
Mass	Calc. Mass	mDa	PPM	DBE	Score	Formula
178.1000	178.0994	0.6	3.5	5.0	1	C11 H14 O2

Figure S-42 HRMS of compound **10**.

Elemental Composition Report

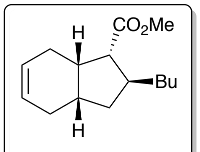

11

Page 1

Single Mass Analysis
Tolerance = 10.0 PPM / DBE: min = -1.5, max = 50.0
Isotope cluster parameters: Separation = 1.0 Abundance = 1.0%

Monoisotopic Mass, Odd and Even Electron Ions
49 formula(e) evaluated with 1 results within limits (up to 50 closest results for each mass)

C15H24O2
DEHGS2017020902 60 (2.209)

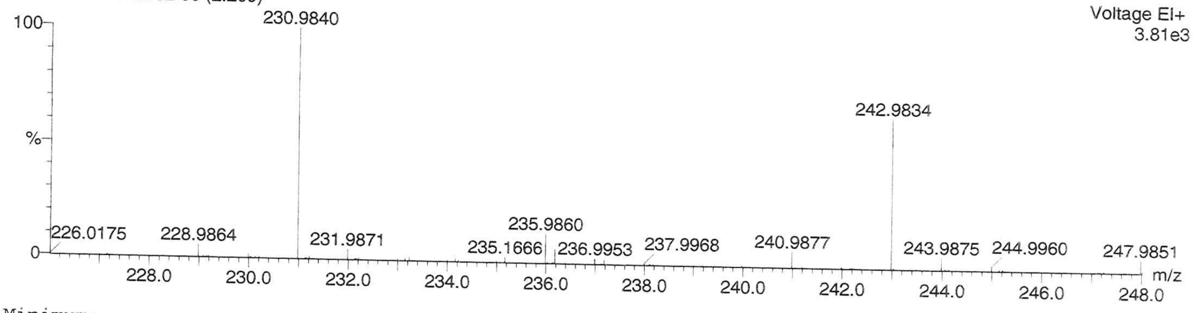

Minimum:				-1.5		
Maximum:		200.0	10.0	50.0		
Mass	Calc. Mass	mDa	PPM	DBE	Score	Formula
236.1763	236.1776	-1.3	-5.6	4.0	1	C15 H24 O2

Figure S-43 HRMS of compound **11**.

Elemental Composition Report

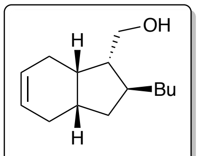

12

Page 1

Single Mass Analysis
Tolerance = 10.0 PPM / DBE: min = -1.5, max = 50.0
Isotope cluster parameters: Separation = 1.0 Abundance = 1.0%

Monoisotopic Mass, Odd and Even Electron Ions
80 formula(e) evaluated with 1 results within limits (up to 50 closest results for each mass)

C14H24O MW 208
DEHGS2017020904 136 (5.009)

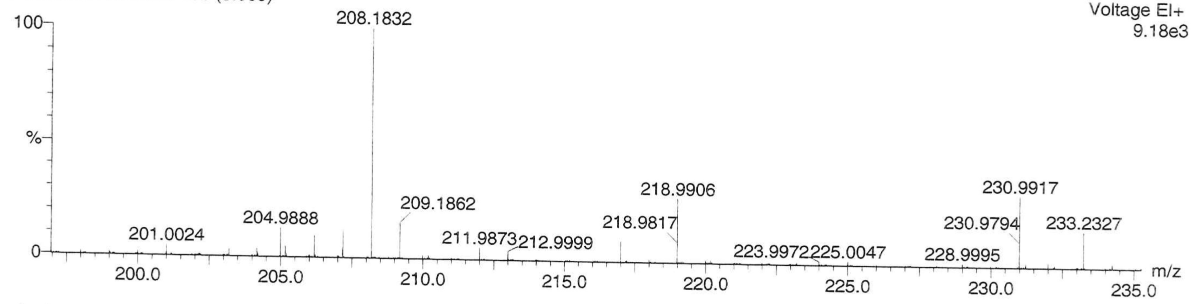

Minimum:				-1.5		
Maximum:		200.0	10.0	50.0		
Mass	Calc. Mass	mDa	PPM	DBE	Score	Formula
208.1832	208.1827	0.5	2.3	3.0	1	C14 H24 O

Figure S-44 HRMS of compound **12**.

Elemental Composition Report

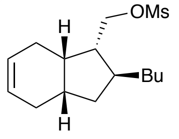

pre-**13a**

Page 1

Single Mass Analysis

Tolerance = 10.0 PPM / DBE: min = -1.5, max = 50.0
Isotope cluster parameters: Separation = 1.0 Abundance = 1.0%

Monoisotopic Mass, Odd and Even Electron Ions
112 formula(e) evaluated with 2 results within limits (up to 50 closest results for each mass)

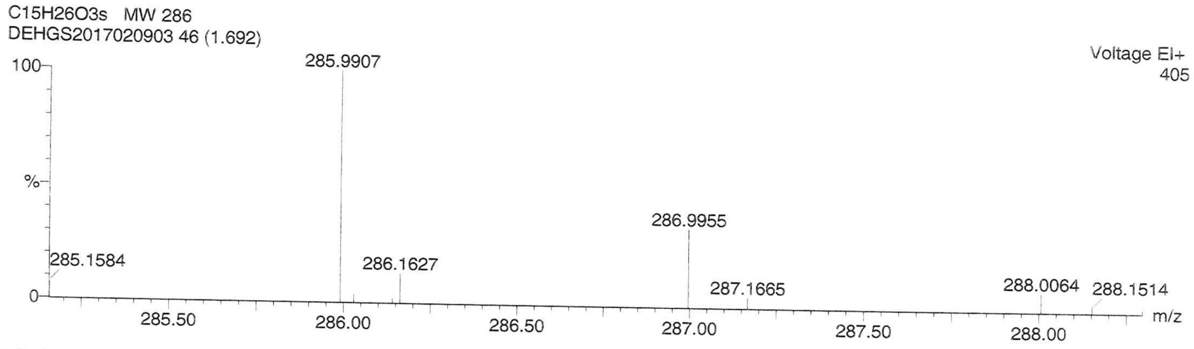

Minimum:				-1.5		
Maximum:		200.0	10.0	50.0		
Mass	Calc. Mass	mDa	PPM	DBE	Score	Formula
286.1627	286.1615	1.2	4.0	3.0	2	C15 H28 O P2
	286.1603	2.4	8.5	3.0	1	C15 H26 O3 S

Figure S-45 HRMS of compound ***pre*-13a**.

Elemental Composition Report

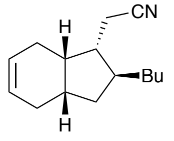

pre-**13b**

Page 1

Single Mass Analysis

Tolerance = 10.0 PPM / DBE: min = -1.5, max = 50.0
Isotope cluster parameters: Separation = 1.0 Abundance = 1.0%

Monoisotopic Mass, Odd and Even Electron Ions
25 formula(e) evaluated with 1 results within limits (up to 50 closest results for each mass)

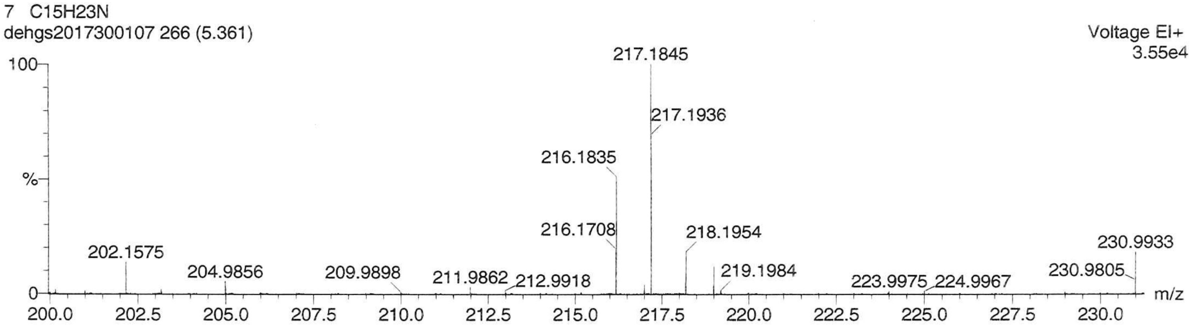

Minimum:				-1.5		
Maximum:		200.0	10.0	50.0		
Mass	Calc. Mass	mDa	PPM	DBE	Score	Formula
217.1845	217.1830	1.5	6.7	5.0	1	C15 H23 N

Figure S-46 HRMS of compound ***pre*-13b**.

Elemental Composition Report

Page 1

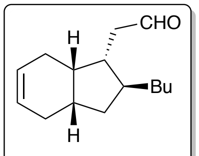

13

Single Mass Analysis

Tolerance = 10.0 PPM / DBE: min = -1.5, max = 50.0
Isotope cluster parameters: Separation = 1.0 Abundance = 1.0%

Monoisotopic Mass, Odd and Even Electron Ions
84 formula(e) evaluated with 1 results within limits (up to 50 closest results for each mass)

C15H24O MW 220
DEHGS2017020905 87 (3.204)

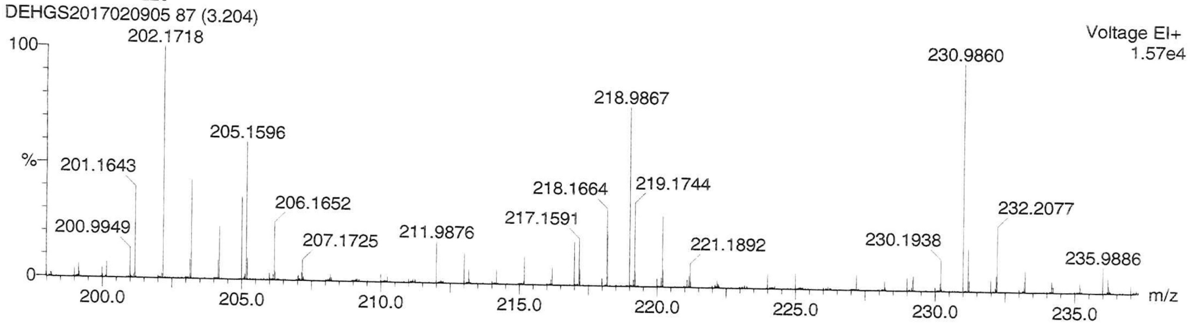

Minimum:				-1.5		
Maximum:		200.0	10.0	50.0		
Mass	Calc. Mass	mDa	PPM	DBE	Score	Formula
220.1824	220.1827	-0.3	-1.4	4.0	1	C15 H24 O

Figure S-47 HRMS of compound **13**.

Elemental Composition Report

Page 1

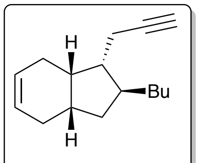

14

Single Mass Analysis

Tolerance = 10.0 PPM / DBE: min = -1.5, max = 50.0
Isotope cluster parameters: Separation = 1.0 Abundance = 1.0%

Monoisotopic Mass, Odd and Even Electron Ions
44 formula(e) evaluated with 1 results within limits (up to 50 closest results for each mass)

9 C16H24
dehgs2017300109 184 (3.708)

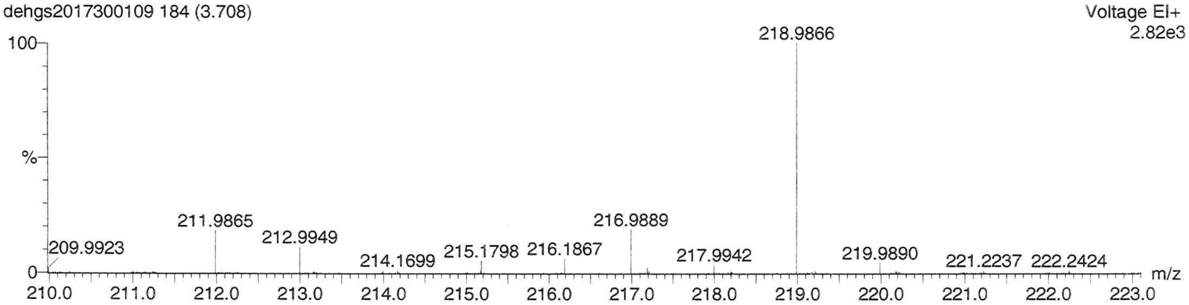

Minimum:				-1.5		
Maximum:		200.0	10.0	50.0		
Mass	Calc. Mass	mDa	PPM	DBE	Score	Formula
216.1867	216.1878	-1.1	-5.1	5.0	1	C16 H24

Figure S-48 HRMS of compound **14**.

Elemental Composition Report

Page 1

Single Mass Analysis
Tolerance = 10.0 PPM / DBE: min = -1.5, max = 50.0
Isotope cluster parameters: Separation = 1.0 Abundance = 1.0%

Monoisotopic Mass, Odd and Even Electron Ions
127 formula(e) evaluated with 1 results within limits (up to 50 closest results for each mass)

C22H36O2 MW 332
DEHGS2017020906 80 (2.942)

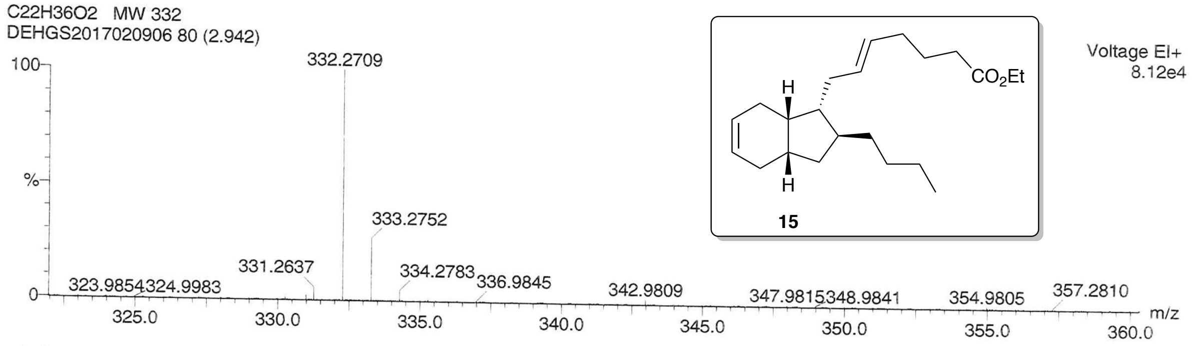

Minimum:				-1.5		
Maximum:		200.0	10.0	50.0		
Mass	Calc. Mass	mDa	PPM	DBE	Score	Formula
332.2709	332.2715	-0.6	-1.9	5.0	1	C22 H36 O2

Figure S-49 HRMS of compound **15**.

Elemental Composition Report

Page 1

Single Mass Analysis
Tolerance = 10.0 PPM / DBE: min = -1.5, max = 50.0
Isotope cluster parameters: Separation = 1.0 Abundance = 1.0%

Monoisotopic Mass, Odd and Even Electron Ions
61 formula(e) evaluated with 1 results within limits (up to 50 closest results for each mass)

C20H32O2 MW 304
DEHGS2017020908 98 (3.605)

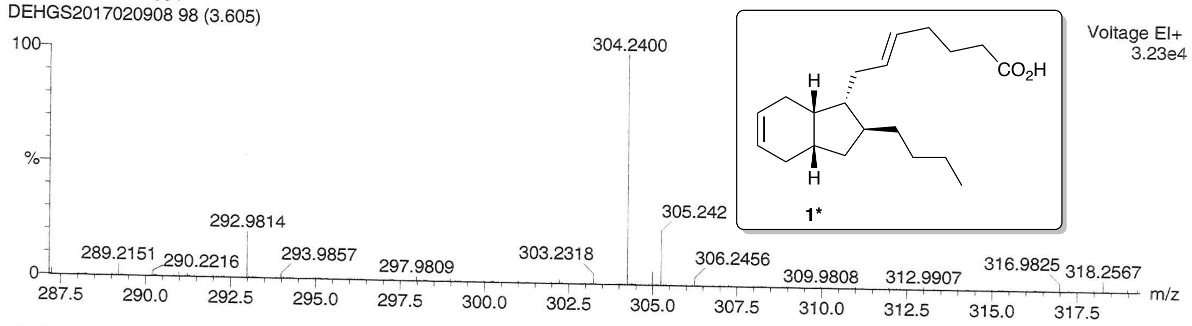

Minimum:				-1.5		
Maximum:		200.0	10.0	50.0		
Mass	Calc. Mass	mDa	PPM	DBE	Score	Formula
304.2400	304.2402	-0.2	-0.8	5.0	1	C20 H32 O2

Figure S-50 HRMS of compound **1***.

Elemental Composition Report

Page 1

Single Mass Analysis

Tolerance = 10.0 PPM / DBE: min = -1.5, max = 50.0
Isotope cluster parameters: Separation = 1.0 Abundance = 1.0%

Monoisotopic Mass, Odd and Even Electron Ions
64 formula(e) evaluated with 1 results within limits (up to 50 closest results for each mass)

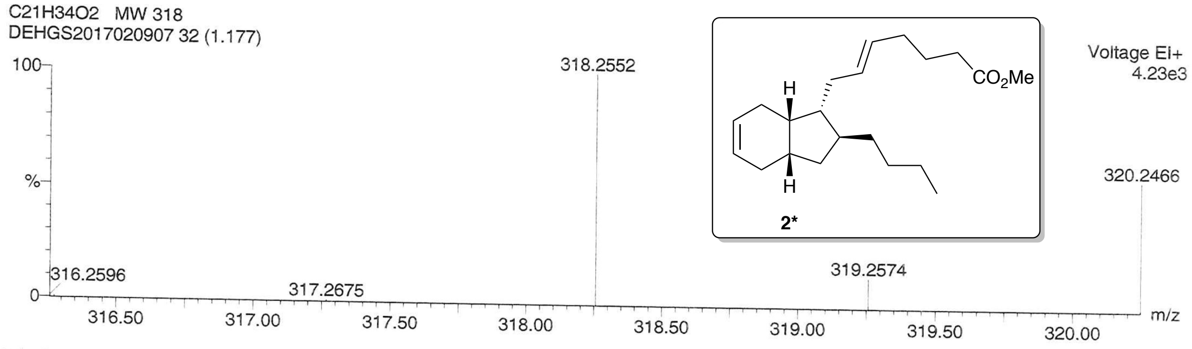

Minimum:				-1.5		
Maximum:		200.0	10.0	50.0		
Mass	Calc. Mass	mDa	PPM	DBE	Score	Formula
318.2552	318.2559	-0.7	-2.1	5.0	1	C21 H34 O2

Figure S-51 HRMS of compound **2***.

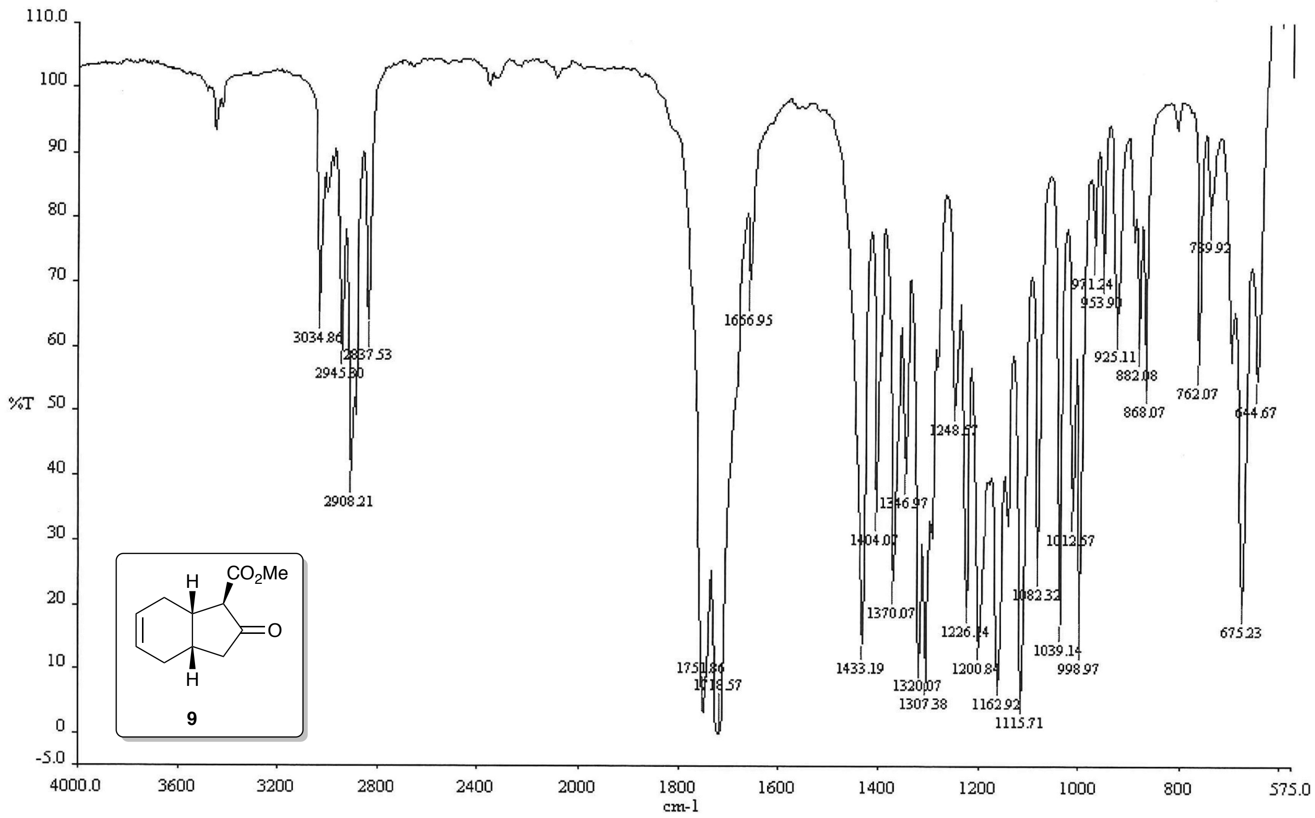

Figure S-52 IR of compound **9**.

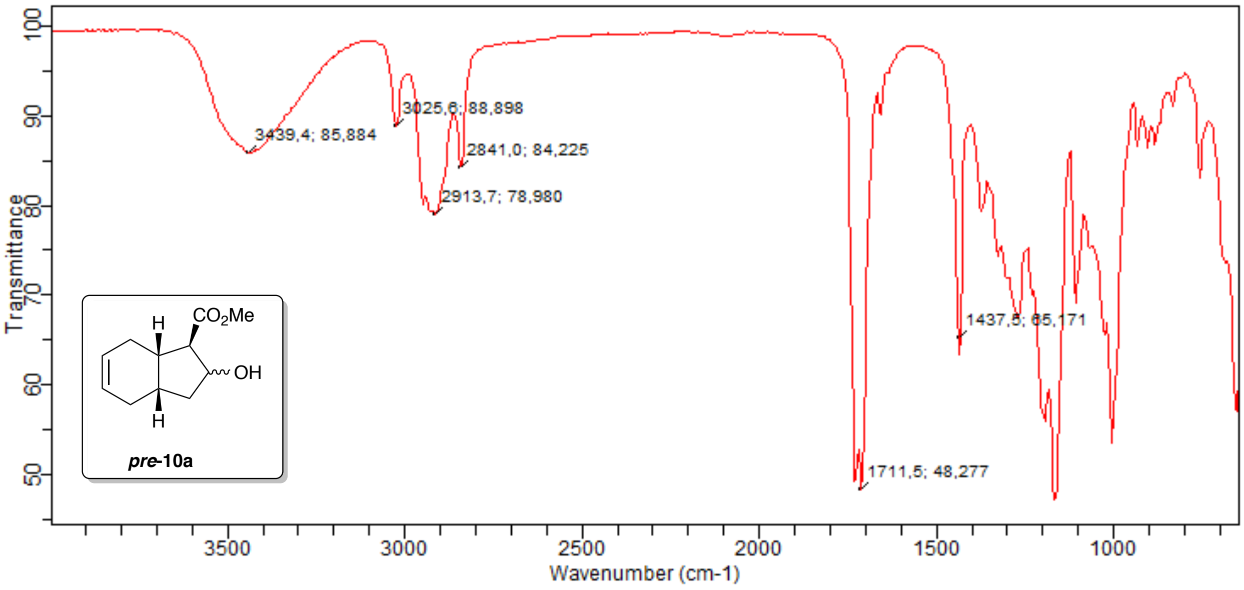

Figure S-53 IR of compound ***pre*-10a**.

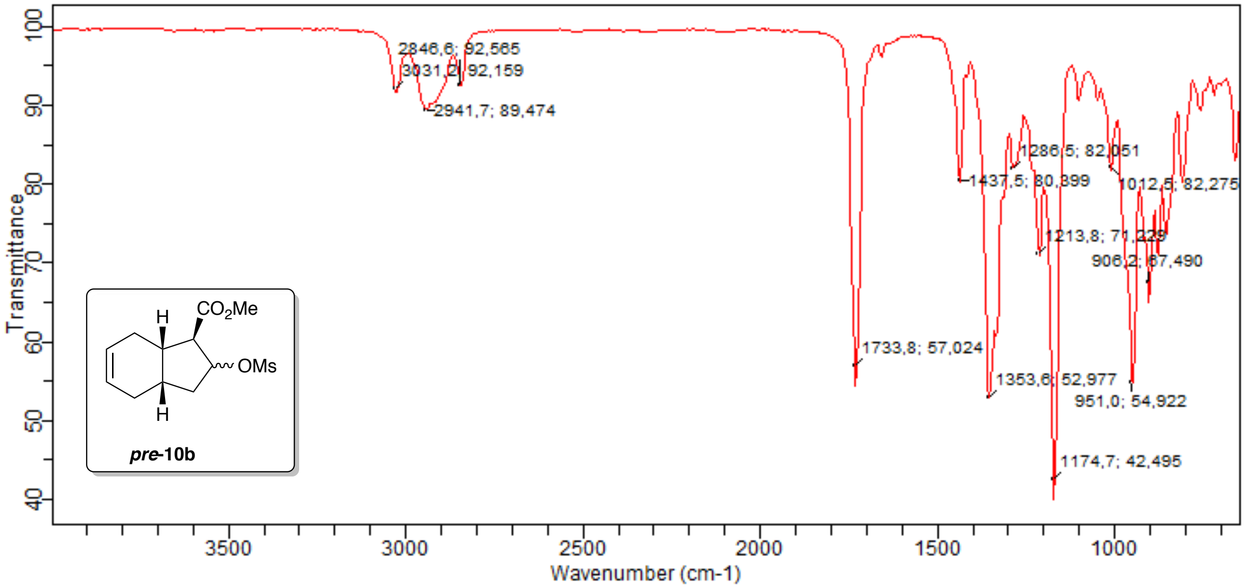

Figure S-54 IR of compound ***pre*-10b**.

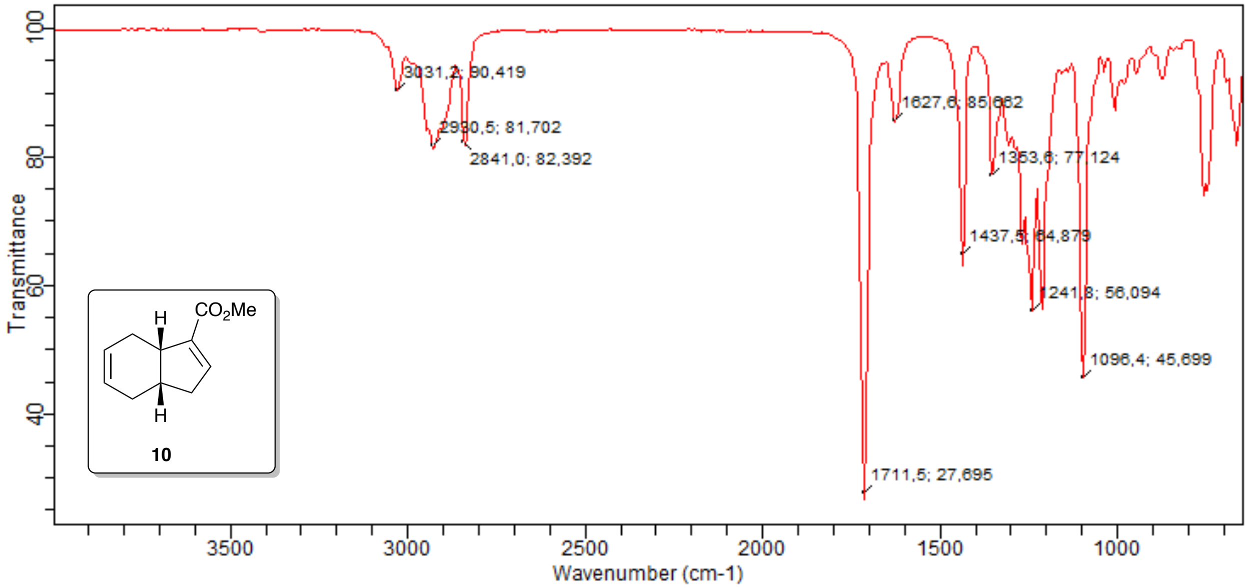

Figure S-55 IR of compound **10**.

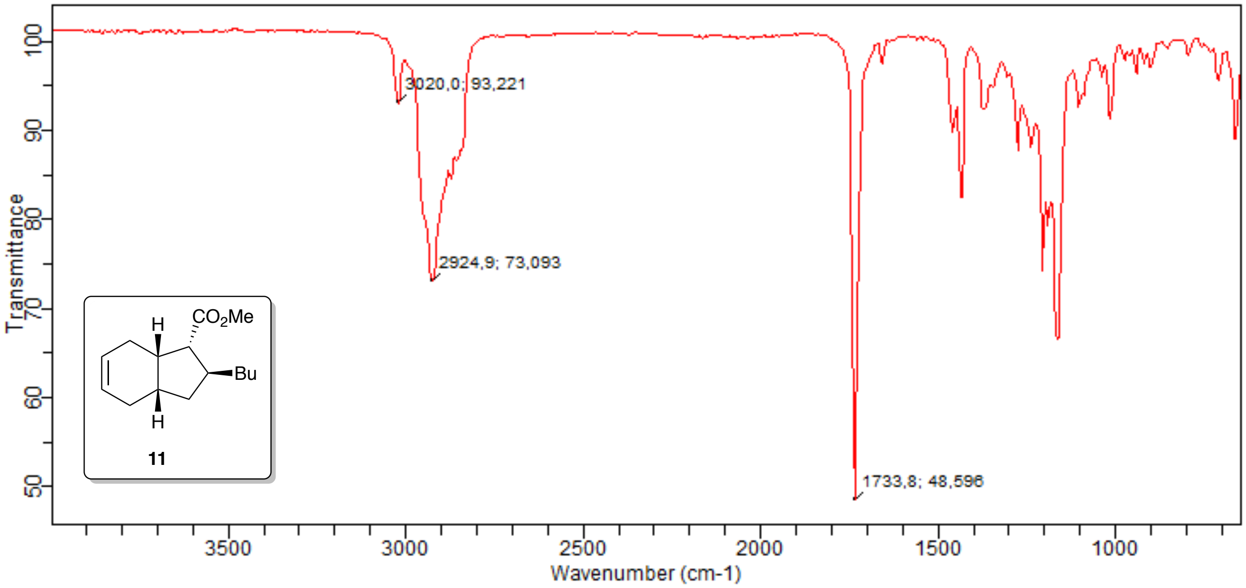

Figure S-56 IR of compound **11**.

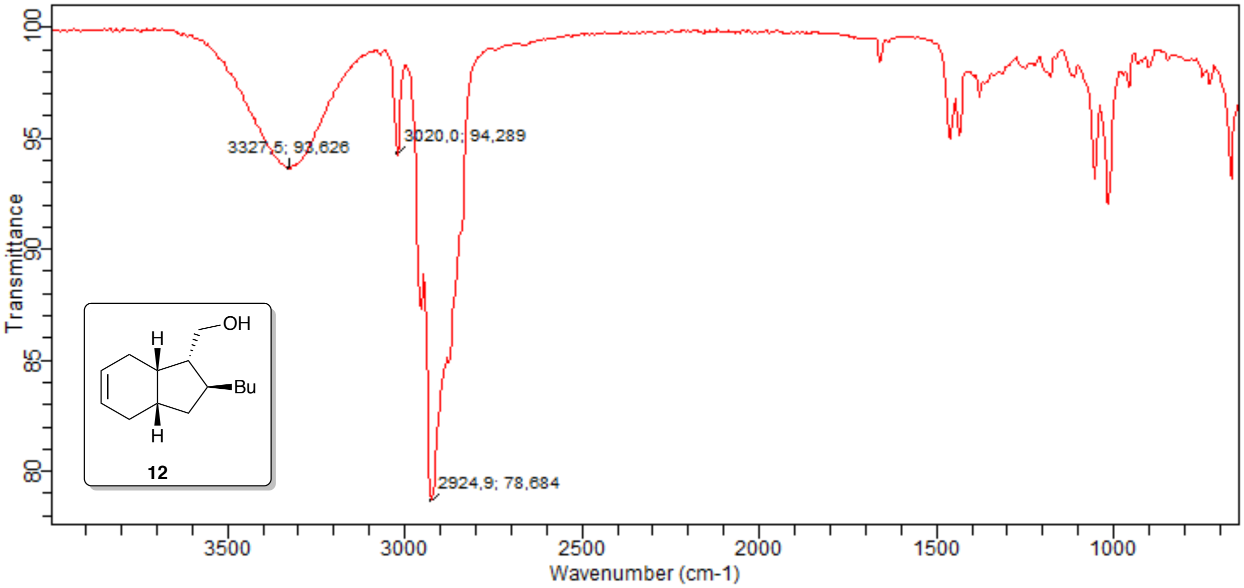

Figure S-57 IR of compound **12**.

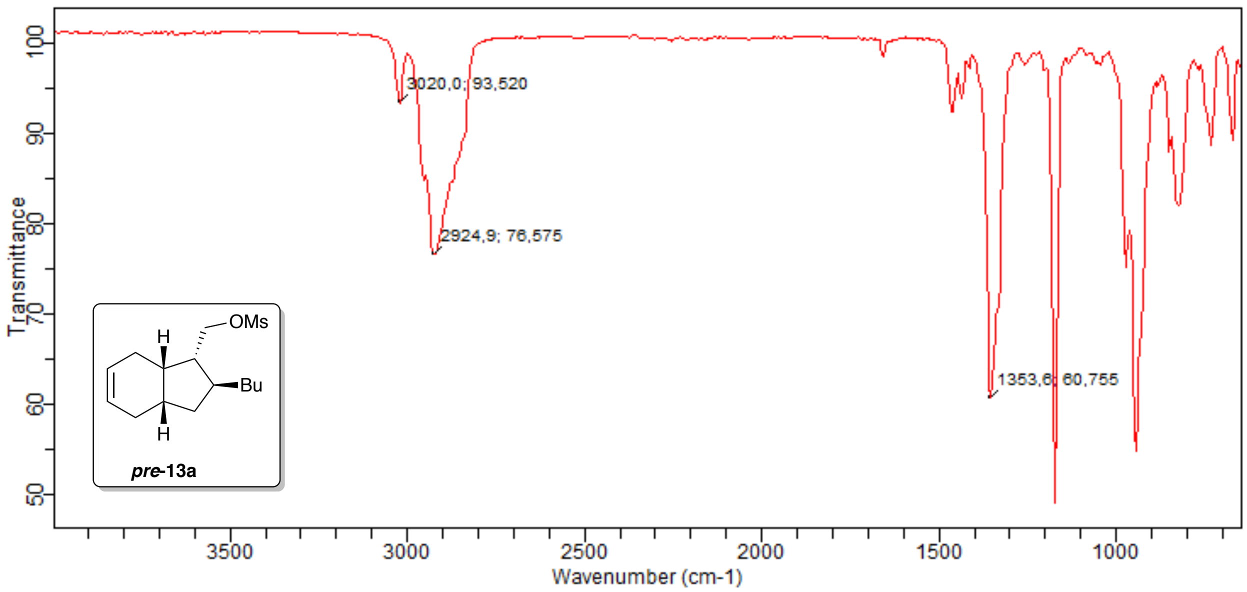

Figure S-58 IR of compound ***pre*-13a**.

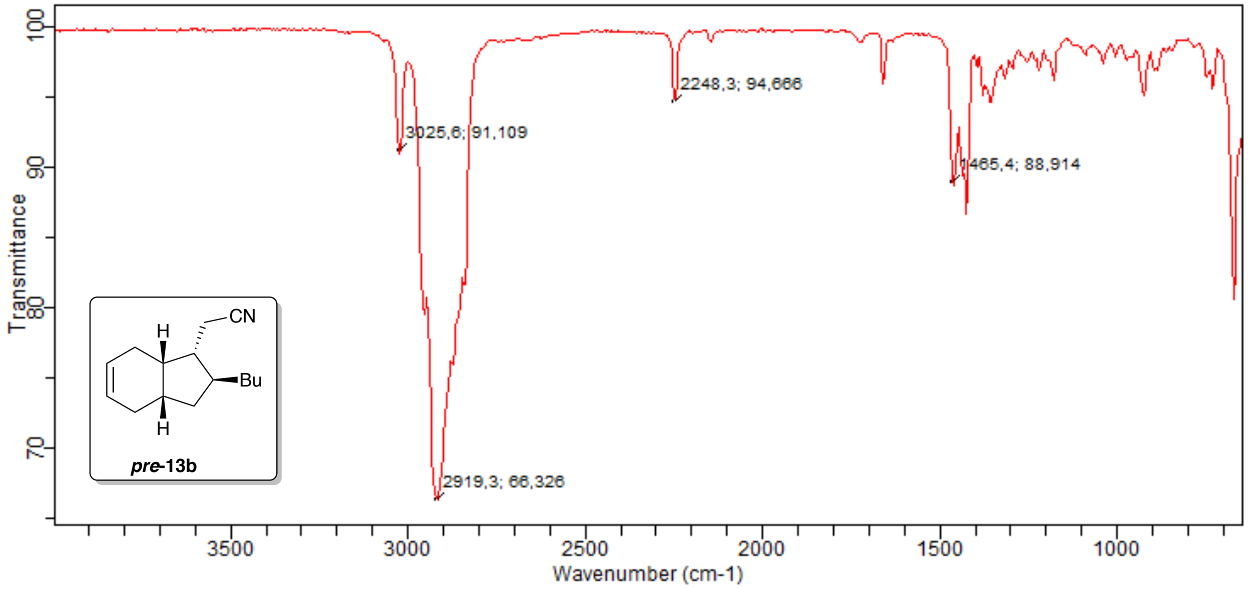

Figure S-59 IR of compound ***pre*-13b**.

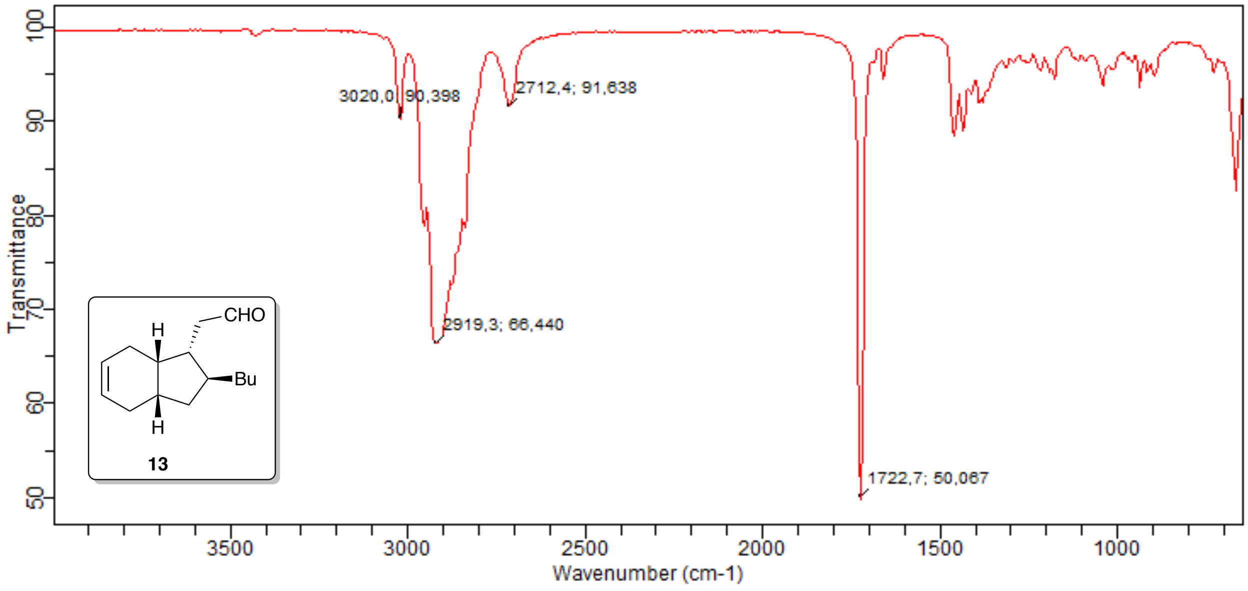

Figure S-60 IR of compound **13**.

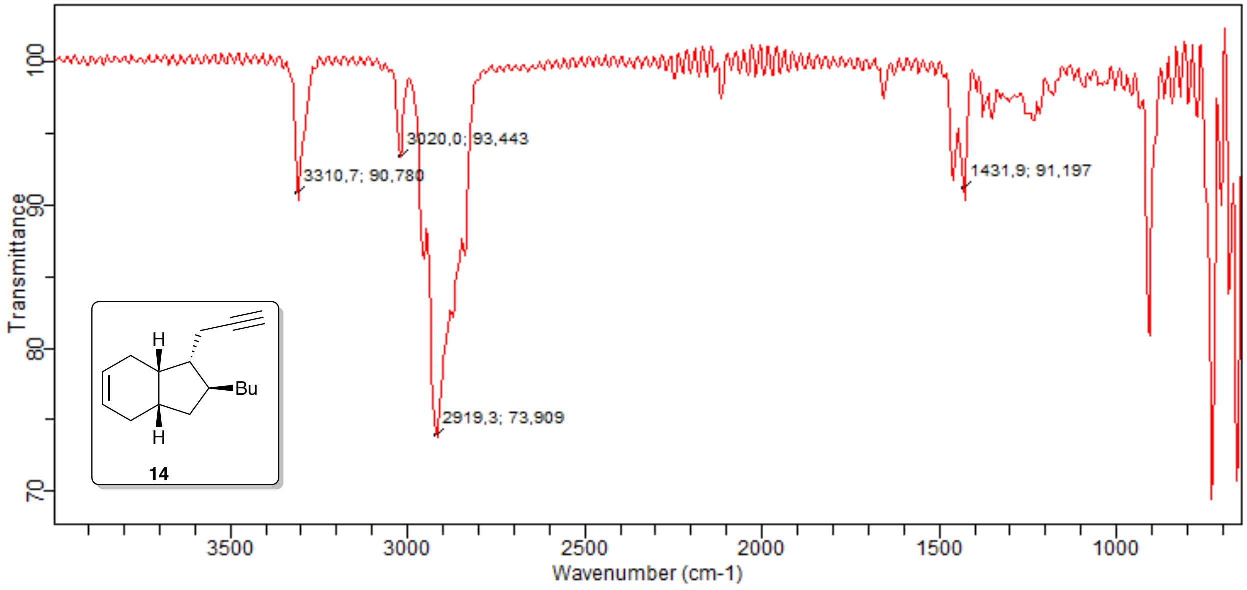

Figure S-61 IR of compound **14**.

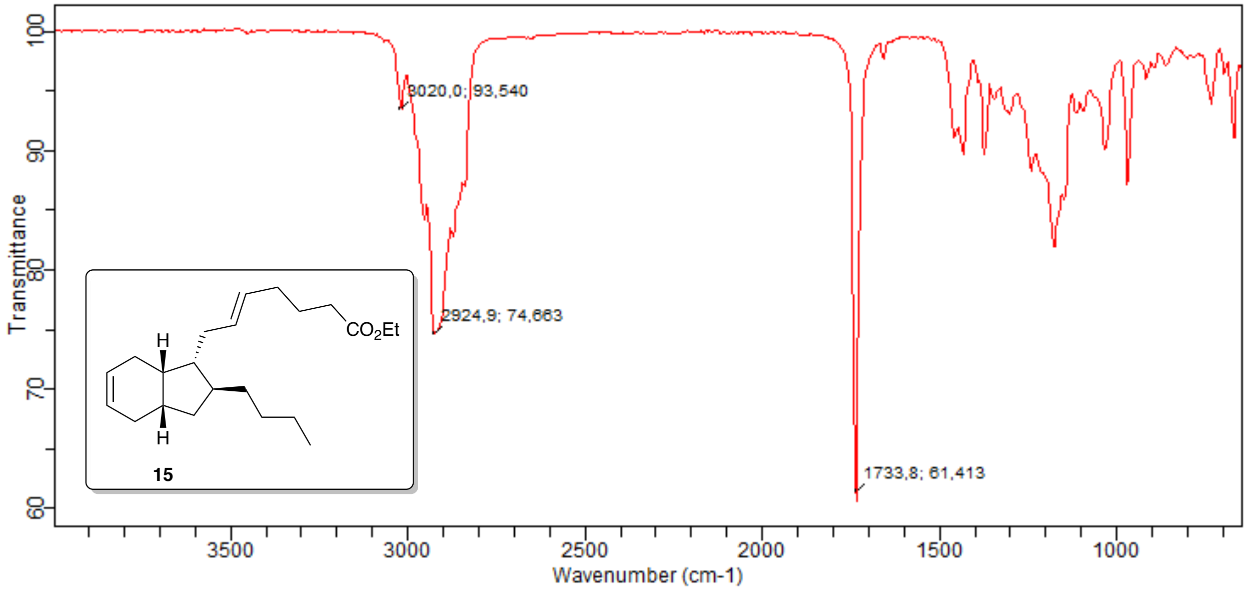

Figure S-62 IR of compound **15**.

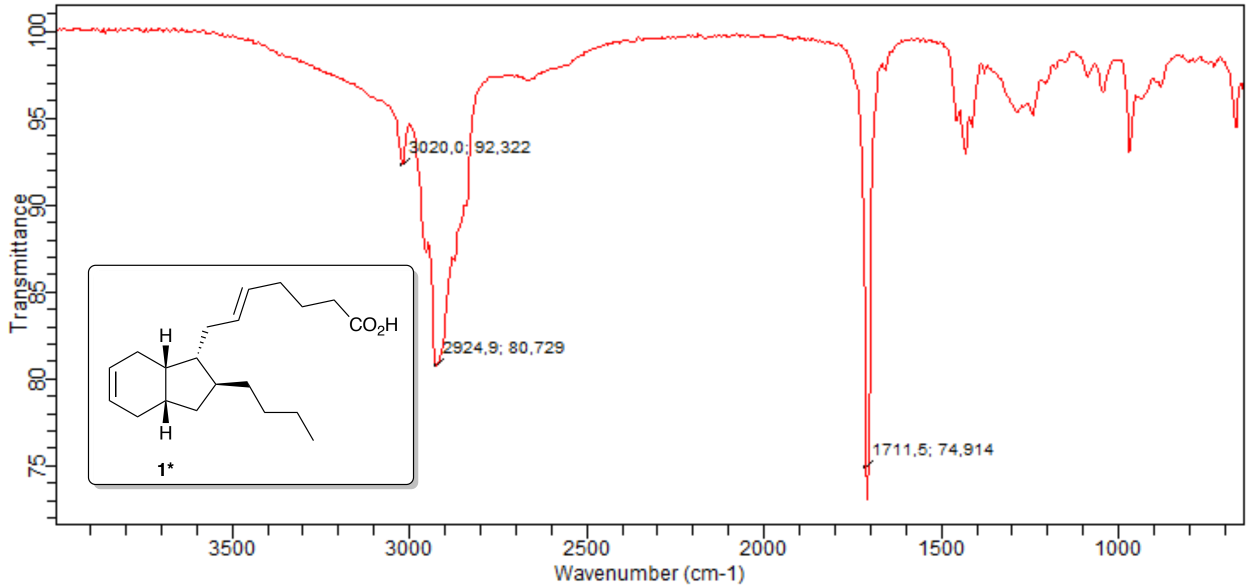

Figure S-63 IR of compound **1***.

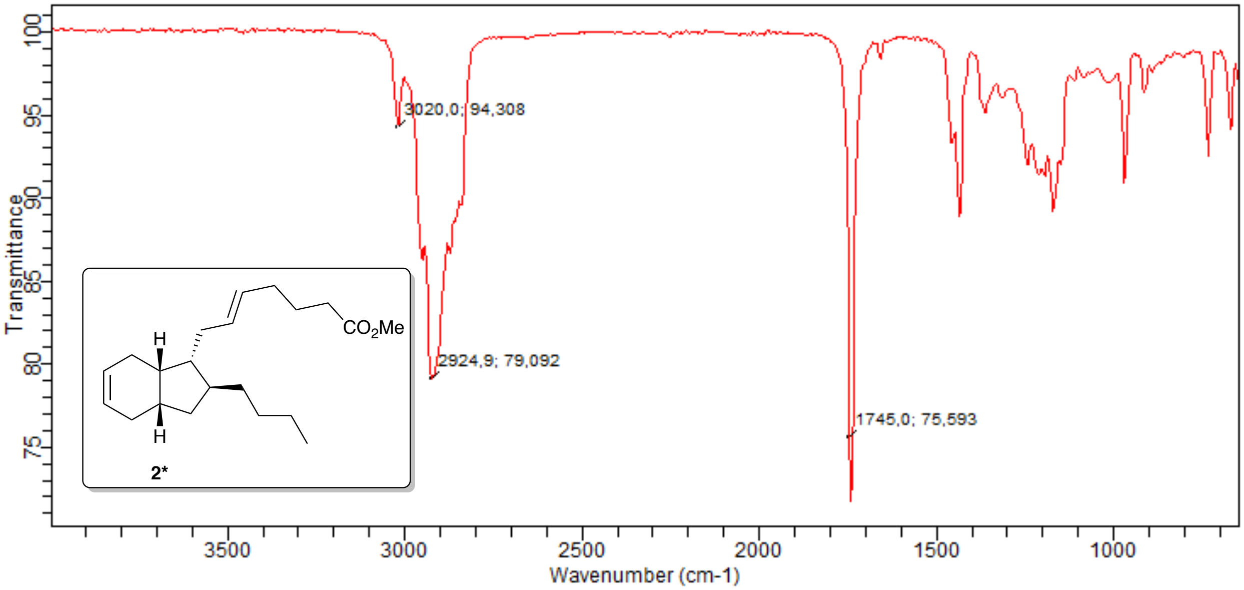

Figure S-64 IR of compound **2***.

```
Data File C:\CHEM32\1\DATA\TEST\2017-03-09H.D
Sample Name: obscuraminol diacetate LF-2015

     =====================================================================
     Acq. Operator   : SYSTEM
     Sample Operator : SYSTEM
     Acq. Instrument : 7820 GC                        Location : Vial 1
     Injection Date  : 3/9/2017 09:45:44
                                                    Inj Volume : Manually
     Method          : C:\CHEM32\1\METHODS\CP7502\CP7502.M
     Last changed    : 3/9/2017 09:39:49 by SYSTEM
     Method Info     : TVH

     Sample Info     : 80 grader 45 min, 1 grader/min til 160 grader, 5 min hold time
```

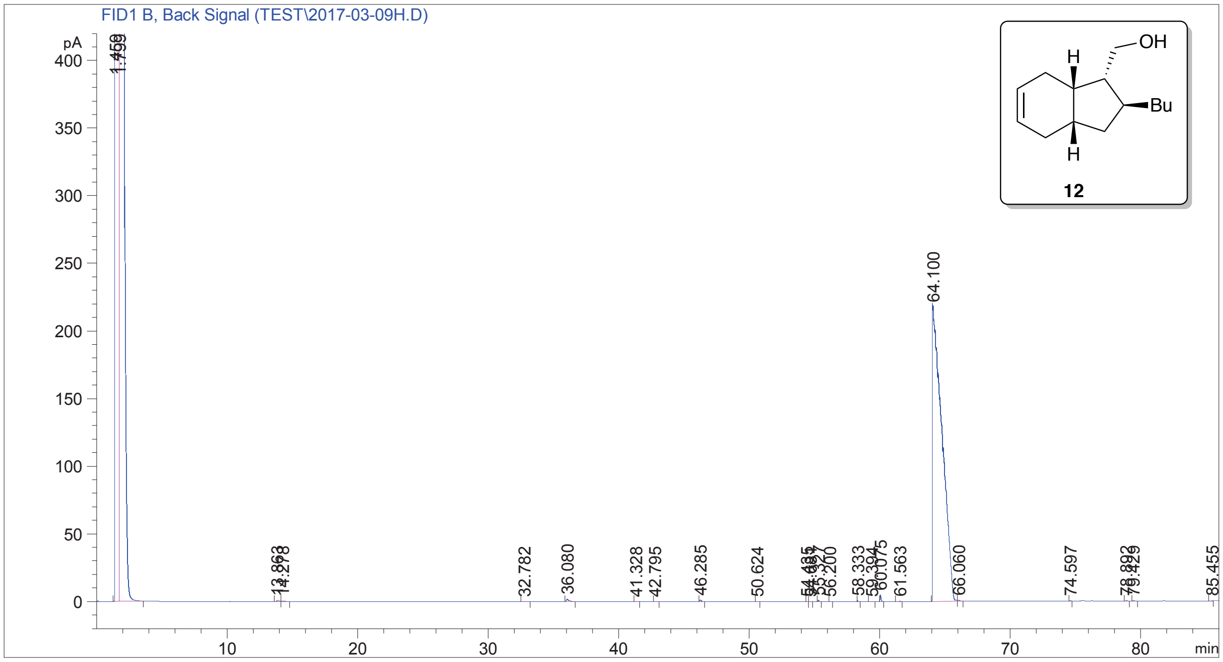


```
     =====================================================================
                           External Standard Report
     =====================================================================

     Sorted By             :      Signal
     Calib. Data Modified  :      Tuesday, August 20, 201311:04:49
     Multiplier            :      1.0000
     Dilution              :      1.0000
     Do not use Multiplier & Dilution Factor with ISTDs

     Signal 1: FID1 B, Back Signal

     RetTime  Type     Area     Amt/Area    Amount   Grp   Name
      [min]          [pA*s]               [ng/ul]
     -------|------|----------|----------|----------|--|------------------
       3.710            -          -          -         tridekan
       4.351            -          -          -         tetradekan
       4.970            -          -          -         pentadekan
       5.557            -          -          -         hexadekan

     Totals :                              0.00000
```


Figure S-65 Chiral GLC of compound **12**.

Data File C:\CHEM32\1\DATA\TEST\2017-03-09H.D
Sample Name: obscuraminol diacetate LF-2015

1 Warnings or Errors :

Warning : Calibrated compound(s) not found

===
===
Area Percent Report
===

Sorted By : Signal
Calib. Data Modified : Tuesday, August 20, 201311:04:49
Multiplier : 1.0000
Dilution : 1.0000
Do not use Multiplier & Dilution Factor with ISTDs

Signal 1: FID1 B, Back Signal

Peak #	RetTime [min]	Type	Width [min]	Area [pA*s]	Area %	Name
1	1.459	BV S	0.1133	1.90012e5	47.91768	?
2	1.799	VB S	0.1147	1.95097e5	49.20017	?
3	3.710		0.0000	0.00000	0.00000	tridekan
4	4.351		0.0000	0.00000	0.00000	tetradekan
5	4.970		0.0000	0.00000	0.00000	pentadekan
6	5.557		0.0000	0.00000	0.00000	hexadekan
7	13.863	BV	0.2370	12.08627	0.00305	?
8	14.278	VB	0.2547	7.78858	0.00196	?
9	32.782	BB	0.2431	3.14527	0.00079	?
10	36.080	BB	0.2167	29.80929	0.00752	?
11	41.328	BB	0.1531	1.40514	0.00035	?
12	42.795	BB	0.1564	1.44569	0.00036	?
13	46.285	VB	0.1344	11.81501	0.00298	?
14	50.624	BB	0.1101	1.27909	0.00032	?
15	54.435	BV	0.0847	1.04445	0.00026	?
16	54.681	VB	0.0823	1.10538	0.00028	?
17	55.327	BB	0.0923	6.13038	0.00155	?
18	56.200	VB	0.0876	1.11939	0.00028	?
19	58.333	BB	0.0702	2.48628	0.00063	?
20	59.394	BB	0.1260	1.48444	0.00037	?
21	60.075	BB	0.0828	26.48581	0.00668	?
22	61.563	VB	0.0945	2.07171	0.00052	?
23	64.100	BV	0.6207	1.13029e4	2.85040	?
24	66.060	VB	0.1664	9.06356	0.00229	?
25	74.597	BB	0.0844	1.00437	0.00025	?
26	78.892	BB	0.1327	1.10520	0.00028	?
27	79.429	BB	0.1510	1.31295	0.00033	?
28	85.455	BV	0.1721	2.73978	0.00069	?

Totals : 3.96538e5

1 Warnings or Errors :

Warning : Calibrated compound(s) not found

Figure S-66 Report from chiral GLC of compound **12**.

Data File C:\CHEM32\1\DATA\TEST\2017-03-08H.D
Sample Name: obscuraminol diacetate LF-2015

===

Acq. Operator : SYSTEM
Sample Operator : SYSTEM
Acq. Instrument : 7820 GC   Location : Vial 1
Injection Date : 3/8/2017 12:46:11
Inj Volume : Manually
Acq. Method : C:\CHEM32\1\METHODS\CP7502\CP7502.M
Last changed : 3/8/2017 10:20:12 by SYSTEM
Analysis Method : C:\CHEM32\1\METHODS\CP7502\CP7502.M
Last changed : 3/9/2017 09:39:49 by SYSTEM
Method Info : TVH

Sample Info : 80 grader 45 min, 1 grader/min til 160 grader, 5 min hold time

Additional Info : Peak(s) manually integrated

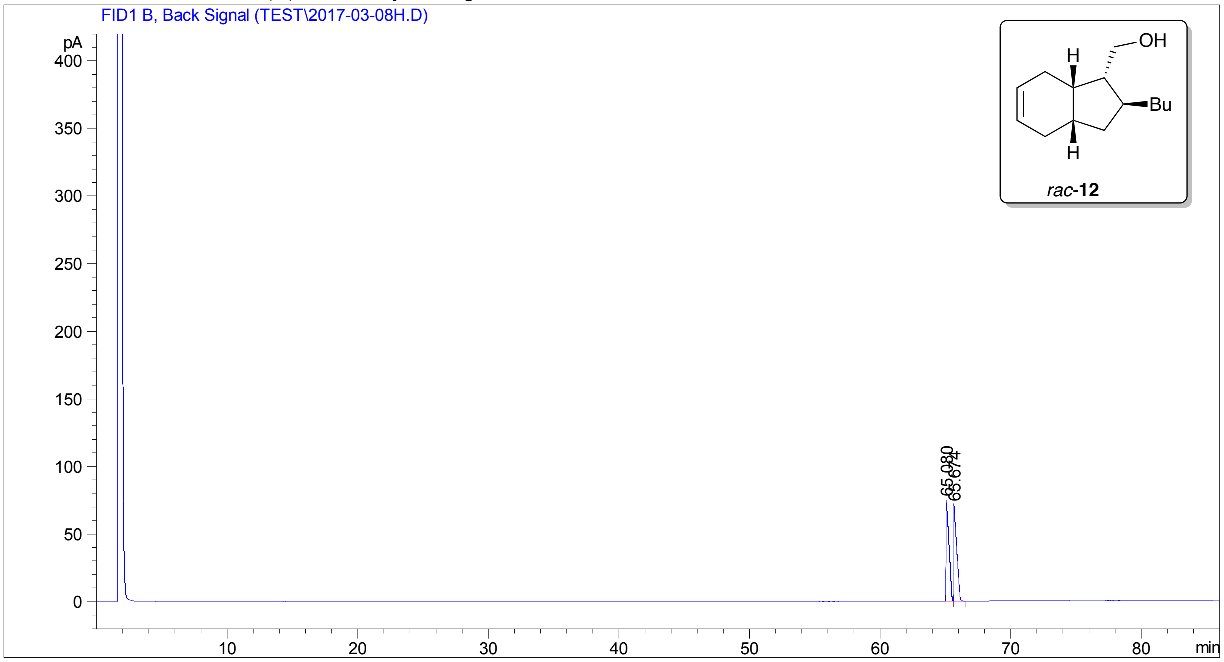

===
External Standard Report
===

Sorted By : Signal
Calib. Data Modified : Tuesday, August 20, 201311:04:49
Multiplier : 1.0000
Dilution : 1.0000
Do not use Multiplier & Dilution Factor with ISTDs

Signal 1: FID1 B, Back Signal

RetTime [min]	Type	Area [pA*s]	Amt/Area	Amount [ng/ul]	Grp	Name
3.710		-	-	-		tridekan
4.351		-	-	-		tetradekan
4.970		-	-	-		pentadekan

Figure S-67 Chiral GLC of compound *rac*-**12**.

Data File C:\CHEM32\1\DATA\TEST\2017-03-08H.D
Sample Name: obscuraminol diacetate LF-2015

RetTime [min]	Type	Area [pA*s]	Amt/Area	Amount [ng/ul]	Grp	Name
5.557		-	-	-		hexadekan

Totals : 0.00000

1 Warnings or Errors :

Warning : Calibrated compound(s) not found

Area Percent Report

Sorted By : Signal
Calib. Data Modified : Tuesday, August 20, 201311:04:49
Multiplier : 1.0000
Dilution : 1.0000
Do not use Multiplier & Dilution Factor with ISTDs

Signal 1: FID1 B, Back Signal

Peak #	RetTime [min]	Type	Width [min]	Area [pA*s]	Area %	Name
1	3.710		0.0000	0.00000	0.00000	tridekan
2	4.351		0.0000	0.00000	0.00000	tetradekan
3	4.970		0.0000	0.00000	0.00000	pentadekan
4	5.557		0.0000	0.00000	0.00000	hexadekan
5	65.080	BV	0.2045	1158.40588	50.35220	?
6	65.674	VB	0.2139	1142.20020	49.64780	?

Totals : 2300.60608

1 Warnings or Errors :

Warning : Calibrated compound(s) not found

*** End of Report ***

7820 GC 3/9/2017 09:42:21 SYSTEM

Figure S-68 Report from chiral GLC of compound *rac*-**12**.

Preparation 3,5-dinitrobenzoate derivative of (12).

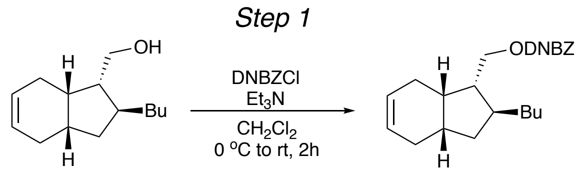

Scheme S-3 Derivatization of advanced intermediate **12** to 3,5-dinitrobenzoate **12-DNB**.

(1*S*,6*S*,7*R*,8*S*)-8-Butyl-7-((3,5-dinitrobenzoyl)oxymethyl)bicyclo[4.3.0]non-3-ene (12-DNB).

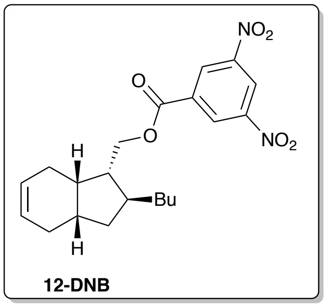

A stirring solution of (1*S*,6*S*,7*S*,8*R*)-8-butyl-7-(hydroxymethyl)bicyclo[4.3.0]non-3-ene **12** (0.129 g, 0.546 mmol, 1.0 equiv.) in dry DCM (20 mL) was added Et_3N (0.23 mL, 1.64 mmol, 3.0 equiv.) dropwise. The solution was then cooled to 0 °C and 3,5-dinitrobenzoyl chloride (0.215 g, 0.933 mmol, 1.7 equiv.) was added in one portion. The reaction was slowly warmed to room temperature and monitored by TLC until completion. After 2h, the reaction mixture was poured over H_2O (10 mL) and the organic layer separated. The aqueous layer was then extracted with DCM (2 x 10 mL) and the organic layers combined. The organic layers were then washed with H_2O (1 x 30 mL), brine (1 x 30 mL), dried with $MgSO_4$, filtered and concentrated *in vacuo* to form a crude orange oil. This was purified by column chromatography on silica (hexane/EtOAc, 95:5) to afford the title compound as a white powder. Yield: 0.193 g (88%), $[\alpha]_D^{26} + 42$ ($c\ =\ 0.8$, $CHCl_3$); ^{1}H NMR (400 MHz, $CDCl_3$) δ 9.23 (t, $J =$ 2.2 Hz, 1H), 9.14 (d, $J = 2.2$ Hz, 2H), 5.70-5.61 (m, 2H), 4.49 (s, 1H), 4.47 (d, $J = 1.9$ Hz, 1H), 2.37-2.10 (m, 4H), 2.02-1.88 (m, 2H), 1.88-1.68 (m, 3H), 1.58-1.43 (m, 2H), 1.40-1.23 (m, 5H), 0.89 (t, $J = 6.7$ Hz, 3H); ^{13}C NMR (100 MHz, $CDCl_3$) δ 162.5, 148.7, 134.1, 129.3, 125.4, 124.3, 122.3, 67.7, 49.5, 38.6, 36.9, 36.7, 35.5, 35.4, 30.8, 26.5, 22.9, 21.8, 14.1; IR (neat, cm^{-1}) 3098 (w), 3020 (w), 2931 (m), 1723 (s), 1538 (s); HRMS (EI+): Exact mass calculated for $C_{21}H_{26}N_2O_6$ $[M]^+$: 402.1791, found 402.1797; m.p.: 117 °C; TLC (hexane/EtOAc 4:1, $KMnO_4$ stain): R_f =0.75.

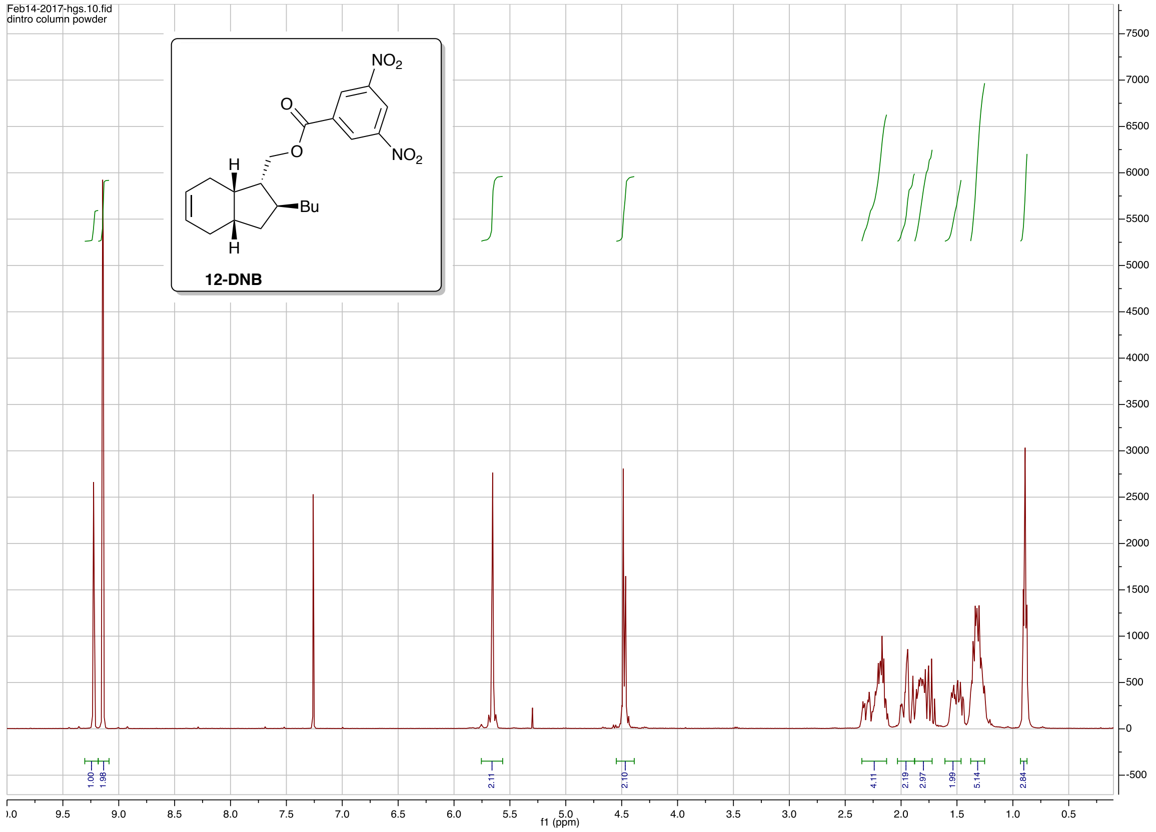

Figure S-69 ^{1}H-NMR spectrum of compound **12-DNB**.

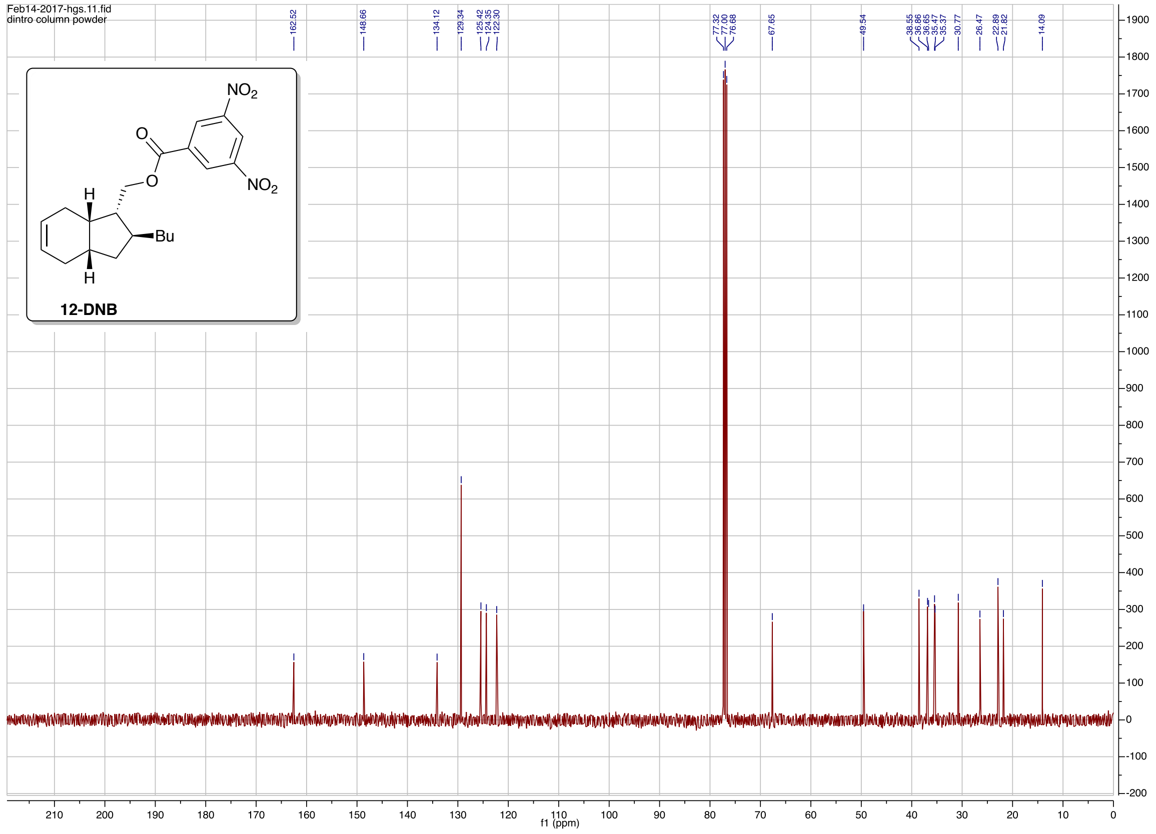

Figure S-70 ^{13}C-NMR spectrum of compound **12-DNB**.

Elemental Composition Report

Single Mass Analysis

Tolerance = 10.0 PPM / DBE: min = -1.5, max = 50.0
Isotope cluster parameters: Separation = 1.0 Abundance = 1.0%

Monoisotopic Mass, Odd and Even Electron Ions
107 formula(e) evaluated with 2 results within limits (up to 50 closest results for each mass)

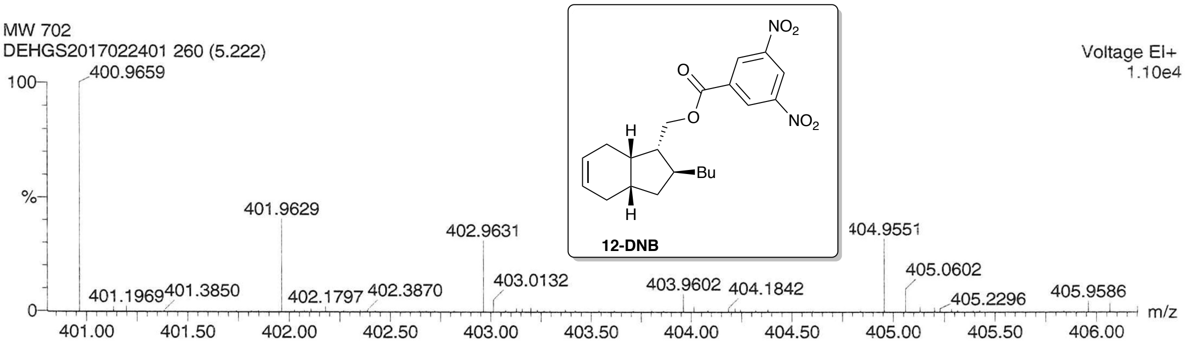

Minimum:				-1.5		
Maximum:		200.0	10.0	50.0		
Mass	Calc. Mass	mDa	PPM	DBE	Score	Formula
402.1797	402.1791	0.6	1.5	10.0	2	C21 H26 N2 O6
	402.1831	-3.4	-8.5	14.0	1	C26 H26 O4

Figure S-71 HRMS spectrum of compound **12-DNB**.

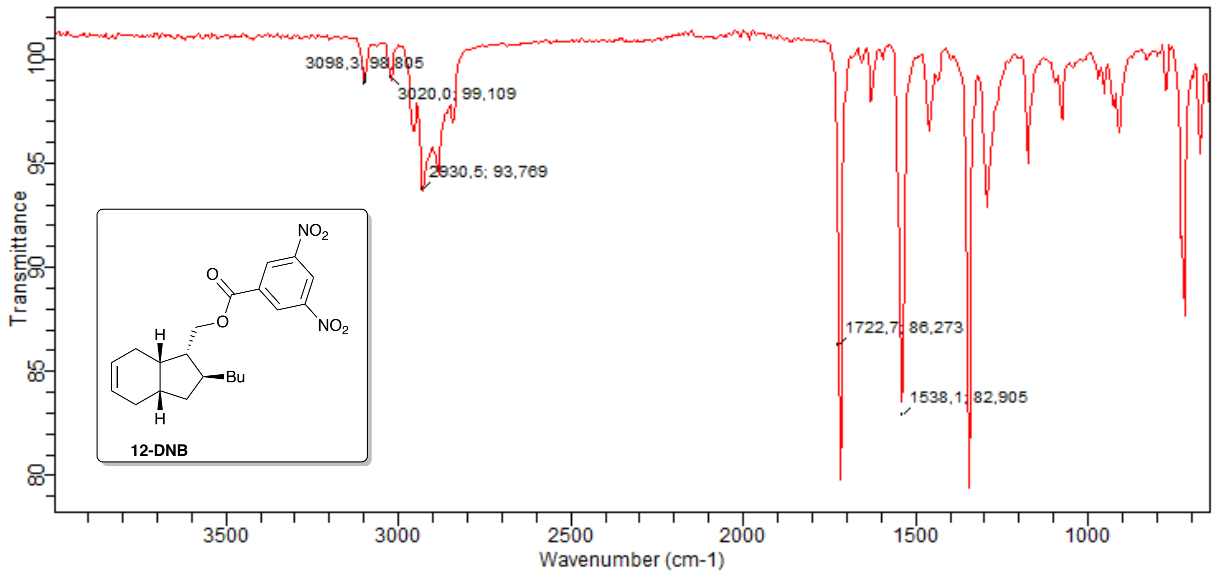

Figure S-72 IR spectrum of compound **12-DNB**.

X-ray crystallography on compound (12-DNB):

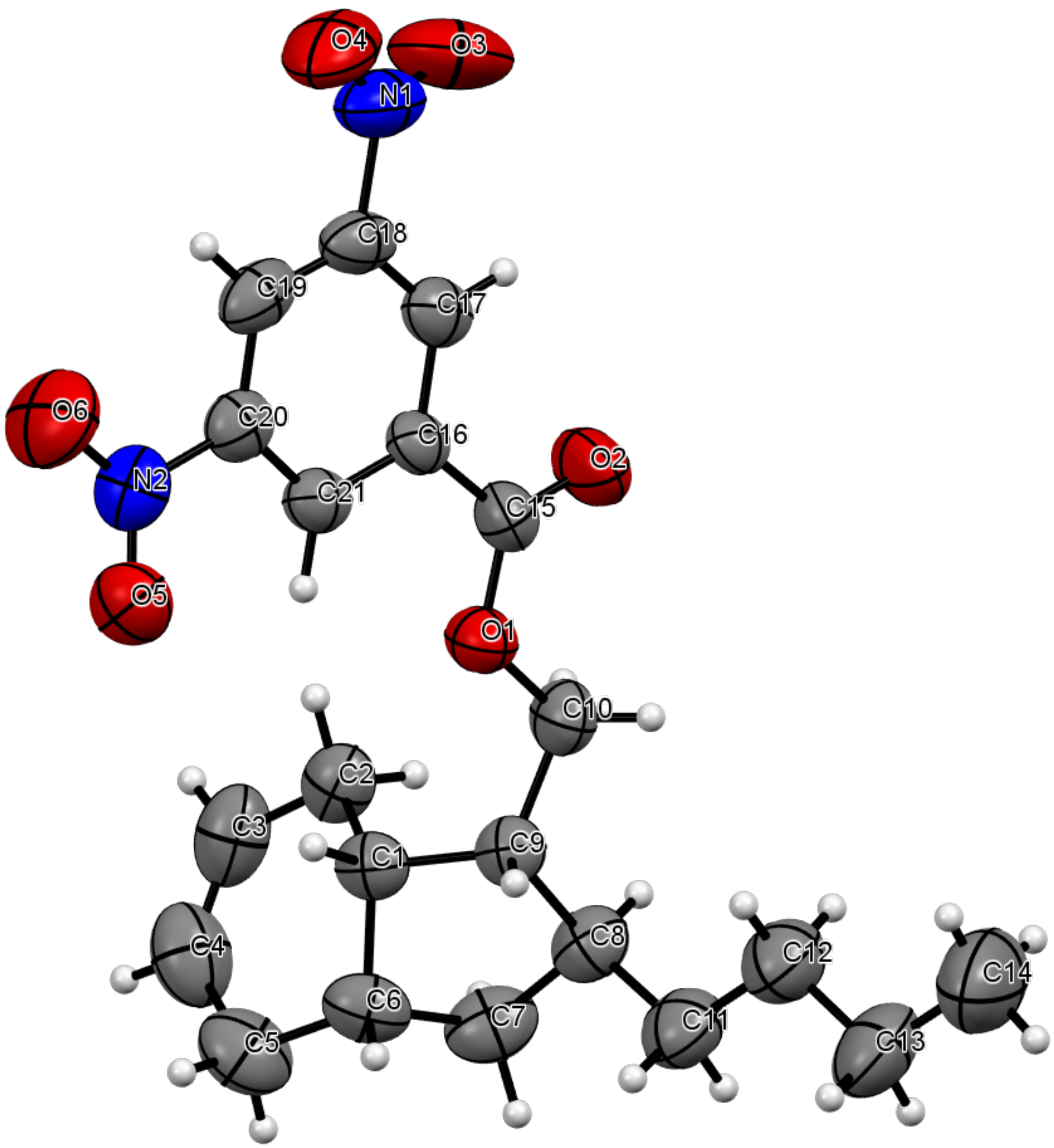

Figure S-73 ORTEP plot of compound **12-DNB** (top perspective).

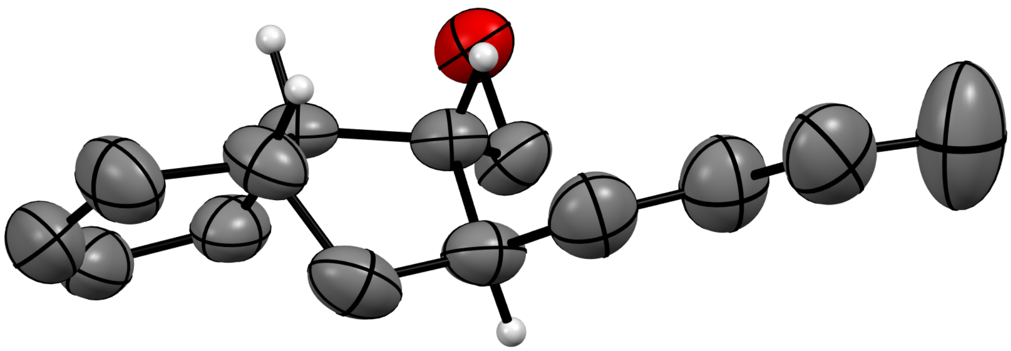

Figure S-74 ORTEP plot of compound **12-DNB** (side perspective: non-stereogenic protons and DNB group omitted).

supporting information

Pursuing the true structure of marine natural product mucosin. Part 2.

Computing details

Data collection: *APEX3* (Bruker, 2016); cell refinement: *APEX3* (Bruker, 2016); data reduction: *APEX3* (Bruker, 2016); program(s) used to solve structure: *APEX3* (Bruker, 2016); program(s) used to refine structure: *SHELXT* (Sheldrick, 2015a); molecular graphics: *Mercury* (Macrae *et al.*, 2008); software used to prepare material for publication: *SHELXL2014* (Sheldrick, 2015b).

(1*S*,6S,7*R*,8S)-8-Butyl-7-((3,5- dinitrobenzoyl)oxymethyl)bicyclo[4.3.0]non-3-ene

Crystal data

$C_{21}H_{26}N_2O_6$
M_r = 402.44
Orthorhombic, $P2_12_12_1$
a = 5.0886 (5) Å
b = 11.5288 (11) Å
c = 35.602 (3) Å
V = 2088.6 (3) Å^3
Z = 4
F(000) = 856
D_x = 1.280 Mg m^{-3}
Mo $K\alpha$ radiation, λ = 0.71073 Å
Cell parameters from 3545 reflections
θ = 2.5–23.1°
μ = 0.09 mm^{-1}
T = 297 K
Flat needle, colorless
0.81 × 0.23 × 0.03 mm

Data collection

Bruker D8 Venture with a Photon 100 CMOS detector diffractometer
Radiation source: fine-focus sealed tube
Graphite monochromator
Detector resolution: 8.3 pixels mm^{-1}
Sets of exposures each taken over 0.5° ω rotation scans
Absorption correction: multi-scan *SADABS* (Bruker, 2016)
T_{min} = 0.906, T_{max} = 1.000
9297 measured reflections
3950 independent reflections
2611 reflections with $I > 2\sigma(I)$
R_{int} = 0.030
θ_{max} = 25.7°, θ_{min} = 2.5°
h = −4→6
k = −14→14
l = −43→33

Refinement

Refinement on F^2
Least-squares matrix: full
$R[F^2 > 2\sigma(F^2)]$ = 0.053
$wR(F^2)$ = 0.124
S = 1.05
3950 reflections
263 parameters
0 restraints
Hydrogen site location: inferred from neighbouring sites
H-atom parameters constrained
$w = 1/[\sigma^2(F_o^2) + (0.0508P)^2 + 0.326P]$ where $P = (F_o^2 + 2F_c^2)/3$
$(\Delta/\sigma)_{max}$ < 0.001
$\Delta\rho_{max}$ = 0.14 e Å^{-3}
$\Delta\rho_{min}$ = −0.16 e Å^{-3}
Absolute structure: Flack x determined using 803 quotients [(I+)-(I-)]/[(I+)+(I-)] (Parsons, Flack and Wagner, Acta Cryst. B69 (2013) 249-259).
Absolute structure parameter: −0.9 (6)

Special details

Geometry. All e.s.d.'s (except the e.s.d. in the dihedral angle between two l.s. planes) are estimated using the full covariance matrix. The cell e.s.d.'s are taken into account individually in the estimation of e.s.d.'s in distances, angles and torsion angles; correlations between e.s.d.'s in cell parameters are only used when they are defined by crystal symmetry. An approximate (isotropic) treatment of cell e.s.d.'s is used for estimating e.s.d.'s involving l.s. planes.

Fractional atomic coordinates and isotropic or equivalent isotropic displacement parameters (Å²)

	x	*y*	*z*	U_{iso}*/U_{eq}
C1	0.1952 (8)	0.3211 (3)	0.32542 (10)	0.0657 (10)
H1	0.3773	0.3354	0.3177	0.079*
C2	0.1407 (10)	0.1930 (4)	0.32050 (13)	0.0889 (14)
H21	0.0939	0.1785	0.2945	0.107*
H22	0.2999	0.1499	0.3259	0.107*
C3	−0.0722 (10)	0.1499 (4)	0.34495 (15)	0.0912 (14)
H3	−0.1344	0.0751	0.3409	0.109*
C4	−0.1797 (9)	0.2110 (4)	0.37227 (14)	0.0814 (12)
H4	−0.3115	0.1762	0.3864	0.098*
C5	−0.1037 (7)	0.3314 (3)	0.38181 (10)	0.0645 (10)
H51	−0.0969	0.3392	0.4089	0.077*
H52	−0.2379	0.3839	0.3726	0.077*
C6	0.1622 (6)	0.3673 (3)	0.36535 (9)	0.0520 (9)
H6	0.3051	0.3388	0.3813	0.062*
C9	0.0196 (8)	0.4038 (4)	0.30336 (10)	0.0751 (12)
H91	0.0678	0.4039	0.2770	0.090*
H92	−0.1632	0.3810	0.3056	0.090*
C7	0.1905 (7)	0.4977 (3)	0.35926 (8)	0.0533 (9)
H7	0.3786	0.5146	0.3570	0.064*
C8	0.0627 (8)	0.5224 (3)	0.32047 (9)	0.0625 (10)
H8	−0.1077	0.5602	0.3243	0.075*
C10	0.0829 (8)	0.5738 (3)	0.38977 (9)	0.0633 (10)
H101	0.1131	0.6548	0.3838	0.076*
H102	−0.1048	0.5616	0.3924	0.076*
C11	0.2342 (10)	0.5985 (4)	0.29485 (10)	0.0817 (13)
H111	0.3996	0.5586	0.2907	0.098*
H112	0.1473	0.6051	0.2707	0.098*
C12	0.2924 (12)	0.7161 (4)	0.30846 (12)	0.0915 (14)
H121	0.3886	0.7103	0.3319	0.110*
H122	0.1280	0.7556	0.3136	0.110*
C13	0.4519 (12)	0.7890 (4)	0.28093 (12)	0.1029 (16)
H131	0.6242	0.7543	0.2778	0.124*
H132	0.3652	0.7887	0.2567	0.124*
C14	0.4825 (19)	0.9093 (5)	0.29375 (17)	0.153 (3)
H141	0.5852	0.9518	0.2758	0.229*
H142	0.5699	0.9101	0.3177	0.229*
H143	0.3126	0.9447	0.2962	0.229*
O1′	0.2180 (5)	0.54315 (19)	0.42458 (6)	0.0607 (7)
O2′	−0.0468 (7)	0.6630 (3)	0.45574 (8)	0.0954 (10)
O3′	0.0468 (10)	0.6832 (4)	0.59179 (10)	0.1279 (16)
O4′	0.4052 (10)	0.6242 (3)	0.61725 (9)	0.1141 (14)
O5′	0.8863 (5)	0.3287 (2)	0.49051 (9)	0.0784 (8)
O6′	0.8736 (7)	0.3167 (3)	0.55058 (9)	0.1096 (12)
N1′	0.2532 (12)	0.6308 (3)	0.59142 (11)	0.0878 (13)
N2′	0.8003 (7)	0.3561 (3)	0.52080 (11)	0.0683 (9)
C1′	0.2723 (7)	0.5574 (3)	0.48966 (9)	0.0509 (8)
C2′	0.2014 (8)	0.6073 (3)	0.52338 (10)	0.0599 (9)
H2′	0.0676	0.6622	0.5242	0.072*
C3′	0.3294 (9)	0.5755 (3)	0.55553 (10)	0.0622 (10)

C4′	0.5238 (9)	0.4942 (3)	0.55576 (10)	0.0650 (11)
H4′	0.6082	0.4733	0.5779	0.078*
C5′	0.5905 (7)	0.4444 (3)	0.52200 (10)	0.0551 (9)
C6′	0.4711 (7)	0.4746 (3)	0.48884 (9)	0.0518 (9)
H6′	0.5221	0.4404	0.4663	0.062*
C7′	0.1300 (8)	0.5941 (3)	0.45514 (10)	0.0601 (10)

Atomic displacement parameters (Å^2)

	U^{11}	U^{22}	U^{33}	U^{12}	U^{13}	U^{23}
C1	0.051 (2)	0.078 (2)	0.068 (2)	−0.005 (2)	0.003 (2)	−0.016 (2)
C2	0.086 (3)	0.083 (3)	0.097 (3)	−0.009 (3)	−0.009 (3)	−0.028 (3)
C3	0.084 (3)	0.069 (3)	0.120 (4)	−0.008 (3)	−0.026 (3)	−0.005 (3)
C4	0.059 (3)	0.079 (3)	0.106 (3)	−0.010 (2)	−0.010 (3)	0.028 (3)
C5	0.047 (2)	0.073 (2)	0.074 (2)	0.003 (2)	0.0025 (19)	0.012 (2)
C6	0.0348 (18)	0.067 (2)	0.054 (2)	0.0045 (17)	−0.0045 (16)	−0.0017 (17)
C9	0.063 (3)	0.102 (3)	0.060 (2)	−0.014 (2)	−0.009 (2)	−0.002 (2)
C7	0.0433 (19)	0.063 (2)	0.054 (2)	0.0036 (18)	−0.0063 (17)	0.0010 (16)
C8	0.049 (2)	0.080 (3)	0.058 (2)	0.002 (2)	−0.0079 (19)	0.007 (2)
C10	0.067 (2)	0.062 (2)	0.061 (2)	0.014 (2)	−0.008 (2)	0.0048 (18)
C11	0.094 (3)	0.088 (3)	0.063 (2)	−0.001 (3)	−0.010 (2)	0.008 (2)
C12	0.113 (4)	0.088 (3)	0.074 (3)	−0.004 (3)	−0.003 (3)	0.006 (2)
C13	0.127 (4)	0.105 (4)	0.076 (3)	−0.014 (3)	−0.010 (3)	0.028 (3)
C14	0.249 (9)	0.093 (4)	0.117 (4)	−0.019 (5)	0.014 (5)	0.012 (3)
O1′	0.0680 (17)	0.0637 (14)	0.0505 (13)	0.0126 (14)	−0.0048 (13)	−0.0066 (11)
O2′	0.120 (3)	0.089 (2)	0.0774 (17)	0.054 (2)	−0.0023 (18)	−0.0060 (16)
O3′	0.126 (3)	0.166 (4)	0.092 (2)	0.003 (3)	0.039 (3)	−0.042 (2)
O4′	0.186 (4)	0.104 (2)	0.0523 (17)	−0.020 (3)	−0.008 (2)	−0.0130 (16)
O5′	0.0633 (18)	0.0866 (19)	0.085 (2)	0.0119 (16)	0.0027 (16)	0.0022 (16)
O6′	0.109 (3)	0.136 (3)	0.084 (2)	0.029 (3)	−0.029 (2)	0.020 (2)
N1′	0.124 (4)	0.079 (2)	0.060 (3)	−0.032 (3)	0.021 (3)	−0.013 (2)
N2′	0.058 (2)	0.072 (2)	0.075 (2)	−0.0055 (18)	−0.011 (2)	0.0087 (19)
C1′	0.062 (2)	0.0384 (16)	0.053 (2)	−0.0085 (17)	0.0074 (18)	−0.0072 (15)
C2′	0.069 (2)	0.0455 (18)	0.066 (2)	−0.0153 (18)	0.012 (2)	−0.0062 (17)
C3′	0.077 (3)	0.058 (2)	0.052 (2)	−0.021 (2)	0.015 (2)	−0.0077 (18)
C4′	0.076 (3)	0.068 (2)	0.052 (2)	−0.029 (2)	−0.004 (2)	0.006 (2)
C5′	0.052 (2)	0.055 (2)	0.058 (2)	−0.0128 (19)	0.0001 (19)	0.0012 (18)
C6′	0.056 (2)	0.0494 (19)	0.050 (2)	−0.0092 (18)	0.0047 (18)	−0.0042 (16)
C7′	0.072 (3)	0.0462 (19)	0.062 (2)	0.008 (2)	0.000 (2)	−0.0016 (18)

Geometric parameters (Å, º)

C1—C2	1.513 (6)	C11—H112	0.9700
C1—C9	1.524 (5)	C12—C13	1.525 (7)
C1—C6	1.527 (4)	C12—H121	0.9700
C1—H1	0.9800	C12—H122	0.9700
C2—C3	1.476 (7)	C13—C14	1.469 (7)
C2—H21	0.9700	C13—H131	0.9700
C2—H22	0.9700	C13—H132	0.9700
C3—C4	1.319 (6)	C14—H141	0.9600
C3—H3	0.9300	C14—H142	0.9600
C4—C5	1.480 (6)	C14—H143	0.9600

C4—H4	0.9300	O1′—C7′	1.315 (4)
C5—C6	1.532 (5)	O2′—C7′	1.200 (4)
C5—H51	0.9700	O3′—N1′	1.212 (6)
C5—H52	0.9700	O4′—N1′	1.204 (5)
C6—C7	1.526 (4)	O5′—N2′	1.206 (4)
C6—H6	0.9800	O6′—N2′	1.212 (4)
C9—C8	1.513 (5)	N1′—C3′	1.479 (5)
C9—H91	0.9700	N2′—C5′	1.476 (5)
C9—H92	0.9700	C1′—C2′	1.380 (4)
C7—C10	1.500 (5)	C1′—C6′	1.391 (5)
C7—C8	1.553 (4)	C1′—C7′	1.488 (5)
C7—H7	0.9800	C2′—C3′	1.367 (5)
C8—C11	1.538 (5)	C2′—H2′	0.9300
C8—H8	0.9800	C3′—C4′	1.363 (5)
C10—O1′	1.461 (4)	C4′—C5′	1.375 (5)
C10—H101	0.9700	C4′—H4′	0.9300
C10—H102	0.9700	C5′—C6′	1.372 (5)
C11—C12	1.470 (6)	C6′—H6′	0.9300
C11—H111	0.9700		

C2—C1—C9	116.3 (3)	H101—C10—H102	108.5
C2—C1—C6	115.3 (3)	C12—C11—C8	116.4 (4)
C9—C1—C6	101.4 (3)	C12—C11—H111	108.2
C2—C1—H1	107.8	C8—C11—H111	108.2
C9—C1—H1	107.8	C12—C11—H112	108.2
C6—C1—H1	107.8	C8—C11—H112	108.2
C3—C2—C1	113.3 (4)	H111—C11—H112	107.3
C3—C2—H21	108.9	C11—C12—C13	113.8 (4)
C1—C2—H21	108.9	C11—C12—H121	108.8
C3—C2—H22	108.9	C13—C12—H121	108.8
C1—C2—H22	108.9	C11—C12—H122	108.8
H21—C2—H22	107.7	C13—C12—H122	108.8
C4—C3—C2	124.0 (4)	H121—C12—H122	107.7
C4—C3—H3	118.0	C14—C13—C12	112.2 (5)
C2—C3—H3	118.0	C14—C13—H131	109.2
C3—C4—C5	124.1 (4)	C12—C13—H131	109.2
C3—C4—H4	117.9	C14—C13—H132	109.2
C5—C4—H4	117.9	C12—C13—H132	109.2
C4—C5—C6	113.3 (3)	H131—C13—H132	107.9
C4—C5—H51	108.9	C13—C14—H141	109.5
C6—C5—H51	108.9	C13—C14—H142	109.5
C4—C5—H52	108.9	H141—C14—H142	109.5
C6—C5—H52	108.9	C13—C14—H143	109.5
H51—C5—H52	107.7	H141—C14—H143	109.5
C7—C6—C1	101.6 (3)	H142—C14—H143	109.5
C7—C6—C5	113.8 (3)	C7′—O1′—C10	115.7 (3)
C1—C6—C5	111.0 (3)	O4′—N1′—O3′	125.4 (4)
C7—C6—H6	110.0	O4′—N1′—C3′	117.6 (5)
C1—C6—H6	110.0	O3′—N1′—C3′	116.8 (5)
C5—C6—H6	110.0	O5′—N2′—O6′	124.9 (4)
C8—C9—C1	105.8 (3)	O5′—N2′—C5′	118.0 (3)
C8—C9—H91	110.6	O6′—N2′—C5′	117.1 (4)

C1—C9—H91	110.6	C2′—C1′—C6′	119.6 (3)
C8—C9—H92	110.6	C2′—C1′—C7′	118.2 (3)
C1—C9—H92	110.6	C6′—C1′—C7′	122.2 (3)
H91—C9—H92	108.7	C3′—C2′—C1′	119.5 (4)
C10—C7—C6	116.0 (3)	C3′—C2′—H2′	120.3
C10—C7—C8	112.6 (3)	C1′—C2′—H2′	120.3
C6—C7—C8	105.5 (3)	C4′—C3′—C2′	122.4 (3)
C10—C7—H7	107.4	C4′—C3′—N1′	118.8 (4)
C6—C7—H7	107.4	C2′—C3′—N1′	118.9 (4)
C8—C7—H7	107.4	C3′—C4′—C5′	117.5 (4)
C9—C8—C11	111.1 (3)	C3′—C4′—H4′	121.3
C9—C8—C7	104.7 (3)	C5′—C4′—H4′	121.3
C11—C8—C7	113.2 (3)	C6′—C5′—C4′	122.5 (4)
C9—C8—H8	109.2	C6′—C5′—N2′	118.0 (3)
C11—C8—H8	109.2	C4′—C5′—N2′	119.5 (3)
C7—C8—H8	109.2	C5′—C6′—C1′	118.6 (3)
O1′—C10—C7	107.5 (3)	C5′—C6′—H6′	120.7
O1′—C10—H101	110.2	C1′—C6′—H6′	120.7
C7—C10—H101	110.2	O2′—C7′—O1′	124.5 (4)
O1′—C10—H102	110.2	O2′—C7′—C1′	122.6 (3)
C7—C10—H102	110.2	O1′—C7′—C1′	113.0 (3)

C9—C1—C2—C3	−83.7 (5)	C11—C12—C13—C14	174.1 (6)
C6—C1—C2—C3	34.9 (5)	C7—C10—O1′—C7′	−173.8 (3)
C1—C2—C3—C4	−9.4 (7)	C6′—C1′—C2′—C3′	0.6 (5)
C2—C3—C4—C5	0.8 (7)	C7′—C1′—C2′—C3′	−179.8 (3)
C3—C4—C5—C6	−17.1 (6)	C1′—C2′—C3′—C4′	−1.0 (5)
C2—C1—C6—C7	−172.0 (4)	C1′—C2′—C3′—N1′	179.3 (3)
C9—C1—C6—C7	−45.5 (3)	O4′—N1′—C3′—C4′	18.2 (5)
C2—C1—C6—C5	−50.6 (4)	O3′—N1′—C3′—C4′	−164.8 (4)
C9—C1—C6—C5	75.9 (4)	O4′—N1′—C3′—C2′	−162.1 (4)
C4—C5—C6—C7	154.2 (3)	O3′—N1′—C3′—C2′	14.8 (6)
C4—C5—C6—C1	40.3 (4)	C2′—C3′—C4′—C5′	0.4 (5)
C2—C1—C9—C8	165.4 (4)	N1′—C3′—C4′—C5′	−180.0 (3)
C6—C1—C9—C8	39.5 (4)	C3′—C4′—C5′—C6′	0.7 (5)
C1—C6—C7—C10	160.6 (3)	C3′—C4′—C5′—N2′	179.9 (3)
C5—C6—C7—C10	41.1 (4)	O5′—N2′—C5′—C6′	11.9 (5)
C1—C6—C7—C8	35.2 (3)	O6′—N2′—C5′—C6′	−168.3 (3)
C5—C6—C7—C8	−84.2 (3)	O5′—N2′—C5′—C4′	−167.3 (3)
C1—C9—C8—C11	105.0 (4)	O6′—N2′—C5′—C4′	12.5 (5)
C1—C9—C8—C7	−17.6 (4)	C4′—C5′—C6′—C1′	−1.1 (5)
C10—C7—C8—C9	−138.6 (3)	N2′—C5′—C6′—C1′	179.7 (3)
C6—C7—C8—C9	−11.1 (4)	C2′—C1′—C6′—C5′	0.4 (5)
C10—C7—C8—C11	100.3 (4)	C7′—C1′—C6′—C5′	−179.2 (3)
C6—C7—C8—C11	−132.2 (3)	C10—O1′—C7′—O2′	−0.9 (5)
C6—C7—C10—O1′	58.6 (4)	C10—O1′—C7′—C1′	178.8 (3)
C8—C7—C10—O1′	−179.7 (3)	C2′—C1′—C7′—O2′	−1.8 (5)
C9—C8—C11—C12	−179.6 (4)	C6′—C1′—C7′—O2′	177.8 (4)
C7—C8—C11—C12	−62.2 (5)	C2′—C1′—C7′—O1′	178.4 (3)
C8—C11—C12—C13	−177.2 (4)	C6′—C1′—C7′—O1′	−2.0 (5)

References:

[1] Bruker, **2016**, *Bruker APEX3, SAINT+ and SADABS*, Bruker AXS Inc., Madison, Wisconsin, USA.

[2] G. Sheldrick, *Acta Crystallogr.*, **2015*a***, A**71**, 3.

[3] G. Sheldrick, *Acta Crystallogr.*, **2015*b***, C**71**, 3.

[4] C. F. Macrae, I. J. Bruno, J. A. Chisholm, P. R. Edgington, P. McCabe, E. Pidcock, L. Rodriguez-Monge, R. Taylor, J. van de Streek and P. A. Wood, *J. Appl. Crystallogr.*, **2008**, *41*, 466.

[5] F. J. Liotta, G. Van Duyne and B. K. Carpenter *Organometallics*, **1987**, *6*, 1010.

[6] J. Aube,; S. Ghosh and M. Tanol *J. Am. Chem. Soc.*, **1994**, *116*, 9009.

[7] Y. Nagao, Y. Hagiwara, T. Tohjo, Y. Hasegawa, M. Ochiai and M. Shiro *J. Org. Chem.*, **1988**, *53*, 5983.
